# Correction: Development and validation protocol for an instrument to measure household water insecurity across cultures and ecologies: the Household Water InSecurity Experiences (HWISE) Scale

**DOI:** 10.1136/bmjopen-2018-023558corr1

**Published:** 2019-09-24

Young SL, Collins SM, Boateng GO, *et al*. Development and validation protocol for an instrument to measure household water insecurity across cultures and ecologies: the Household Water InSecurity Experiences (HWISE) Scale. *BMJ Open* 2019;9:e023558. doi: 10.1136/bmjopen-2018-023558

This article was previously published with an error.

Four names were missed in the collaborators list. The names are Jonathan Maupin, Monet Niesluchowski, Asiki Gershim, and Divya Krishnakumar.

