# Supplementary material for: Development and validation protocol for an instrument to measure household water insecurity across cultures and ecologies: the Household Water InSecurity Experiences (HWISE) Scale
Source: BMJ Open. 2019 Jan 17;9(1):e023558. doi: 10.1136/bmjopen-2018-023558 (PMC6340431; doi:10.1136/bmjopen-2018-023558)
Supplement: Supplementary file 1 [file bmjopen-2018-023558supp001.pdf]

Supplemental Material 1. Overview of water insecurity survey items, by implementation phase.

	Module Version 1	Module Version 2	Rationale for Modifications to Module Version 1
**Response Options:**	Never (0), Rarely (1), Sometimes (2), Often (3), Always (4)	Never (0), Rarely (1), Sometimes (2), Often (3), Always (4)	
**Number of Questions:**	32	30	
**Dimensions:**	Multidimensional: psychological, disease, nutrition, economic, social	Multidimensional: psychological, disease, nutrition, economic, social, cultural	
**Domains**	**Module Version 1**	**Module Version 2**	
[redacted]	In the last 4 weeks, how frequently did you or anyone in your household **worry** you would not have **enough water for all of** your household needs?	In the last 4 weeks, how frequently did you or anyone in your household **worry** you would not have enough water for all of your household needs?	—
	In the last 4 weeks, how frequently have you or anyone in your household **worried about the safety** of the person getting water for your household?	In the last 4 weeks, how frequently have you or anyone in your household **worried about the safety of the person** getting water for your household?	—
	In the last 4 weeks, how frequently did you or anyone in your household **feel upset** about your water situation?	In the last 4 weeks, how frequently did you or anyone in your household feel **angry** about your water situation?	Word choice changed since upset does not translate well in many contexts and is tied to religiosity.
	In the last 4 weeks, how frequently has your or anyone in your household's **day been interrupted by your water situation**, including getting or distributing water within the household?	In the last 4 weeks, how frequently has you or anyone in your household had to **change schedules/plans** due to problems with your water situation, such as problems getting or distributing water within the household? Activities that may have been interrupted include caring for others, doing household chores, etc.	HWISE 1.0 items related to schedules subsumed into this question. Rephrased from "interrupted" to "changed schedules" because the former confused many participants in cognitive interviews.
		How **satisfied** are you with your water situation on a scale of 1-5? (1 is not at all satisfied and 5 is completely satisfied).	Starting with a question framed in the negative may bias people towards over-exaggerating their difficulties with water.
[redacted]	In the last 4 weeks, how frequently have problems with water prevented you or anyone in your household from **attending social events** (i.e. church, funerals, community gatherings, etc.)?	In the last 4 weeks, how frequently have problems with water prevented you or anyone in your household from **attending social or cultural events** (e.g. church, funerals, community gatherings, cultural practices, etc.)?	Revised to include cultural components of water.
	In the last 4 weeks, how frequently did the **children** in your household **miss school** because they were getting water?	In the last 4 weeks, how frequently did the children in your household **miss school** or **go to school late** because of problems with water (e.g. time spent fetching water, lack of water for bathing, etc.)?	In many contexts, children only miss part of the school day as a consequence of water problems (e.g. time spent fetching water, cleanliness). analyses.
	In the last 4 weeks, how frequently did you or anyone in your household have problems with water that caused **difficulties with neighbors** or others in the community?	In the last 4 weeks, how frequently did you or anyone in your household have problems with water that caused **difficulties with neighbors, water providers, or others** in the community?	In many contexts, individuals face difficulties with their water providers.
	In the last 4 weeks, how frequently did you or anyone in your household have problems with water that caused **difficulties within your household**?	In the last 4 weeks, how frequently did you or anyone in your household have problems with water that caused **difficulties within your household**?	—
		In the last 4 weeks, how frequently have you or anyone in your household been unable to access the **water that you preferred**?	Previous version did not captures the concept of accessing a dignified, culturally-preferred water source.
		In the last 4 weeks, how frequently have problems with water caused you or anyone in your household to **feel ashamed**/excluded/stigmatized?	There are many uses and dimensions of water; currently, we were previously lacking a specific cultural component.
[redacted]	In the last 4 weeks, how frequently has the **time spent getting** water prevented you or anyone in your household from **earning money** (e.g. engaging in paid work, economic activities)?	In the last 4 weeks, how frequently have problems with water prevented you or anyone in your household from **earning money** (e.g. engaging in paid work, economic activities)?	Changed language to be applicable for flood and drought conditions.
	In the last 4 weeks, how frequently have you or anyone in your household **lacked money needed** to buy water?	In the last 4 weeks, how frequently have you or anyone in your household **lacked money** needed to **buy water**?	—
	In the last 4 weeks, how frequently have you or anyone in your household **thought of leaving** [*name of town*] because there was no water there?	In the last 4 weeks, how frequently have you or anyone in your household thought of **moving dwellings** because of the water situation there?	Language deemed too extreme; people tend not to leave town since they have family nearby but *are* likely to switch households. Also, rephrased to say water situation instead of no water, e.g. in case of flooding or contaminated water.
	In the last 4 weeks, how frequently did you or anyone in your household want to **buy water but there was nowhere to buy it from**?	In the last 4 weeks, how frequently did you or anyone in your household want to buy water but there was **nowhere to buy it from**?	—
	In the last 4 weeks, how frequently have you or anyone in your household asked to **borrow water** from other people?	In the last 4 weeks, how frequently have you or anyone in your household asked to **borrow** water from other people?	—
	In the last 4 weeks, how frequently have you or anyone in your household **loaned water** to anyone?	In the last 4 weeks, how frequently have you or anyone in your household **loaned water** to anyone? **Only asked in a subset of HWISE 2.0 sites*	—
[redacted]	In the last 4 weeks, how frequently has the **time spent getting** water prevented you or anyone in your household from **caring for children in the household**?		Subsumed under "change schedules/plans" in HWISE 2.0.
	In the last 4 weeks, how frequently has the **time spent getting** water prevented you or anyone in your household from **doing household chores** (such as cooking, preparing food, washing clothes, etc.)?		Subsumed under "change schedules/plans" in HWISE 2.0.
	In the last 4 weeks, how frequently has there **not been enough** water in the household to **wash clothes**?	In the last 4 weeks, how frequently has there not been enough water in the household to **wash clothes**?	—
	In the last 4 weeks, how frequently have you or anyone in your household had to go **without washing hands** after **dirty activities** (e.g., defecating or changing diapers, cleaning animal dung) because you didn't have enough water?	In the last 4 weeks, how frequently have you or anyone in your household had to go **without washing hands** after **dirty activities** (e.g., defecating or changing diapers, cleaning animal dung) because of problems with water?	Changed language to be applicable for flood and drought conditions.
	In the last 4 weeks, how frequently have you or anyone in your household not had enough water to **wash the faces and hands of children in your household?**	In the last 4 weeks, how frequently have you or anyone in your household not **washed the faces and hands of children** because of problems with water?	Changed language to account for flood events.
	In the last 4 weeks, how frequently have you or anyone in your household had to go without **washing their body** because there wasn't enough water?	In the last 4 weeks, how frequently have you or anyone in your household had to go without **washing their body** because of problems with water (e.g. not enough water, dirty, unsafe)?	Changed language to be applicable for flood and drought conditions.
	In the last 4 weeks, how frequently did you or anyone in your household want to **treat your water**, but couldn't? By treat, I mean boiling, using chemicals to treat, or other ways you make your water safe to use or drink.		Not many people experienced this.
	In the last 4 weeks, how frequently have you or anyone in your household **drank water** that **tasted bad**?	In the last 4 weeks, how frequently have you or anyone in your household drank water that **looked, tasted, and/or smelled bad**?	Participants mentioned that water can taste, look, and smell bad; the question is more encompassing by including all of these organoleptic properties.
	In the last 4 weeks, how frequently have you or anyone in your household **actually drank water** that you thought was unsafe?	In the last 4 weeks, how frequently have you or anyone in your household drank water that you thought was **unsafe**?	"Actually" does not add anything to the question, therefore removed.
	In the last 4 weeks, how frequently have you or anyone in your household **not had enough water to take medications**?		Not enough people experienced this; too specific.
	In the last 4 weeks, how frequently have you or anyone in your household not gotten water where you wanted to because you were **too sick or weak** to get water?		Too rare.
[redacted]	In the last 4 weeks, how frequently have you or anyone in your household had to **change what was being eaten** because there wasn't enough water (e.g. for washing foods, cooking, etc.)?	In the last 4 weeks, how frequently have you or anyone in your household had to **change what was being eaten** because there were problems with water (e.g. for washing foods, cooking, etc.)?	Changed language to be applicable for flood and drought conditions.
	In the last 4 weeks, how frequently has there not been as much water to drink as you would like for you or anyone in your household?	In the last 4 weeks, how frequently has there not been **as much water to drink** as you would like for you or anyone in your household?	—
	In the last 4 weeks, how frequently have you or anyone in your household gone to **sleep thirsty**?	In the last 4 weeks, how frequently have you or anyone in your household gone to **sleep thirsty** because there wasn't any water to drink?	Ensures that the question is measuring lack of water.
	In the last 4 weeks, how frequently has there been **no water whatsoever** in your household?	In the last 4 weeks, how frequently has there been **no useable or drinkable water** whatsoever in your household?	Changed language to be applicable for flood and drought conditions.
[redacted]	In the last 4 weeks, how frequently has your household **not had enough** water for your **garden, crops, or fruit trees**?	In the last 4 weeks, how frequently has your household water situation impacted the cultivation of your **garden, crops, or fruit trees**?	Changed language to be applicable for flood and drought conditions.
	In the last 4 weeks, how frequently has your household **not had enough water** to give to your **animals and poultry?**	In the last 4 weeks, how frequently has your household water situation impacted your raising of **animals and poultry**?	Changed language to be applicable for flood and drought conditions.
[redacted]	In the last 4 weeks, how frequently has your household **water supply** from your main water source **been interrupted?**	In the last 4 weeks, how frequently has your household water supply from your main water source been **interrupted** or **limited** (e.g. water pressure, less water than expected)?	Include "limited" to cover instances where water is not available in normal or anticipated quantities.
