# Supplementary material for: Development and validation protocol for an instrument to measure household water insecurity across cultures and ecologies: the Household Water InSecurity Experiences (HWISE) Scale
Source: BMJ Open. 2019 Jan 17;9(1):e023558. doi: 10.1136/bmjopen-2018-023558 (PMC6340431; doi:10.1136/bmjopen-2018-023558)
Supplement: Supplementary file 2 [file bmjopen-2018-023558supp002.pdf]

HWISE 2.0 Consent Script, Survey, and Sources

Household Water Insecurity Experiences

Version 25, June 13, 2018

Household Water Insecurity Experiences

HWISE Consent Script

You are being asked to take part in a research study about the different factors affecting water insecurity and health for people in your area. We are asking you to take part because you live in this area and have unique insight into how water is acquired and used in your community.

What this study is about: The purpose of this study is to learn about the various ways people acquire and use water and any consequences that arise from household water insecurity. From this study, we will be able to develop a scale to measure household water insecurity in many settings across the world.

What we will ask you to do: If you agree to be in this study, we will ask you to participate in a brief survey during which we will ask you questions about the composition of your household, water acquisition and use, food insecurity, and stress.

Risks and benefits: There is the risk that you may find some of the questions about your household to be sensitive. There are no immediate benefits to you, but the information learned in this study will be useful for researchers who are studying household water insecurity. The information we learn through this survey may be shared with researchers at other institutions, but your personal identifying information will not be shared or linked to your responses.

Compensation. There is no compensation for taking part in this study.

Your answers will be confidential. We will write or record your responses to this survey using tablets, your name and information will not be included in any part of the survey. The written records and any information you share will be kept private. In any sort of report we make public we will not include any information that will make it possible to identify you. Research records will be kept in a locked file or in secure, password-protected online storage; only researchers will have access to the records.

Taking part is voluntary. Taking part in this study is completely voluntary. You may skip any questions that you do not want to answer. If you decide not to take part or to skip some of the questions, it will not affect your future relationship with Arizona State University or Northwestern University. If you decide to take part, you are free to withdraw at any time.

If you have questions: The lead researcher conducting this study is Dr. Sera Young at Northwestern University. If you have questions later, you can contact Dr. Young at sera.young@northwestern.edu. If you have any questions or concerns regarding your rights as a subject in this study, you may contact the Northwestern University Institutional Review Board (IRB) at +1 (847) 467-1723 or http://www.northwestern.edu.

Now I would like to ask if you agree to participate in this study.
Do you agree to participate in this study? **YES/NO**
Do you agree to allow me to record your responses to questions? **YES/NO**

Cross-cultural study of household water insecurity	
Interviewer_______________________________________	Date_________________
Time at start of interview___________________	Time at end of interview___________________
Country __________________	Region/District_________________
Neighborhood_________________	
Participant ID:______________	Language of interview: ________________
Participant gender: (0) Male (1) Female	
Participant place of residence: (0) Rural (2) Peri-urban (3) Urban	
Participant ethnicity:	
Participant ID should start with 1st two letters of country, eg. BA, NE, GU, GH…	

1. Screening Questions

Code Name	Questions	Coding Classification
SQ1a	Do you agree to participate in this survey?	0…No<br>1…Yes
SQ2	Are you 16 years of age or older?	0…No<br>1…Yes
SQ3	Would you consider yourself knowledgeable about water acquisition and use within your household?	0…No<br>1…Yes
SQ4	If no, who is most knowledgeable about water acquisition and use within your household? Who should I talk to?	

2. Socio Demographic Questions

SD1	Role in household: What is your relationship to the head of your household? By household, I mean all people who sleep under the same roof and take food from the same pot.	1….Self<br>2….Spouse/partner<br>3….Adult child<br>4….Other
SD2	What is the gender of household head?	0…Male<br>1…Female
SD9	What is your current relationship status?	1…Single/separated or divorced<br>2…Widowed<br>3…Have a partner who lives separately<br>4…Have a partner who lives with you<br>5…Married
SD3	How old are you?	
SD4	Who is primarily responsible for making sure there is enough water in the house? By household, I mean all people who sleep under the same roof and take food from the same pot.<br><br>*If primary responsibility is shared, choose (5) and then also circle the two categories of people who share the responsibility.*	1…Self<br>2…Spouse/partner<br>3…Children<br>4…Other family members<br>5…Shared responsibility

SD5	How many children (≤16 years) live in your household? By household, I mean all people who sleep under the same roof and take food from the same pot.	
SD6	How many adults (>16 years) including yourself live in your household? By household, I mean all people who sleep under the same roof and take food from the same pot.	
SD7	What type of housing do you live in?	1….House/condominium (owned)<br>2….House/condominium (rented)<br>3….Apartment (owned)<br>4…..Apartment (rented)<br>5….Farm (own)<br>6…..Farm (lease)<br>7…..Informal settlement/squatter community<br>8……Refugee/internally displaced person camp<br>9…..Other ____________________
SD8	What is your religion?	1….Christianity<br>2….Judaism<br>3….Islam<br>4…..Hinduism<br>5…..Buddhism<br>6…..Nonreligious<br>7…...Other
SD8a	What is your denomination?	

**3. Household Water Insecurity Experiences Scale (32 questions)**<br>*Now I'm going to ask you about your own personal experiences with water. For each of the items, please indicate how many times within the past 4 weeks or 30 days. Interviewer, please mark the response that best corresponds with the coding classification. For example, if participants says 15 times, you need to mark/circle code 4.*		
**Code name**	**Question**	**Coding Classification**
HWISE1	How **satisfied** are you with your water situation on a scale of 1-5? (1 is not at all satisfied and 5 is completely satisfied).	1…Not at all satisfied<br>2<br>3<br>4<br>5...Completely satisfied
HWISE2	In the last 4 weeks, how frequently have you or anyone in your household been unable to access the **water that you preferred**?	1...Never (0 times in the last 4 weeks)<br>2...Rarely (1–2 times in the last 4 weeks)<br>3...Sometimes (3–10 times in the last 4 weeks)<br>4...Often (11-20 times in the last 4 weeks)<br>5….Always (More than 20 times in the last 4 weeks)

		99….Don't know<br>88…Not applicable/I don't have this
HWISE3	In the last 4 weeks, how frequently did you or anyone in your household **worry** you would not have enough water for all of your household needs?	1...Never (0 times in the last 4 weeks)<br>2...Rarely (1–2 times in the last 4 weeks)<br>3...Sometimes (3–10 times in the last 4 weeks)<br>4...Often (11-20 times in the last 4 weeks)<br>5….Always (More than 20 times in the last 4 weeks)<br>99….Don't know<br>88…Not applicable/I don't have this
HWISE4	In the last 4 weeks, how frequently have you or anyone in your household **worried about the safety of the person** getting water for your household? *By getting, I mean: traveling to, collecting the water, and returning with the water.*	1...Never (0 times in the last 4 weeks)<br>2...Rarely (1–2 times in the last 4 weeks)<br>3...Sometimes (3–10 times in the last 4 weeks)<br>4...Often (11-20 times in the last 4 weeks)<br>5….Always (More than 20 times in the last 4 weeks)<br>99….Don't know<br>88…Not applicable/I don't have this
HWISE5	In the last 4 weeks, how frequently has your household water supply from your main water source been **interrupted** or **limited** (e.g. water pressure, less water than expected)?	1...Never (0 times in the last 4 weeks)<br>2...Rarely (1–2 times in the last 4 weeks)<br>3...Sometimes (3–10 times in the last 4 weeks)<br>4...Often (11-20 times in the last 4 weeks)<br>5….Always (More than 20 times in the last 4 weeks)<br>99….Don't know<br>88…Not applicable/I don't have this
HWISE5a	If your supply was interrupted or limited, were these **expected** (announced/scheduled) or unexpected?	0…Unexpected<br>1…Announced/Scheduled
HWISE6	In the last 4 weeks, how frequently has your household water situation impacted the cultivation of your **garden, crops, or fruit trees**?	1...Never (0 times in the last 4 weeks)<br>2...Rarely (1–2 times in the last 4 weeks)<br>3...Sometimes (3–10 times in the last 4 weeks)<br>4...Often (11-20 times in the last 4 weeks)<br>5….Always (More than 20 times in the last 4 weeks)<br>99….Don't know<br>88…Not applicable/I don't have this
HWISE7	In the last 4 weeks, how frequently has your household water situation impacted your raising of **animals and poultry**?	1...Never (0 times in the last 4 weeks)<br>2...Rarely (1–2 times in the last 4 weeks)<br>3...Sometimes (3–10 times in the last 4 weeks)<br>4...Often (11-20 times in the last 4 weeks)

		5….Always (More than 20 times in the last 4 weeks)<br>99….Don't know<br>88…Not applicable/I don't have this
HWISE8	In the last 4 weeks, how frequently have problems with water prevented you or anyone in your household from **earning money** (e.g. engaging in paid work, economic activities)?	1...Never (0 times in the last 4 weeks)<br>2...Rarely (1–2 times in the last 4 weeks)<br>3...Sometimes (3–10 times in the last 4 weeks)<br>4...Often (11-20 times in the last 4 weeks)<br>5….Always (More than 20 times in the last 4 weeks)<br>99….Don't know<br>88…Not applicable/I don't have this
HWISE9	In the last 4 weeks, how frequently have you or anyone in your household **lacked money** needed to **buy water**?	1...Never (0 times in the last 4 weeks)<br>2...Rarely (1–2 times in the last 4 weeks)<br>3...Sometimes (3–10 times in the last 4 weeks)<br>4...Often (11-20 times in the last 4 weeks)<br>5….Always (More than 20 times in the last 4 weeks)<br>99….Don't know<br>88…Not applicable/I don't have this
HWISE10	In the last 4 weeks, how frequently did you or anyone in your household want to buy water but there was **nowhere to buy it from**?	1...Never (0 times in the last 4 weeks)<br>2...Rarely (1–2 times in the last 4 weeks)<br>3...Sometimes (3–10 times in the last 4 weeks)<br>4...Often (11-20 times in the last 4 weeks)<br>5….Always (More than 20 times in the last 4 weeks)<br>99….Don't know<br>88…Not applicable/I don't have this
HWISE11	In the last 4 weeks, how frequently did the children in your household **miss school** or **go to school late** because of problems with water (e.g. time spent fetching water, lack of water for bathing, etc.)?	1...Never (0 times in the last 4 weeks)<br>2...Rarely (1–2 times in the last 4 weeks)<br>3...Sometimes (3–10 times in the last 4 weeks)<br>4...Often (11-20 times in the last 4 weeks)<br>5….Always (More than 20 times in the last 4 weeks)<br>99….Don't know<br>88…Not applicable/I don't have this
HWISE12	In the last 4 weeks, how frequently has there not been enough water in the household to **wash clothes**?	1...Never (0 times in the last 4 weeks)<br>2...Rarely (1–2 times in the last 4 weeks)<br>3...Sometimes (3–10 times in the last 4 weeks)<br>4...Often (11-20 times in the last 4 weeks)<br>5….Always (More than 20 times in the last 4 weeks)<br>99….Don't know<br>88…Not applicable/I don't have this

HWISE13	In the last 4 weeks, how frequently have you or anyone in your household had to **change what was being eaten** because there were problems with water (e.g. for washing foods, cooking, etc.)?	1...Never (0 times in the last 4 weeks)<br>2...Rarely (1–2 times in the last 4 weeks)<br>3...Sometimes (3–10 times in the last 4 weeks)<br>4...Often (11-20 times in the last 4 weeks)<br>5….Always (More than 20 times in the last 4 weeks)<br>99….Don't know<br>88…Not applicable/I don't have this
HWISE14	In the last 4 weeks, how frequently have you or anyone in your household had to go **without washing hands** after **dirty activities (**e.g., defecating or changing diapers, cleaning animal dung) because of problems with water?	1...Never (0 times in the last 4 weeks)<br>2...Rarely (1–2 times in the last 4 weeks)<br>3...Sometimes (3–10 times in the last 4 weeks)<br>4...Often (11-20 times in the last 4 weeks)<br>5….Always (More than 20 times in the last 4 weeks)<br>99….Don't know<br>88…Not applicable/I don't have this
HWISE15	In the last 4 weeks, how frequently have you or anyone in your household not **washed the faces and hands of children** because of problems with water?	1...Never (0 times in the last 4 weeks)<br>2...Rarely (1–2 times in the last 4 weeks)<br>3...Sometimes (3–10 times in the last 4 weeks)<br>4...Often (11-20 times in the last 4 weeks)<br>5….Always (More than 20 times in the last 4 weeks)<br>99….Don't know<br>88…Not applicable/I don't have this
HWISE16	In the last 4 weeks, how frequently have you or anyone in your household had to go without **washing their body** because of problems with water (e.g. not enough water, dirty, unsafe)?	1...Never (0 times in the last 4 weeks)<br>2...Rarely (1–2 times in the last 4 weeks)<br>3...Sometimes (3–10 times in the last 4 weeks)<br>4...Often (11-20 times in the last 4 weeks)<br>5….Always (More than 20 times in the last 4 weeks)<br>99….Don't know<br>88…Not applicable/I don't have this
HWISE17	In the last 4 weeks, how frequently has you or anyone in your household had to **change schedules/plans** due to problems with your water situation, such as problems getting or distributing water within the household? Activities that may have been interrupted include caring for others, doing household chores, etc.	1...Never (0 times in the last 4 weeks)<br>2...Rarely (1–2 times in the last 4 weeks)<br>3...Sometimes (3–10 times in the last 4 weeks)<br>4...Often (11-20 times in the last 4 weeks)<br>5….Always (More than 20 times in the last 4 weeks)<br>99….Don't know<br>88…Not applicable/I don't have this
HWISE18	In the last 4 weeks, how frequently have problems with water prevented you or anyone in your household from **attending social or cultural events**	1...Never (0 times in the last 4 weeks)<br>2...Rarely (1–2 times in the last 4 weeks)<br>3...Sometimes (3–10 times in the last 4 weeks)<br>4...Often (11-20 times in the last 4 weeks)

	(e.g. church, funerals, community gatherings, cultural practices, etc.)?	5….Always (More than 20 times in the last 4 weeks)<br>99….Don't know<br>88…Not applicable/I don't have this
HWISE19	In the last 4 weeks, how frequently have you or anyone in your household drank water that **looked, tasted, and/or smelled bad**?	1...Never (0 times in the last 4 weeks)<br>2...Rarely (1–2 times in the last 4 weeks)<br>3...Sometimes (3–10 times in the last 4 weeks)<br>4...Often (11-20 times in the last 4 weeks)<br>5….Always (More than 20 times in the last 4 weeks)<br>99….Don't know<br>88…Not applicable/I don't have this
HWISE20	In the last 4 weeks, how frequently have you or anyone in your household drank water that you thought was **unsafe**?	1...Never (0 times in the last 4 weeks)<br>2...Rarely (1–2 times in the last 4 weeks)<br>3...Sometimes (3–10 times in the last 4 weeks)<br>4...Often (11-20 times in the last 4 weeks)<br>5….Always (More than 20 times in the last 4 weeks)<br>99….Don't know<br>88…Not applicable/I don't have this
HWISE21c	If you needed to borrow water, from how many people could you borrow water?	
HWISE21	In the last 4 weeks, how frequently have you or anyone in your household asked to **borrow** water from other people?	1...Never (0 times in the last 4 weeks)<br>2...Rarely (1–2 times in the last 4 weeks)<br>3...Sometimes (3–10 times in the last 4 weeks)<br>4...Often (11-20 times in the last 4 weeks)<br>5….Always (More than 20 times in the last 4 weeks)<br>99….Don't know<br>88…Not applicable/I don't have this
HWISE21a	From whom? Please list all the ways you are connected to these people. For example, neighbor, family member, or both neighbor and family member.	Person #1:<br><br>Person #2:<br><br>Person #3:
HWISE21b	What were you expected to give in return?	
HWISE30	In the last 4 weeks, how frequently have you or anyone in your household loaned water to anyone?	1...Never (0 times in the last 4 weeks)<br>2...Rarely (1–2 times in the last 4 weeks)<br>3...Sometimes (3–10 times in the last 4 weeks)<br>4...Often (11-20 times in the last 4 weeks)<br>5….Always (More than 20 times in the last 4 weeks)

		99….Don't know<br>88…Not applicable/I don't have this
HWISE22	In the last 4 weeks, how frequently did you or anyone in your household have problems with water that caused **difficulties with neighbors, water providers, or others** in the community?	1...Never (0 times in the last 4 weeks)<br>2...Rarely (1–2 times in the last 4 weeks)<br>3...Sometimes (3–10 times in the last 4 weeks)<br>4...Often (11-20 times in the last 4 weeks)<br>5….Always (More than 20 times in the last 4 weeks)<br>99….Don't know<br>88…Not applicable/I don't have this
HWISE23	In the last 4 weeks, how frequently did you or anyone in your household have problems with water that caused **difficulties within your household**?	1...Never (0 times in the last 4 weeks)<br>2...Rarely (1–2 times in the last 4 weeks)<br>3...Sometimes (3–10 times in the last 4 weeks)<br>4...Often (11-20 times in the last 4 weeks)<br>5….Always (More than 20 times in the last 4 weeks)<br>99….Don't know<br>88…Not applicable/I don't have this
HWISE24	In the last 4 weeks, how frequently did you or anyone in your household feel **angry** about your water situation?	1...Never (0 times in the last 4 weeks)<br>2...Rarely (1–2 times in the last 4 weeks)<br>3...Sometimes (3–10 times in the last 4 weeks)<br>4...Often (11-20 times in the last 4 weeks)<br>5….Always (More than 20 times in the last 4 weeks)<br>99….Don't know<br>88…Not applicable/I don't have this
HWISE25	In the last 4 weeks, how frequently has there not been **as much water to drink** as you would like for you or anyone in your household?	1...Never (0 times in the last 4 weeks)<br>2...Rarely (1–2 times in the last 4 weeks)<br>3...Sometimes (3–10 times in the last 4 weeks)<br>4...Often (11-20 times in the last 4 weeks)<br>5….Always (More than 20 times in the last 4 weeks)<br>99….Don't know<br>88…Not applicable/I don't have this
HWISE26	In the last 4 weeks, how frequently have you or anyone in your household gone to **sleep thirsty** because there wasn't any water to drink?	1...Never (0 times in the last 4 weeks)<br>2...Rarely (1–2 times in the last 4 weeks)<br>3...Sometimes (3–10 times in the last 4 weeks)<br>4...Often (11-20 times in the last 4 weeks)<br>5….Always (More than 20 times in the last 4 weeks)<br>99….Don't know<br>88…Not applicable/I don't have this
HWISE27	In the last 4 weeks, how frequently has there been **no useable or drinkable water** whatsoever in your household?	1...Never (0 times in the last 4 weeks)<br>2...Rarely (1–2 times in the last 4 weeks)<br>3...Sometimes (3–10 times in the last 4 weeks)

		4...Often (11-20 times in the last 4 weeks)<br>5….Always (More than 20 times in the last 4 weeks)<br>99….Don't know<br>88…Not applicable/I don't have this
HWISE28	In the last 4 weeks, how frequently have you or anyone in your household thought of **moving dwellings** because of the water situation there?	1...Never (0 times in the last 4 weeks)<br>2...Rarely (1–2 times in the last 4 weeks)<br>3...Sometimes (3–10 times in the last 4 weeks)<br>4...Often (11-20 times in the last 4 weeks)<br>5….Always (More than 20 times in the last 4 weeks)<br>99….Don't know<br>88…Not applicable/I don't have this
HWISE29	In the last 4 weeks, how frequently have problems with water caused you or anyone in your household to **feel ashamed**/excluded/stigmatized?	1...Never (0 times in the last 4 weeks)<br>2...Rarely (1–2 times in the last 4 weeks)<br>3...Sometimes (3–10 times in the last 4 weeks)<br>4...Often (11-20 times in the last 4 weeks)<br>5….Always (More than 20 times in the last 4 weeks)<br>99….Don't know<br>88…Not applicable/I don't have this

**4. Water Access**<br>*Now I would like to learn how and where you acquire water.*		
Wat1	What is currently the **primary source** of drinking water for your household? (Choose only ONE)	1…Piped water<br>2…Stand pipe<br>3…Borehole/tubewell<br>4…Protected dug well<br>5…Unprotected dug well<br>6…Protected spring<br>7…Unprotected spring<br>8…Rainwater collection<br>9…Small water vendor<br>10…Tanker truck<br>11…Bottled water<br>12…Bagged/sachet water<br>13…Surface water (pond, river, lake)<br>14…Other person ____________<br>15…Other_________
Wat2	What is currently the **primary source** of non-drinking water for your household? (Choose only ONE)	1…Piped water<br>2…Stand pipe<br>3…Borehole/tubewell<br>4…Protected dug well<br>5…Unprotected dug well<br>6…Protected spring<br>7…Unprotected spring<br>8…Rainwater collection

		9…Small water vendor<br>10…Tanker truck<br>11…Bottled water<br>12…Bagged/sachet water<br>13…Surface water (pond, river, lake)<br>14…Other person ____________<br>15…Other_________
Wat3	How long (in minutes) does it take to go to the water source, get water and come back (including wait time)? *(If water source is in household/compound, record 00 minutes)*	____________ minutes
Wat4	How many trips in total are made to this site per week (not including household/compound?)?	__________ trips
Wat4a	Have you ever been injured while fetching water?	0...No<br>1…Yes
Wat4b	If so, how?	
Wat5	In the past 4 weeks, approximately how much money did you spend on getting water for your household?	Units: ______ Amount: ________
Wat6	In the past 4 weeks, was drinking water for your household typically treated in any way to make it safer?	0…No<br>1…Yes<br>DK…Don't know
Wat7	What is the primary way that your household treats your **drinking** water?	1…Do not treat it<br>2…Boil<br>3…Filter<br>4…Add chemicals<br>5…Other (Specify):<br>___________________________
Wat8	In the past 4 weeks, how much money did you spend to treat water (including money for chemicals to treat water)?	Currency: ______ Amount: ________

**5. Water Quantity, Utility and Stability**<br>*Now I would like to ask you questions on the quantity of water you have in your household and the amount you drink.*		
Wat9	What is the estimate of the current amount of drinking water (liters) stored in your household?	________ Liters
Wat10	What is the estimate of the current amount of non-drinking water (liters) stored in your household?	________ Liters
Wat11	Which of the months in the year does your household mostly experience **water shortage** (circle all months)?	1…January 7…July<br>2…February 8…August<br>3…March 9…September<br>4…April 10…October<br>5…May 11…November

		6…June 12…December
Wat12	Which of the months in the year does your household have **excessive amounts** of water (tick the specific months)?	1…January 7…July<br>2…February 8…August<br>3…March 9…September<br>4…April 10…October<br>5…May 11…November<br>6…June 12…December
Wat13	What times of day does your household experience water shortages?	0…None<br>1…Morning (Sunrise (6:00am) to 11:59am)<br>2…Afternoon (12:00 – 5:00pm)<br>3…Evening (5:01 – 8:00pm)<br>4…Night (8:01pm – 5:59am)
Wat14	What do you see as the main **cause of problems with water** in your area?	
Wat15	What do you do when you **don't have enough water** and don't have enough money to buy water?	

**6. Food Insecurity (HFIAS)**<br>*Now I'm going to ask you about your experiences with access to food in the last four weeks. I will ask you about the frequencies that you have experienced a few situations, and I want you to tell me how frequently it has happened in the last four weeks.*		
**Code Name**	**Questions**	**Coding Classification**
FI1	In the past four weeks, how frequently did you worry that your household would not have enough food?	1…Never (0 times in the last 4 weeks)<br>2…Rarely (1 – 2 times in the last 4 weeks)<br>3…Sometimes (3 – 10 times in the last 4 weeks)<br>4…Often (More than 10 times in the last 4 weeks)<br>9…Don't know
FI2	In the past four weeks, how frequently were you or any household member not able to eat foods you preferred because you couldn't obtain them because of a lack of resources (such as money, business, land, or any other thing that you would require to help you obtain other types of food)?	1…Never (0 times in the last 4 weeks)<br>2…Rarely (1 – 2 times in the last 4 weeks)<br>3…Sometimes (3 – 10 times in the last 4 weeks)<br>4…Often (More than 10 times in the last 4 weeks)<br>9…Don't know
FI3	In the past four weeks, how frequently did you or any household member have to eat a limited variety of food due to lack of resources (such as money, business, land, or any other thing that you would require to help you obtain other types of food)?	1…Never (0 times in the last 4 weeks)<br>2…Rarely (1 – 2 times in the last 4 weeks)<br>3…Sometimes (3 – 10 times in the last 4 weeks)<br>4…Often (More than 10 times in the last 4 weeks)<br>9…Don't know
FI4	In the past four weeks, how frequently did you or any household member have to eat some foods that you really did not want to eat because of a lack of resources to obtain other types of food?	1…Never (0 times in the last 4 weeks)<br>2…Rarely (1 – 2 times in the last 4 weeks)<br>3…Sometimes (3 – 10 times in the last 4 weeks)<br>4…Often (More than 10 times in the last 4 weeks)<br>9…Don't know
FI5	In the past four weeks, how frequently did	1…Never (0 times in the last 4 weeks)

	you or any household member have to eat a smaller meal than you felt you needed, meaning a little amount of food that did not satisfy you, because there was not enough food?	2…Rarely (1 – 2 times in the last 4 weeks)<br>3…Sometimes (3 – 10 times in the last 4 weeks)<br>4…Often (More than 10 times in the last 4 weeks)<br>9…Don't know
FI6	How many meals do you think you should eat in a day?	
FI7	In the past four weeks, how frequently did you or anyone in your household have to eat fewer meals in a day because there was not enough food?	1…Never (0 times in the last 4 weeks)<br>2…Rarely (1 – 2 times in the last 4 weeks)<br>3…Sometimes (3 – 10 times in the last 4 weeks)<br>4…Often (More than 10 times in the last 4 weeks)<br>9…Don't know
FI8	In the past four weeks, how frequently was there ever no food to eat of any kind in your household because of lack of resources to get food?	1…Never (0 times in the last 4 weeks)<br>2…Rarely (1 – 2 times in the last 4 weeks)<br>3…Sometimes (3 – 10 times in the last 4 weeks)<br>4…Often (More than 10 times in the last 4 weeks)<br>9…Don't know
FI9	In the past four weeks, how frequently did you or any household member go to sleep at night hungry because there was not enough food?	1…Never (0 times in the last 4 weeks)<br>2…Rarely (1 – 2 times in the last 4 weeks)<br>3…Sometimes (3 – 10 times in the last 4 weeks)<br>4…Often (More than 10 times in the last 4 weeks)<br>9…Don't know
FI10	In the past four weeks, how frequently did you or any household member go hungry for a whole day and night because of limited food in the house?	1…Never (0 times in the last 4 weeks)<br>2…Rarely (1 – 2 times in the last 4 weeks)<br>3…Sometimes (3 – 10 times in the last 4 weeks)<br>4…Often (More than 10 times in the last 4 weeks)<br>9…Don't know

**7. Perceived Stress Scale**<br>*The questions in this scale ask you about your feelings and thoughts during the last month. In each case, indicate how often you felt or thought a certain way.*		
PS1	In the past four weeks, how often have you felt that you were unable to control the important things in your life?	1…Never (0 times in the last 4 weeks)<br>2…Almost Never (1-2 times in the last 4 weeks)<br>3…Sometimes (3-10 times in the last 4 weeks)<br>4…Fairly Often (11-20 times in the last 4 weeks)<br>5…Very Often (More than 20 times in the last 4 weeks)
PS2	In the last month, how often have you felt confident about your ability to handle your personal problems?	1…Never (0 times in the last 4 weeks)<br>2…Almost Never (1-2 times in the last 4 weeks)<br>3…Sometimes (3-10 times in the last 4 weeks)<br>4…Fairly Often (11-20 times in the last 4 weeks)<br>5…Very Often (More than 20 times in the last 4 weeks)
PS3	In the last month, how often have you felt that things were going your way?	1…Never (0 times in the last 4 weeks)<br>2…Almost Never (1-2 times in the last 4 weeks)<br>3…Sometimes (3-10 times in the last 4 weeks)<br>4…Fairly Often (11-20 times in the last 4 weeks)<br>5…Very Often (More than 20 times in the last 4 weeks)
PS4	In the last month, how often have you felt difficulties were piling up so high that you could not overcome them?	1…Never (0 times in the last 4 weeks)<br>2…Almost Never (1-2 times in the last 4 weeks)<br>3…Sometimes (3-10 times in the last 4 weeks)

		4…Fairly Often (11-20 times in the last 4 weeks)<br>5…Very Often (More than 20 times in the last 4 weeks)

**8. Infant feeding**<br>*This question will ask you about infant feeding and how it can be impacted by the water situation in your area.*		
BF1	Can you tell me some ways that the water situation here affects how infants (under 12 months of age) are fed? (*Interviewer, prompt for three ways.)*	1.<br><br>2.<br><br>3.

**2a. Socio Demographic Questions (continued)**<br>*We are now going to ask you three final questions about your standing in your community.*		
SD8	What is your current occupation or how do you earn money?	
SD9	Here is a picture of a ladder. Please think of this ladder as representing the socioeconomic standing of people ***in your community.***<br><br>At the top of the ladder are those who are best off; they have the most money, the most education, the most respected jobs. At the bottom are people who have the least money, least education, and least jobs.<br><br>Where would you place yourself on this ladder? Please touch the rung/step.<br><br>Interviewer, write the corresponding number here: ________	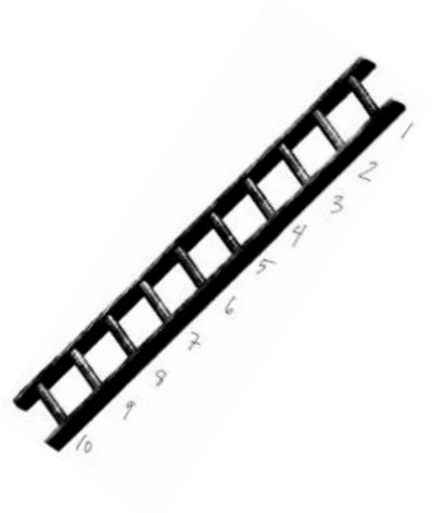

SD11	Here is a picture of a ladder. Please think of this ladder as representing the water situation of people ***in your community.***<br><br>At the top of the ladder are those who have the best water situation; they can easily get enough water for everything that they need and never have too much. At the bottom are people who have the most problems with water.<br><br>Where would you place yourself on this ladder? Please touch the rung/step.<br><br>Interviewer, write the corresponding number here: ________	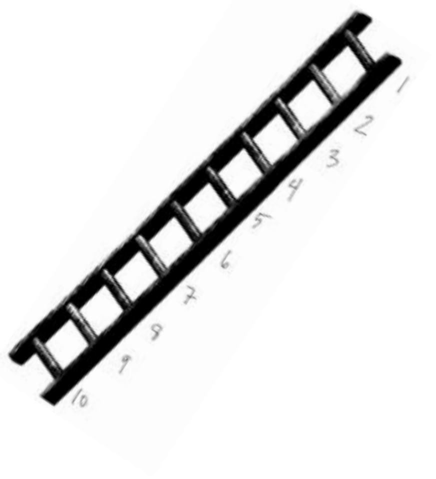
SD10	What is the primary monthly income for your household? (/month)	Units______ Amount ________

Wrap-up Question: Is there anything else you would like to share about your experiences with water for your household and how this affects your life?

Do you have any questions for us? ***Thank you*** *for participating in this survey.*

**Data Quality: For the interviewer to complete**<br>*Please give your assessment on the quality and reliability of the data you have collected in this survey.*		
DQ01	Did the respondent show any of the following? *(check all that apply):*	___ Mistrust of you or the study<br>___ Dishonesty, lying, or inventing responses that didn't seem true<br>___ Fear of you or the study<br>___ Hostility, anger or resentment<br>___ Evasion or trying to avoid answering
DQ02	Were there any interruptions or distractions?	0…No<br>1…Yes, but I don't think it influenced responses<br>2…Yes, and I think it influenced the answers
DQ03	What is your overall assessment of the quality of the data in this survey?	0…**Excellent**. The respondent understood the survey and was engaged and there were no interruptions<br>1…**Just okay**. The participant may not have understood all or part of the survey well or there were some interruptions.<br>2…**Suspect**. The participant misunderstood the survey or did not participate well or there were many interruptions.
DQ04	The quality of the data from this interview is really important to us. Please explain your answers above, in terms of how the data may have been affected.<br>Is there anything else we need to know?	

Household Water Insecurity Experiences

HWISE 2.0 Survey Sources

Topic	Brief Description	Source
**Socio-demographics**	Neighborhood, region, district/residence	Adapted from UNICEF Multiple Indicator Cluster Survey (MICS)[1]
	Role in household	
	Gender of Household head/respondent	
	Relationship status	Adapted from Demographic and Health Surveys (DHS) Household Questionnaire[2]
	Age of household head/respondent	
	Person responsible for collecting water in the household	Adapted from WHO & UNICEF Core Questions on Drinking Water and Sanitation for Household Surveys[3]
	Household size (# of adults & # of kids)	Adapted from UNICEF Multiple Indicator Cluster Survey (MICS)[1]
	Type of housing	Developed by investigators
	Religion and denomination	
**Household Water Insecurity Experiences (HWISE) Scale**	30-item household water insecurity experiences scale	Developed from extensive literature review[5] and team's knowledge of water insecurity[4,9-12]
**Water sharing**	Borrowing/loaning, what are you expected to give in return, from whom do you borrow?	Developed by investigators
**Water Quality**	Source of primary drinking water (WHO categories for improved & unimproved sources)	Adapted from WHO & UNICEF Core Questions on Drinking Water and Sanitation for Household Surveys[3]
	Source of primary non-drinking water (WHO categories for improved & unimproved sources)	
	Assessment of drinking water to be safe or unsafe	Developed by investigators
	Participants treating their water	Adapted from Demographic and Health Surveys (DHS) Household Questionnaire[2]
**Water Accessibility**	The amount of money spent by the household in water collection	Developed by investigators
	Estimate the time spent in collecting water from water source	Adapted from WHO & UNICEF Core Questions on Drinking Water and Sanitation for Household Surveys[3]
	Frequency of water collection	
**Water Quantity**	Amount of drinking water stored in household (L)	Developed by investigators
	Amount of non-drinking water stored in household (L)	
**Water Utility**	Amount of water drank in a day (L)	Developed by investigators
**Water Stability/Reliability**	Which of the months in a year do households experience water excess and scarcity?	Developed by investigators
	Which times of day do households experience water scarcity?	

**Food Insecurity**	9-item estimate via Household Food Insecurity Access Scale (HFIAS).	Adapted from the Household Food Insecurity Access Scale (HFIAS)[8]
**Perceived Stress**	4-item Estimate via Cohen's Perceived Stress Scale.	Adapted from the Perceived Stress Scale[7]
**Infant Feeding**	1 open-ended question on perceptions of how water insecurity may affect infant & young child feeding	Developed by investigators
**Socioeconomic Status**	Open-ended question about current occupation	Developed by investigators
	Likert ladder with degree of participants socio-economic status (scaled 1 to 10, with 1 being the best off, most educated, most money, and the most respected job; at the bottom participants with less money, education, least respected jobs)	Adapted from The MacArthur Scale of Subjective Social Status[8]
	Household monthly income	Developed by investigators
**Data Quality**	4-items on interviewer-assessed quality of responses	Developed by investigators

1. UNICEF. Multiple Indicator Cluster Survey (MICS): Household Questionnaire [Internet]. 2017. Available from: http://mics.unicef.org/tools#survey-design
2. The DHS Program, USAID. Demographic and Health Surveys (DHS): Questionnaire Modules [Internet]. 2015. Available from: https://dhsprogram.com/publications/publication-dhsqm-dhs-questionnaires-and-manuals.cfm
3. WHO, UNICEF. Core Questions on Drinking Water and Sanitation for Household Surveys [Internet]. Geneva : World Health Organization; 2006. Available from: https://extranet.who.int/iris/restricted/handle/10665/43489
4. Boateng G, Collins SM, Mbullo P, Wekesa P, Onono M, Neilands TB, et al. A Novel Household Water Insecurity Scale: Procedures and Psychometric Analysis Among Postpartum Women in Western Kenya. Biorxiv 10.1101/294298.
5. Jepson WE, Wutich A, Collins SM, Boateng GO, Young SL. Progress in household water insecurity metrics: a cross-disciplinary approach. WIREs Water. 2017 Apr 11;4(3):e1214-21.
6. Coates J, Swindale A, Bilinsky P. Household food insecurity access scale (HFIAS) for measurement of food access: Indicator guide. Washington DC: Food and Nutrition Technical Assistance Project; 2007.
7. Cohen S, Kamarck T, Mermelstein R. A global measure of perceived stress. J Health Soc Behav. 1983;24(4):385–96.
8. Adler N, Stewart J, Psychosocial Working Group. The MacArthur Scale of Subjective Social Status [Internet]. MacArthur Scale of Subjective Social Status. 2007. Available from: http://www.macses.ucsf.edu/research/psychosocial/subjective.php
9. Wutich A, Brewis A. Food, Water, and Scarcity. Current Anthropology. 2014;55(4):444–68.
10. Jepson W. Measuring no-win waterscapes: Experience-based scales and classification approaches to assess household water security in *colonias* on the US--Mexico border. Geoforum. 2014;51(C):107–20.
11. Wutich A, Ragsdale K. Water insecurity and emotional distress: coping with supply, access, and seasonal variability of water in a Bolivian squatter settlement. Social science & medicine. 2008;67(12):2116–25.
12. Krumdieck N, Collins S, Wekesa P, Mbullo P, Boateng G, Onono M, et al. Household water insecurity is associated with a range of negative consequences among pregnant Kenyan women of mixed HIV status. Journal of Water and Health. 2016 Jul 25.
