# Supplementary material for: Development and validation protocol for an instrument to measure household water insecurity across cultures and ecologies: the Household Water InSecurity Experiences (HWISE) Scale
Source: BMJ Open. 2019 Jan 17;9(1):e023558. doi: 10.1136/bmjopen-2018-023558 (PMC6340431; doi:10.1136/bmjopen-2018-023558)
Supplement: Supplementary file 3 [file bmjopen-2018-023558supp003.pdf]

Household Water Insecurity Experiences

HWISE Data Protocol

Data cleaning procedure and notes for using the HWISE data

Purposes of Data Cleaning

I. Identify Aberrant Data

A. There are numerous instances in which data may be entered incorrectly or missing when it should not be; for each of the following situations, the protocol is to 1) contact PIs to see if the data can be recovered and/or corrected and, if not, 2) either update the aberrant data with a reasonable value or as missing
   - Categorical variables that are not within reasonable or defined bounds (e.g. age is reported to be 500)
   - Data that are logically incongruent (e.g. respondent reports that he/she does not treat water but then lists a non-zero amount spent on water treatment)
   - Missing data

II. De-string Numeric Variables

A. String variables (i.e. those with with non-numeric characters) are updated to be numerical

III. Split Multiple-Select Variables

A. To make multiple-select responses analyzable, they are split into multiple responses (e.g. months of water shortage are split into 12 variables)

IV. Remove Duplicates

A. If multiple entries are submitted for the same participant, these are flagged and only the most up-to-date submission is retained

V. Recode Values

A. Values may be recoded to more clearly flag data that should later be changed to missing for analysis
   - 999 = "Don't know"
   - 888 = "Not applicable"
   - 555 = "Refuse to answer"

B. Multiple-select response options sometimes vary across sites (e.g. '1' is piped water in one site but '7' in another); to account for this in aggregated datasets, values are recoded to be harmonious across sites

VI. Label Data

A. Add variable labels to describe what a variable represents

B. Add value labels to categorical variables to convey what each value represents (e.g. ‘0’ is male)

VII. Generate New Variables

A. Variables that are of broad interest to the group are generated based on other items within the dataset (e.g. creating a food insecurity score based on the FIAS questions)

VIII. Reorder Variables

A. Throughout the data entry and cleaning process, variables may be shifted around; these are reordered to match the order in the original survey

Stages of Data Cleaning

I. Cleaning of Site-Specific Data

A. Raw .csv data is imported and saved as a Stata dataset (.dta file)
B. All variables are then cleaned following the above guidelines; this includes variables unique to specific sites (e.g. ward in Ethiopia)
  - All changes made to aberrant data are documented in the data dictionary (see below)

C. Once cleaned, the dataset is saved

II. Cleaning of Aggregated Data

A. Cleaned datasets for each site are appended together
B. Site-specific variables are dropped
C. Multiple-select response options that vary across sites (e.g. ‘1’ is piped water in one site but ‘7’ in another) are recoded so responses across all sites are comparable
D. Questions related to money are converted to USD (exchange rate for each site based on date of last interview at the site and pulled from https://www.oanda.com/currency/converter/)
E. Once cleaned, the dataset is saved
F. Code replacing 555, 888, 999 with missing is then executed; the dataset is saved again

Using HWISE Data

I. Data Dictionary

A. The first tab (“Summary”) provides a broad overview of the modules within the survey; blue text hyperlinks to the respective module in the more in-depth “Dictionary” tab
B. The “Dictionary” tab describes what each variables represents, data type, and the appropriate range of responses
C. The “Data Errors” section lists each error encountered while cleaning the site-specific data, as well as any corrective action taken; if you discover an error in the data that is not listed here, please contact the Northwestern Team

II. Determining Which Aggregated Dataset to Use

A. One dataset retains 555 (refuse to answer), 888 (not applicable), and 999 (don’t know); this is best used for understanding the range of responses
B. The other dataset replaces 555, 888, and 999 with missing; it is best to use this dataset when performing analyses
