# Supplementary material for: Development and validation protocol for an instrument to measure household water insecurity across cultures and ecologies: the Household Water InSecurity Experiences (HWISE) Scale
Source: BMJ Open. 2019 Jan 17;9(1):e023558. doi: 10.1136/bmjopen-2018-023558 (PMC6340431; doi:10.1136/bmjopen-2018-023558)
Supplement: Supplementary file 4 [file bmjopen-2018-023558supp004.pdf]

Supplemental Material 4. Interviewer debriefing guide.

Cross-cultural study of Household Water Insecurity

Interviewer Debriefing Guide

Interviewer ID ____________________

Country __________________________ **Region/District**_________________

Gender of interviewer (participant): (0) Male (1) Female

Interviewer (participant) conducted interviews/surveys in what kind of area(s):
(0) Rural (2) Peri-urban (3) Urban

0a. This debrief is occurring after the completion of the:
(1) Cognitive interviews (2) Surveys

0b. Approximately how many of the above (surveys OR interviews) did you conduct? ________

0c. What was the primary language(s) you conducted the above (surveys or interviews) in? ____

1. What questions on the water insecurity access scale (WIAS) do you think worked best, and why?

2. Which questions on the water insecurity access scale (WIAS) were not well understood by participants? Why do you think they were hard to understand?

3. What questions didn't work in this population, and why?

4. What do you think was the most important question in understanding water insecurity here and why?

5. What question(s) do you wish we would have asked to better understand water insecurity?
