# Supplementary material for: Development and validation protocol for an instrument to measure household water insecurity across cultures and ecologies: the Household Water InSecurity Experiences (HWISE) Scale
Source: BMJ Open. 2019 Jan 17;9(1):e023558. doi: 10.1136/bmjopen-2018-023558 (PMC6340431; doi:10.1136/bmjopen-2018-023558)
Supplement: Author's manuscript [file bmjopen-2018-023558.draft_revisions.pdf]

BMJ Open is committed to open peer review. As part of this commitment we make the peer review history of every article we publish publicly available.

When an article is published we post the peer reviewers' comments and the authors' responses online. We also post the versions of the paper that were used during peer review. These are the versions that the peer review comments apply to.

The versions of the paper that follow are the versions that were submitted during the peer review process. They are not the versions of record or the final published versions. They should not be cited or distributed as the published version of this manuscript.

BMJ Open is an open access journal and the full, final, typeset and author-corrected version of record of the manuscript is available on our site with no access controls, subscription charges or pay-per-view fees (http://bmjopen.bmj.com).

If you have any questions on BMJ Open's open peer review process please email info.bmjopen@bmj.com

BMJ Open

A protocol for the development and validation of an instrument to measure household water insecurity across cultures: the Household Water InSecurity Experiences (HWISE) scale

Journal:	*BMJ Open*
Manuscript ID	bmjopen-2018-023558
Article Type:	Protocol
Date Submitted by the Author:	11-Apr-2018
Complete List of Authors:	Young, Sera; Northwestern University, Anthropology<br>Collins, Shalean ; Northwestern University, Anthropology<br>Boateng, Godfred; Northwestern University, Anthropology<br>Neilands, Torsten; University of California San Francisco, School of Medicine<br>Jamaluddine, Zeina; American University of Beirut, Center for Research on Population and Health<br>Miller , Joshua; Northwestern University, Anthropology<br>Brewis, Alex; Arizona State University, School of Human Evolution and Social Change<br>Frongillo, EA; University of South Carolina, Department of Health Promotion, Education, and Behavior<br>Jepson, Wendy; Texas A&M University Department of Geography<br>Melgar-Quiñonez, Hugo; McGill University Institute, Institute for Global Food Security<br>Schuster, Roseanne; Arizona State University, School of Human Evolution and Social Change<br>Stoler, JB; University of Miami, Department of Geography<br>Wutich, Amber; Arizona State University, School of Human Evolution and Social Change
Keywords:	water security, survey, water, household, scale development

SCHOLARONE™
Manuscripts

A protocol for the development and validation of an instrument to measure household water insecurity across cultures: the Household Water InSecurity Experiences (HWISE) Scale

Sera L. Young[1], Shalean M Collins[1], Godfred O. Boateng[1], Torsten B. Neilands[2], Zeina Jamaluddine[3], Joshua D. Miller[1], Alexandra Brewis[6], Edward A. Frongillo[5], Wendy E. Jepson[8], Hugo Melgar-Quiñonez[4], Roseanne C. Schuster[6], Justin Stoler[7], Amber Wutich[6]

[1]Northwestern University, Department of Anthropology & Institute for Policy Research, Evanston, IL
[2]University of California, San Francisco, School of Medicine, San Francisco, CA US
[3]Center for Research on Population and Health, American University of Beirut, Beirut, Lebanon
[4]Institute for Global Food Security, McGill University, Montreal, QC
[5]University of South Carolina, Department of Health Promotion, Education, and Behavior, USA
[6]Arizona State University, School of Human Evolution and Social Change, Tempe, AZ 85284 USA
[7]University of Miami, Miami, FL USA
[8]Texas A&M University, College Station, TX USA

Corresponding Author
Sera L. Young
1819 Hinman Ave
Evanston, IL 60208
Sera.young@northwestern.edu

For peer review only - http://bmjopen.bmj.com/site/about/guidelines.xhtml

ABSTRACT

Introduction: A wide range of water-related problems contribute to a significant global health burden. Despite the many plausible consequences for health and wellbeing, there is currently no validated way to measure water insecurity equivalently at the individual- or household-levels across cultural and geographic settings. Therefore, we are setting out to develop the Household Water InSecurity Experiences (HWISE) Scale for measuring household-level water insecurity in multiple contexts.

Methods and analysis: After domain specification and item development, items will be assessed for both content and face validity. Identified items will be implemented in at least 24 sites globally where water-related problems have been reported (e.g. shortages, excess water, issues with quality), with a minimum of 250 individuals at each site. Scale development will draw on analytic methods from both Classic Test and Item Response Theories, and include item reduction and factor structure identification and confirmation. Scale evaluation will entail assessments of reliability, and predictive, convergent, and discriminant validity, as well as the assessment of differentiation across known groups.

Ethics and dissemination: Study activities will receive necessary ethical approvals from academic and national scientific review bodies relevant to each site. We anticipate that the final HWISE scale will be completed by late 2018 and will be made available through open-access Creative Commons licensing. Associated findings will be disseminated to scientists, practitioners, and policy makers through peer-reviewed journals and conference presentations. Measures to quantify household food insecurity have been transformative for policy, research, and humanitarian aid efforts globally, and we expect that an analogous measure for water insecurity will achieve similar impacts.

For peer review only

For peer review only - http://bmjopen.bmj.com/site/about/guidelines.xhtml

Keywords: water security, survey, water, household, scale development

Article Summary
*This study is based on rigorous, multi-disciplinary formative research on water insecurity by anthropologists, geographers, nutritionists, and epidemiologists, among others.
Strengths and limitations of this study
*A further strength is that data on household water insecurity experiences are being collected in dozens of sites across four continents by local partners in widely varying cultural, ecological, and political settings.
*We will use analytic methods from both Classic Test and Item Response Theories to develop and evaluate the eventual scale.
* The HWISE scale is validated for assessing water insecurity in low- and middle-income countries only. Additional scale assessment relevant to high income countries is planned.

3507 words

Introduction

Water insecurity, the inability to "access and benefit from affordable, adequate, reliable and safe water for wellbeing and a healthy life",[1] has manifold adverse effects on physical and mental health;[2,3] undermines productivity;[4] triggers and perpetuates domestic, social, and political tensions and conflicts;[5,6] and reinforces environmental and social inequities.[7] Water insecurity often co-occurs with food insecurity, malnutrition, and communicable diseases, and can produce syndemic, or systemically exacerbating, epidemics[8,9] much like food insecurity and HIV.[10] Further, water insecurity is projected to worsen in many regions due to climate change and increased inequalities in resource distribution.[7]

Currently, we do not know with any precision how many households or individuals globally are affected by water insecurity, although satellite imagery-derived estimates of available surface water suggest that 4 billion people worldwide experience severe water scarcity at least one month of every year.[11] Measures of water availability exist at the national, regional, and community levels, and are often referred to as indicators of water scarcity, water poverty or water security.[12–15] However, they do not capture the granularity of experiences of water

For peer review only - http://bmjopen.bmj.com/site/about/guidelines.xhtml

insecurity, perceptions of quality, or instances of water excess. Further, there is not a common definition of water insecurity, and thus, the ability to compare measures of water insecurity is limited. While household-level scales have been developed in several sites, e.g. Texas,[16] Bolivia,[17] Uganda,[18] Ethiopia,[19] and Kenya,[8] their comprehensiveness and applicability to other sites is unknown and possibly limited.

As such, a comprehensive, validated scale to measure the experiences of household or individual water insecurity would enable researchers, practitioners, and policymakers to improve estimates of water insecurity prevalence, identify the factors that shape this phenomenon, understand how to more effectively distribute resources, and evaluate the impacts of interventions at a micro level.

Analogous measures of household food insecurity (e.g., Latin American and Caribbean Food Security Scale,[20] Household Food Insecurity Access Scale,[21] Food Insecurity Experience Scale[22]) have been available for some time, and have proven to be transformative for policy and programs. As such, development of an analogous water insecurity scale is overdue and desperately needed, particularly for assessing water insecurity in low- and middle-income settings where household water shortages tend to be most pronounced and frequent.

Therefore, we seek to develop and validate the first household water insecurity scale appropriate for use across low- and middle-income settings. The Household Water InSecurity Experiences (HWISE, pronounced *H-wise*) Consortium (www.sites.northwestern.edu/hwise) was formed as the platform for the multi-country, collaborative data collection required to validate the planned tool ("the HWISE Scale"). The HWISE Consortium brings together a large team of anthropologists, geographers, public health practitioners, physicians, epidemiologists, sociologists, nutritionists, *inter alia*, all of whom have experience with water insecurity, food

For peer review only - http://bmjopen.bmj.com/site/about/guidelines.xhtml

insecurity, and/or scale development across a wide array of low- and middle-income settings (https://sites.northwestern.edu/hwise/collaborators/).

Methods and Analysis

Phase 1: Domain identification

Specifying the domains for the scale is the first step in scale development (Table 1).[23] The boundary of the domain of water insecurity, i.e. the underlying construct that the scale will attempt to measure, is based on extensive literature review[1] and draws on the team's expertise in water insecurity e.g.[6,16,17,24]. As such, we define water insecurity as the condition where "affordability, reliability, adequacy, and safety [of water] is significantly reduced or unattainable so as to threaten or jeopardize well-being".[1]

A best practice is to clearly articulate sub-domains of the eventual scale, if they are known.[25] Although some proposed sub-domains of water insecurity have been proposed,[1,9,19,26] there is currently no consensus on what those are, so we will review potential sub-domains as the scale is developed.

Table 1. Overview of planned methods and analyses for the development of the HWISE scale

Scale Development Activity	Procedure
**Phase 1: Item development**	
Domain specification	Literature review
Item generation	Literature review
Content validity	By target population: Two styles of cognitive interviews were used in the first 12 sites, building on Delphi methodology from prior work
Face validity	Pre-testing and debriefing with enumerators at each site
**Phase 2: Scale development***	
Item reduction	Drop items with cumulative missing cases >30%
	Assess the performance of each item's variation with other items in the scale using a correlation matrix; items with very low (<0.30) inter-item correlation coefficients and very low (<0.30) item-total correlation coefficients across multiple sites will be considered for deletion, as will items that misfit the model, i.e. with residual correlations >0.2
	Item reduction in Rasch paradigm: item severity, item discrimination test

For peer review only - http://bmjopen.bmj.com/site/about/guidelines.xhtml

Identify factor structure	Use factorial analysis across multiple sites to test for factor structure; items with very low factor loadings (<0.30), split factor loadings (high factor loadings in two domains), and high residual variances will be considered for deletion
Assess measurement equivalence	Using multi-group confirmatory analysis (a form of measurement invariance) on data from multiple sites to test for invariance in the hypothetical scale; invariance will be assessed in terms of factor structure (configural model), factor loadings (matrix model), mean intercepts (scalar model), and factor means (strict model)
	Use confirmatory factor analysis alignment optimization to estimate the group specific factor means and variances of scale items across all sites
**Phase 3: Scale evaluation***	
Score scale items	Finalized scale items will be used in their unweighted form as sum scores, or in weighted form as factor scores
Assess reliability (internal consistency) of scale items	We will use Cronbach's alpha and the Rasch reliability statistic to test the internal consistency of the scale items within each site and aggregated across sites

Item generation

We identified initial scale items deductively based on an extensive literature review of items applied in the development of prior site-specific household water insecurity scales (cf. Supplementary Table A[1]). This included team members' prior work implemented in *colonias* in Texas;[16] a squatter settlement in Cochabamba, Bolivia;[17] in rural, peri-urban, and urban Kenya;[8] and elsewhere, including rural areas in Ethiopia[19] and Uganda.[18] These items were expanded based on expert input and will be adapted to be maximally appropriate in a cross-cultural interrogation of household water insecurity through three successive rounds of data collection across multiple country sites (Supplemental Material 1: HWISE 1.0, 2.0, and 3.0 items). Emerging sub-domains are psychosocial and physical health, nutrition, impacts on livelihoods and household economy, and agriculture.

Each individual item is phrased to elicit experiences within the prior four weeks or month. This recall period was determined systematically using the Delphi method of consensus building and by comparing the responses in this recall period to a prospective daily recall of water insecurity experiences.[8] Items are ordered by what we expect to be increasing severity of water insecurity across access, reliability, adequacy, and safety. Response options are "never" (0

For peer review only

For peer review only - http://bmjopen.bmj.com/site/about/guidelines.xhtml

times), “rarely” (1-2 times), “sometimes” (3-10 times), “often” (11-20 times), “always” (more than 20 times), “not applicable”, “don’t know”, or refused (Supplemental Material 1: HWISE 1.0, 2.0, and 3.0 items).

Study populations

Sites

Initially, six sites were selected for data collection: Bangladesh, Brazil, Guatemala, Kenya, Nepal, and Tajikistan. However, the addition of more sites will allow us to test the instrument across more heterogeneous cultural and geographic settings. Additional sites will also permit an iterative analysis of the instrument (cf. “Mid-study evaluation”). Sites are added by leveraging professional networks across academia as well as non-governmental and governmental organizations. In addition to seeking maximal heterogeneity of sites, cost and feasibility of timely implementation are key considerations for site additions. The target is to have at least 24 sites across diverse settings (e.g., both urban and rural, formal and informal settlements) in areas thought to experience either seasonal or chronic forms of water insecurity (Figure 1; Table 2). Such a large number of sites will allow the cross-cultural validation of the scale items using multiple analytical methods.[27]

For peer review only

For peer review only - http://bmjopen.bmj.com/site/about/guidelines.xhtml

Table 2. Characteristics of HWISE sites for scale development, by region

World Bank Region	Site, (HWISE survey version)	Main climate, precipitation (Köppen climate type)[1]	GNI per capita (USD)[3]	Income Classification[2]	Setting
Africa	Accra, Ghana (1.0)	Equatorial, winter dry (AW)	1380	Lower middle income	Urban
	Lagos, Nigeria 1.0)	Equatorial, winter dry (Aw)	2450	Lower middle income	Urban
	Kahemba, DRC (1.0)	Equatorial, winter dry (Aw)	420	Low income	Rural
	Bahir Dar, Ethiopia (1.0)	Warm temperature, winter dry, warm summer (Cwb)	660	Low income	Rural
	Singida, Tanzania (1.0)	Equatorial, winter dry (Aw)	900	Low income	Rural
	Lilongwe, Malawi (1.0)	Warm temperature, winter dry, hot summer (Cwa)	320	Low income	Peri-urban
	Arua, Uganda (1.0)	Equatorial, winter dry (Aw)	660	Low income	Rural
	Seme, Kenya (1.0)	Equatorial, fully humid (Af)	1380	Lower middle income	Rural
	Kampala, Uganda (1.0)	Equatorial, winter dry (Aw)	660	Low income	Urban
	Morogoro, Tanzania (3.0)	Equatorial, winter dry (Aw)	900	Low income	Rural and peri-urban
	Kisoro, Uganda (3.0)	Warm temperature, fully humid, warm summer (Cfb)	490	Low income	Rural
East Asia and Pacific	Upolu, Samoa (1.0)	no data	4100	Upper middle income	TBD
	Labuan Bajo, Indonesia (3.0)	Equatorial, fully humid (Af)	3400	Lower middle income	TBD
Europe & Central Asia	Dushanbe, Tajikistan (1.0)	Warm temperature, summer dry, warm summer (Csb)	1110	Lower middle income	Urban
Latin America and the Caribbean	Pecem & Ceará, Brazil (1.0)	Equatorial, summer dry (As)	8840	Upper middle income	Urban
	Merida, Mexico (1.0)	Arid steppe, hot arid (BSh)	9040	Upper middle income	Urban
	Acatenango, Guatemala (1.0)	Warm temperature, winter dry, warm summer (Cwb)	3790	Lower middle income	Peri-urban
	Honda, Colombia (1.0)	Equatorial, fully humid (Af)	6320	Upper middle income	Peri-urban
	San Borja, Bolivia (1.0)	Equatorial, monsoonal (Am)	3070	Lower middle income	Rural
	Chiquimula, Guatemala (2.0)	Equatorial, monsoonal (Am)	3790	Lower middle income	Rural
	Gressier, Haiti (2.0)	Equatorial, winter dry (Aw)	780	Low income	Rural
Middle East and North Africa	Beirut, Lebanon (2.0)	Warm temperature, summer dry, hot summer (Csa)	7680	Upper middle income	Urban
South Asia	Kathmandu, Nepal (1.0)	Warm temperature, winter dry, hot summer (Cwa)	730	Low income	Urban
	Pune, India (1.0)	Equatorial, winter dry (Aw)	1680	Lower middle income	Urban
	Hajipur, Pakistan (2.0)	Arid, desert, hot arid (BWh)	1510	Lower middle income	Rural
	Rajasthan, India (3.0)	Arid steppe, hot arid (BSh)	1680	Lower middle income	Urban
	Dhaka and Chakaria, Bangladesh (3.0)	Equatorial, winter dry (Aw), Equatorial, monsoonal (Am)	1330	Lower middle income	Urban

[1]Köppen climate classification predicted using Scenario A1F1 for 2001-2025, projected to December 31, 2020 used for reference point (ESRI, ArcGIS)
[2]Income Classification from World Bank, data from 2017
[3]Gross National Income in USD from World Bank classification, data from 2017

For peer review only - http://bmjopen.bmj.com/site/about/guidelines.xhtml

Participant selection

Participants must be 16 or 18 years of age or older (depending on age of consent in each site), identify themselves to the interviewer as the person most knowledgeable about water acquisition and use within their households, and consent to participate. Participants are not remunerated for participation in the survey.

The target sample size at each site is 250 individuals. This sample size is designed to facilitate assessment of the magnitude of the correlation between the observed variables and the components/factors, and obtain a sample pattern that is stable and approximates the population pattern.[28] If sites cannot achieve the target sample size, variation of estimated statistics will be reviewed to determine if the data can be included in final validation of the scale.

The preferred sampling strategy for the study is random sampling of mutually exclusive and exhaustive categories of participants in areas of known high, moderate, and low water insecurity. In standalone HWISE surveys, participant selection follows a simple randomized or cluster-randomized sampling strategy (Table 3). In several sites, the HWISE survey is administered as part of a larger ongoing project with a predetermined survey design (e.g., in Singida, Tanzania, clinicaltrials.gov: NCT02761876 and Kahemba, Democratic Republic of Congo: NCT03157336), such that simple random sampling is not possible.

Sites with simple randomized sampling employ a random-walk sampling method.[29] With the simple randomized sampling strategy, a random number generator (e.g. dice or random number generating application) with set parameters (i.e. less than 20, less than 30, etc.) determines which

For peer review only - http://bmjopen.bmj.com/site/about/guidelines.xhtml

households are surveyed. Surveys are administered to each household corresponding to the random number, such that a random draw of the number 3 indicates that every third household should be sampled. Sites using cluster-randomized sampling first map regions using a grid or satellite imagery (e.g. Google Maps) to identify population density based on the number of habitable structures. Clusters are selected from this grid, and households within clusters are randomly sampled in proportion to structure or population density using a random number generator, similar to the simple randomized sampling. Cluster-randomization is preferred, but simple random sampling is used when cluster data are not available, typically in sparsely populated settings.

For peer review only

For peer review only - http://bmjopen.bmj.com/site/about/guidelines.xhtml

Table 3. Overview of data collection activities at each site

World Bank Region	Site and survey version	Implementing partners	Details of larger study	IRB of record	Season of data collection	Date of data collection	Sampling strategy	Language(s) of data collection	Data collection method (software)	Cognitive interviewing	n=
Africa	Accra, Ghana (1.0)	University of Miami, Delaware State University, Ghana Water Company	Standalone	Miami and Delaware reliant on Northwestern, Ghana Water Company	Rainy season	May 2017	Cluster random	English	Tablet (ODK)	Yes	237
	Lagos, Nigeria (1.0)	College of Medicine at the University of Lagos, Northwestern University	Standalone	Northwestern and University of Lagos	Dry season	July 2017	Multi-stage random sampling	English, Yoruba, Pidgin	Paper (Enketo)	Yes	397
	Kahemba, DRC (1.0)	Oregon Health Sciences University, Michigan State University	NCT03157336: Toxicodietary and genetic determinants of susceptibility to neurodegeneration	Oregon Health Sciences University	Rainy season	July 2017	Cluster randomized control trial	Kikongo, Lingala	Paper/tablet hybrid (ODK)	No	229
	Bahir Dar, Ethiopia (1.0)	Oregon State University	The Women's Development Army in rural Ethiopia: discourses and experiences of health worker status, motivation, and well-being	Oregon State University, reliant on Northwestern	Rainy season	July 2017	Stratified random	Amharic	Tablet (KoboToolbox)	No	259
	Singida, Tanzania (1.0)	Cornell University, Northwestern University	NCT02761876: Singida Nutrition and Agroecology Project	Cornell University	Dry season	July 2017	Purposive, community led	Swahili	Tablet (ODK)	No	100 6
	Lilongwe, Malawi (1.0)	Georgia State University	Standalone	Georgia State University	Neither rainy nor dry season	July 2017	Cluster random	Chichewa, English	Tablets (ODK)	No	303
	Arua, Uganda (1.0)	Michigan State University	Standalone	Michigan State reliant on Northwestern	Rainy season	August 2017	Cluster random	Lugbara, English	Paper (Enketo)	No	260
	Seme, Kenya (1.0)	Pamoja Community	Standalone	Northwestern, African	Neither rainy nor	August 2017	Simple random	Luo, Swahili,	Tablet (ODK)	Yes	247

For peer review only - http://bmjopen.bmj.com/site/about/guidelines.xhtml

		Based Organization, Northwestern University		Medical Research Foundation (AMREF)	dry season			English			
	Kampala, Uganda (1.0)	T-Group Kampala, University of Amsterdam	Standalone	Northwestern and T-Group Kampala	Dry season	August 2017	Simple random	Luganda, English	Paper (Enketo)	No	246
	Morogoro, Tanzania (3.0)	Workman Consulting, Northwestern University	Standalone	Northwestern and Sokoine University of Agriculture	Dry season	March 2018	Cluster random	Swahili	Paper (Enketo)	No	*
	Kisoro, Uganda (3.0)	University of the West of England	Standalone	Exempt	Rainy season	April 2018	TBD	TBD	Tablet (ODK)	No	*
East Asia & Pacific	Upolu, Samoa (1.0)	Yale University	Development of a group prenatal care intervention to address maternal and child NCD risk in American Samoa	Yale University	*	April 2018	Randomized controlled design (intervention/control)	Samoan	Paper (Excel)	No	*
	Labuan Bajo, Indonesia (3.0)	University of the West of England	Standalone	Exempt	*	April 2018	**TBD**	Indonesian	Tablet (ODK)	No	*
Europe & Central Asia	Dushanbe, Tajikistan (1.0)	Arizona State University and M-Vector	Global Ethnohydrology Study—ASU	Arizona State	Dry season	July 2017	Communities purposively selected, every 3rd residence sampled for freestanding buildings, every 5th residence within apartment buildings	Tajik, Russian	Tablet (CSPro)	Yes	225
Latin America & the Caribbean	Pecem & Ceará, Brazil (1.0)	Texas A&M University	NSF1560962: Urban water provisioning systems and household water security	Texas A&M	Neither rainy nor dry season	March 2017	Cluster random	Portuguese	Paper (Enketo)	No	223
	Merida, Mexico (1.0)	Michigan State University	Standalone	Michigan State reliant on Northwestern	Dry season	July 2017	Cluster random	Spanish	Paper (Enketo)	No	250

				University							
	Acatenango, Guatemala (1.0)	Arizona State University	Global Ethnohydrology Study—ASU	Arizona State	Rainy season	July 2017	Cluster-random	Spanish	Paper (Excel)	No	150
	Honda, Colombia (1.0)	Pontificia Universidad Javeriana, Northwestern University	Standalone	Northwestern, Pontificia Universidad Javeriana	Dry season	August 2017	Cluster random	Spanish	Tablet (ODK)	No	252
	San Borja, Bolivia (1.0)	Pennsylvania State University	Standalone	Northwestern	Middle/end of dry season	December 2017	Cluster random	Spanish	Paper (Excel)	No	250
	Chiquimula, Guatemala (2.0)	McGill University	Standalone	McGill, Northwestern	Dry season	January 2018	Single-stage systematic sampling	Spanish	Paper (Excel)	No	314
	Gressier, Haiti (2.0)	University of Florida	Standalone	University of Florida	Middle/end of dry season	March 2018	Cluster random	Creole	Paper (Excel)	No	300
Middle East & North Africa	Beirut, Lebanon (2.0)	American University of Beirut	Standalone	Northwestern, American University of Beirut	Rainy season	January 2018	Cluster random		Tablet (ODK)	Yes	474
South Asia	Kathmandu, Nepal (1.0)	Arizona State University and Kathmandu Upatyaka Khanepani Limited (KUKL)	Global Ethnohydrology Study—ASU	Arizona State	Rainy season	June 2017	Cluster random	Nepali	Tablet (ODK)	Yes	263
	Pune, India	Cornell and Northwestern University	NCT0251259: Evaluating the Pharmacokinetics, Tolerability, and Safety of Once-Weekly Rifapentine and Isoniazid in HIV-1-Infected and HIV-1-Uninfected Pregnant and Postpartum	Cornell University	n.d.	April 2018	Parallel assignment, non-randomized	TBD	Paper (Excel)	No	*

For peer review only - http://bmjopen.bmj.com/site/about/guidelines.xhtml

		Women With Latent Tuberculosis Infection									
Hajipur, Pakistan (2.0)	University of Washington	Standalone	Exempt	n.d.	March 2018	Cluster random	Seraikee, Urdu,	Paper (Excel)	No	*
Rajasthan, India (3.0)	Anode Governance Lab	Standalone	Exempt	n.d.	March 2018	Cluster random	Hindi	Paper (Excel)	No	*
Dhaka and Chakaria, Bangladesh (3.0)	International Centre for Diarrhoel Disease Research, Bangladesh (iccdr,b)	Standalone	Exempt	n.d.	April 2018	Cluster random	Bangla	Paper (Excel)	No	*

*information forthcoming, data collection ongoing

0

For peer review only - http://bmjopen.bmj.com/site/about/guidelines.xhtml

Implementation

Training

An HWISE training manual was developed to provide guidance on implementation.[30] This manual outlines preferred sampling strategy, minimum sample size, instructions for collecting data and choosing unique participant identification numbers, and detailed information explaining the rationale and supplementary information for each HWISE item and survey section. This manual has been translated from English into Arabic[31] and was adapted for use in Uganda.[32]

Each site has a formally-appointed lead investigator responsible for all training, sampling, recruitment, and data collection. In each site, 5-10 enumerators with survey implementation experience, knowledge of the area and context, and fluency in the local language(s) are recruited when possible. Enumerators at all sites attend a 1-2-day training. The first portion of the training curriculum is didactic and follows the survey manual. The second half of the training is interactive and tactile, with enumerators piloting the survey with one another and troubleshooting any issues that arise. After the initial training, the site lead and/or study coordinator accompany enumerators during data collection and provide feedback until enumerators are sufficiently comfortable with the survey to administer it with minimal guidance.

Data collection and management

Enumerators conduct interviews with the person who identifies themselves to the enumerator as the most knowledgeable about the household water acquisition and use. In addition to the water insecurity experience items described above, data are collected on sociodemographic characteristics; water acquisition, use and storage; household food insecurity (using the Household Food Insecurity Access Scale[21]); perceived stress (using a modified, 4-item perceived stress scale[33]); and data quality (Supplemental Material 2: HWISE survey 3.0). These additional

For peer review only - http://bmjopen.bmj.com/site/about/guidelines.xhtml

data are used to help validate the scale and to explore other water insecurity phenomena in a cross-cultural manner.[34] Each interview lasts approximately 45 minutes, and we expect data collection to last approximately 10-14 days in each standalone survey site (Table 3).

Implementation of HWISE data collection began in March 2017 and is expected to end in mid-2018. Data are collected using both paper and tablet-based collection platforms: Open Data Kit (ODK), opendatakit.org;[35] CSPro, csprousers.org; KOBO Toolbox (Cambridge, MA), kobotoolbox.org.

To reduce data collection errors, tablet-based platforms are programmed to include permissible ranges of responses, skips for questions that are not applicable, and survey items in the language(s) most common to each study site. Most responses from paper surveys are entered by enumerators, study coordinators, data managers, and/or site PIs into an online data collection platform (Enketo, enketo.org). Microsoft Excel is used when internet access is unreliable. A further strategy for ensuring data quality is that all surveys contain a concluding module on perceived data quality (e.g., explanation of missing data, distractions, issues with recruitment) that is filled in by the enumerator immediately post-interview.

Data are uploaded to a secure centralized aggregate server (Google App Engine). Stata 14 (StataCorp, College Station, TX, USA) is used for data cleaning following a data cleaning protocol agreed upon by the HWISE Consortium (Supplemental Material 3: Data cleaning protocol).

Analytic strategy

Three software packages will be used for analyses: Stata14 (StataCorp, College Station, TX, USA) will be used to clean the data and run basic descritpives. Mplus version 8 (MPLUS,

For peer review only - http://bmjopen.bmj.com/site/about/guidelines.xhtml

Los Angeles, CA, USA) will be used for Classical Test Theory analysis. WINSTEPS (Winsteps®, Beaverton, OR, USA) will be used for Item Response Theory modeling.

Content and face validity

Content validity (i.e., if items adequately measure the domain of interest) is part of Item development (Table 1). It is assessed in the first eight sites through cognitive interviews with 12 purposively-selected individuals (Table 3). Participants are asked to "think aloud" or "tell [the enumerator] about" their understanding of each of the HWISE items as they complete the pilot survey, and the interviewer records any issues and probes in detail on each as they complete the task.[36] This validation builds on Delphi methodology conducted with international experts from academia, policy, and programmatic disciplines across the fields of food and water insecurity for the earlier, Kenyan-specific scale.[8]

Face validity is assessed at each site (Table 1). First, the survey is translated from English into the language(s) of implementation and then back-translated. Then, enumerators, the predominance of whom are recruited from the target population, pre-test surveys with one another to ensure that questions are appropriate to the setting, the concept of water insecurity is understood, and translations are consistent with local dialects.

Face validity is further ensured when site leads debrief enumerators after each day of data collection and record all the details as project field notes. The debrief is centered on experiences in the community, survey questions that are difficult to administer, and any other problems encountered. At the end of data collection, enumerators engage in a final debrief, and in some cases, use a semi-structured survey that pulls the same information from across the entire site. Site leads are also orally interviewed at the end of study activities by members of the HWISE Consortium about their experiences with project implementation, perceptions of questions by

For peer review only - http://bmjopen.bmj.com/site/about/guidelines.xhtml

enumerators and participants, and any additional topics that should be included or excluded in the final scale. These debriefing interviews with site leads provide additional feedback to iteratively improve training and item refinement.

Phase 2: Scale development

Scale development and evaluation will be informed by two theoretical approaches to scale analysis: Classical Test Theory[37] implemented by factor analysis and Item Response Theory[38] using Rasch models.

Item reduction

First, items with large cumulative missing cases (>30%) i.e. "don't know," "non-applicable," or true missing responses will be dropped (Table 1). This will help to eliminate items that are not understood or do not reflect cross-cultural experiences of water insecurity because they are not widely applicable.

From there, items will be further dropped based on low correlation coefficients. In Classical Test Theory, we will identify items with low (<0.30) inter-item correlation coefficients and very low (<0.30) item-total correlation coefficients across the multiple sites in this study.[39,40]

Within the Rasch paradigm, we will identify and remove items that misfit the models by assessing infit and outfit.[41,42] Condition item independence (i.e., items conditional on the scale that are not correlated) will be assessed using residual correlation metrics. Items will be dropped if residual correlation is >0.2.[43]

Identify factor structure

Factor analysis with data from multiple sites will be used to identify the optimal latent structure. We will examine this structure site-by-site, comparing factor structures, magnitudes of factor loadings, eigenvalues for sample correlation matrices, and global model fitness statistics.

For peer review only - http://bmjopen.bmj.com/site/about/guidelines.xhtml

Items with very low factor loadings (<0.3), split factor loadings, and high residual variances will be considered for deletion.[40]

Assess Measurement Equivalence

Measurement equivalence concerns the extent to which the psychometric properties of the observed indicators are generalizable across groups or over time.[44–47] It holds "when a test measures a construct in the same way regardless of group membership and is violated when individuals from different groups respond to the test in a dissimilar manner".[27] A violation of equivalence implies our inability to make comparisons about scale values across groups (e.g. sites, cultures, languages).[27] To determine measurement equivalence across sites in HWISE 1.0, 2.0, and 3.0, we will conduct confirmatory factor analysis using multi-group confirmatory factor analysis and alignment optimization.

We will also create a map of item severity to display how the items are arranged from most severe to least severe to assess our latent trait, water insecurity. Items will be normalized with mean of zero and a standard deviation of one for each site. The severity of items will be compared across sites and then matched for each site.[43]

Phase 3: Scale evaluation

Reliability

Once a water insecurity scale that is equivalent across sites is provisionally identified, we will use scale scores in both weighted forms (factor scores) and unweighted forms (sum scores) to assess the external validity of our scale.

To test for the internal consistency of the items, we will estimate Cronbach's alpha for the aggregated data and for each site.[48] The Rasch reliability statistic is equivalent to Cronbach's alpha. In our analyses, we will consider a separation reliability to be ideal if it is greater than 0.90.[43]

For peer review only - http://bmjopen.bmj.com/site/about/guidelines.xhtml

Validity

We will then examine 3 types of validity: predictive, convergent, and discriminant validity. Predictive validity is “the extent to which a measure predicts the answers to some other question or a result which it ought to be related with”.[40,49] Using structural equation models, we will test for predictive validity by regressing scores of food insecurity, perceived stress, water sharing practices, and income on HWISE scores.

Convergent validity is the “degree to which scores on a studied instrument are related to measures of other constructs that can be expected on theoretical grounds and accumulated knowledge to be close to the one tapped into by this instrument”.[39,40] To test for convergent validity, we will assess the relationship between HWISE scale scores and individual items that have shown to be closely related to the concept of water insecurity. Specifically, we will use linear regression to examine the strength of the relationship between the sum scores of HWISE scale and time to water source, number of trips to water source, amount of stored drinking water, and amount of money spent purchasing water. Larger regression coefficients and smaller standard deviations of residuals will be indicative of support for convergent validity.

Discriminant validity is the “degree to which scores on a studied instrument are differentiated from behavioral manifestations of other constructs”.[39,40] Using linear regression, we will examine the strength of the relationship between the water insecurity scale scores and total amount of water stored (in liters). Non-significant regression coefficients or very weak associations will be indicative of support for discriminant validity.

A test of differentiation between a range of “known groups” will be conducted using the Student’s t-test and analysis of variance;[39,40] these groups will be based on theoretical evidence and accumulated knowledge. We will determine the distribution of household water insecurity

For peer review only - http://bmjopen.bmj.com/site/about/guidelines.xhtml

scores across known groups, including primary source of drinking water, water treatment, perception of water safety, socio-economic status, gender of household head, and water shortages across months of the year. Under the Rasch measurement model, differentiating between known groups will also be conducted using differential item functioning (DIF). We will determine whether each scale item performs differently in each of the subgroups. DIF is attained when the probabilities of an item being endorsed is unequal for the two subgroups.[40,50]

Selection of the set of items to be included in the final scale will be based on several criteria. The criteria for inclusion of an item are: reliable in each site, fits theoretically and empirically with an important sub-domain, has face and content validity in each site, reflects severity in an equivalent manner across sites, and contributes to predictive, convergent, and discriminant validity in each site.[51] We anticipate that not every item will meet each criterion perfectly, and judgment about tradeoffs of which items to include will be required. These judgments will be made considering the additional criteria of having the set of items in the scale cover as many sub-domains as reasonably possible.

Mid-study evaluations

In addition to these endpoint analyses, we will assess the HWISE items at regular intervals in the study to reduce items and gain preliminary insights into factor structure. We anticipate three such mid-study evaluations; each subsequent resultant version of the module/survey will be numbered sequentially, e.g. HWISE 1.0, HWISE 2.0, and HWISE 3.0 (Supplemental Material 1).

In August 2017, five months after data collection for HWISE 1.0 began in 17 sites, HWISE Consortium members met at Northwestern University to review data received to date

For peer review only - http://bmjopen.bmj.com/site/about/guidelines.xhtml

and thematically sort and rank HWISE items. This led to the reduction and refinement of HWISE for the second round of survey implementation (HWISE 2.0), which was administered across four sites. In February 2018, HWISE Consortium members involved in scale validation met at McGill University to review HWISE 2.0 responses to date and further refine the HWISE module. HWISE 3.0 survey implementation is ongoing in further sites, and data collection is anticipated to conclude in mid-2018.

Ethics

All participants will be verbally consented by enumerators in their language of choice using a standardized script (Supplemental Material 2). Study activities are reviewed and approved by ethical review boards at Northwestern University, African Medical Research Foundation (AMREF), American University at Beirut, Arizona State University, Cornell University, Delaware State University, Florida State University, Georgia State University, Ghana Water Company, International Centre for Diarrhoeal Disease Research, Bangladesh (icddr,b), Johns Hopkins University, College of Medicine at the University of Lagos, University of Miami, McGill University, Michigan State University, Nepal Health Research Council, Oregon Health Sciences University, Oregon State University, Penn State University, Pontificia Universidad Javeriana, Sokoine University of Agriculture, Texas A&M University, T-Group Kampala, and Yale University (Table 3).

The final HWISE scale will be presented at scientific, programmatic, and policy venues. The survey will be made available through an open access creative commons license. Data will be available upon request from the HWISE Consortium. Associated findings will be disseminated to scientists, practitioners, and policy makers through peer-reviewed journals and conference presentations. Measures to quantify household food insecurity have been transformative for

For peer review only - http://bmjopen.bmj.com/site/about/guidelines.xhtml

For peer review only

policy, research, and humanitarian aid efforts globally, and we expect that an analogous measure for water insecurity will achieve similar impacts.

Acknowledgements
We are very grateful to our participants, without whom this scale would not be possible. We would also like to thank the site PIs and field teams for their ongoing hard work and dedication to this project. Site PIs are as follows: Ashley Hagaman (Kathmandu, Nepal); Justin Stoler and Raymond Tutu (Accra, Ghana); Monet Niesluchowski (Dushanbe, Tajikistan); Wendy Jepson, Carol Lima, Marcelo Soares, and Jader Santos (Pecem and Ceara, Brazil); Amber Pearson (Merida, Mexico and Arua, Uganda); Jonathan Maupin (Acatenango, Guatemala); Asher Rosinger (San Borja, Bolivia); Hugo Melgar-Quiñonez and Luisa Samayoa Figueroa (Chiquimula, Guatemala); Kelly Chapman (Gressier, Haiti); Mobolanle Balogun (Lagos, Nigeria); Desiré Tshala-Katumbay (Kahemba, Democratic Republic of Congo); Patrick Mbullo (Seme, Kenya); Kenneth Maes and Yihenew Tesfaye (Bahir Dar, Ethiopia); Marriane V. Santoso (Singida, Tanzania); Alex Trowell (Kampala, Uganda); Ellis Adams (Lilongwe, Malawi); Chad Staddon (Kisoro, Uganda); Cassandra Workman (Morogoro, Tanzania); Farooq (Jam) Ahmed (Hajipur, Pakistan); Sonali Srivastava (Rajasthan, India); Jyoti Mathad (Pune, India); Sabrina Rasheed (Dhaka and Chakaria, Bangladesh); Stroma Cole and Marta (Ica) Muslin (Labuan Bajo, Indonesia); Hala Ghattas and Zeina Jamaluddine (Beirut, Lebanon); Nicola Hawley (Upolu, American Samoa)

Funding
We would like to acknowledge our funders: the Competitive Research Grants to Develop Innovative Methods and Metrics for Agriculture and Nutrition Actions (IMMANA Grants). IMMANA is funded with UK Aid from the UK government. This project was also supported by the Buffett Institute for Global Studies and the Center for Water Research at Northwestern University; Arizona State University's Center for Global Health at the School of Human Evolution and Social Change and Decision Center for a Desert City (National Science Foundation SES-1462086); the Office of the Vice Provost for Research of the University of Miami. Sera Young was supported by the National Institutes of Health (NIMH R21 MH108444; NIMH K01 MH098902) Wendy Jepson was supported by the National Science Foundation (BCS-1560962).

Author contributions
SLY conceptualized the study, developed HWISE items, wrote the manuscript, obtained funding, and oversaw data collection and analysis. SMC helped develop HWISE items, wrote the manuscript, and managed data. GB developed the data analysis and validation plan, and helped write the analytic section of the manuscript. TBN assisted with study design, and supported scale analysis and validation. ZJ proposed data analysis, and helped write the analytic section of the manuscript. JDM developed tools for data collection and managed data. AB proposed analyses for item development. EF and HMQ proposed data analysis. WJ developed HWISE items. RCS supported development of HWISE items and assisted with preparation of the manual. JS proposed analyses for item development. AW developed HWISE items and proposed analyses for item development. All authors critically reviewed and approved the final draft of the manuscript.

For peer review only - http://bmjopen.bmj.com/site/about/guidelines.xhtml

Conflicts of interest
None declared

Data Statement
Data are currently being collected and are not yet available for access.

References
1. Jepson WE, Wutich A, Collins SM, Boateng GO, Young SL. Progress in household water insecurity metrics: a cross-disciplinary approach. WIREs Water. 2017 Apr 11;4(3):e1214-21.

2. Geere J-AL, Hunter PR, Jagals P. Domestic water carrying and its implications for health: a review and mixed methods pilot study in Limpopo Province, South Africa. Environmental Health. 2010 Jan 1;9(1):52–13.

3. Prüss-Ustün A, Bartram J, Clasen T, Colford JM, Cumming O, Curtis V, et al. Burden of disease from inadequate water, sanitation and hygiene in low- and middle-income settings: a retrospective analysis of data from 145 countries. Tropical Medicine & International Health. 2014 Aug;19(8):894–905.

4. Sorenson SB, Morssink C, Campos PA. Safe access to safe water in low income countries: Water fetching in current times. Social Science & Medicine. 2011 May;72(9):1522–6.

5. Mason LR. Examining Relationships between Household Resources and Water Security in an Urban Philippine Community. Journal of the Society for Social Work and Research. 2014 Dec 1;5(4):489–512.

6. Wutich A, Brewis A. Food, Water, and Scarcity: Toward a Broader Anthropology of Resource Insecurity. Current Anthropology. 2014 Aug;55(4):444–68.

7. Cook C, Bakker K. Water security: debating an emerging paradigm. Global Environmental Change. 2012;22(1):94–102.

8. Boateng G, Collins SM, Mbullo P, Wekesa P, Onono M, Neilands TB, et al. A Novel Household Water Insecurity Scale: Procedures and Psychometric Analysis Among Postpartum Women in Western Kenya. bioRxiv. doi: 10.1101/294298.

9. Workman CL, Ureksoy H. Water insecurity in a syndemic context: Understanding the psycho-emotional stress of water insecurity in Lesotho, Africa. Social Science & Medicine. 2017 Apr;179:52–60.

10. Weiser SD, Young SL, Cohen CR, Kushel MB, Tsai AC, Tien PC, et al. Conceptual framework for understanding the bidirectional links between food insecurity and HIV/AIDS. American Journal of Clinical Nutrition. 2011 Dec;94(6):1729S-1739S.

11. Mekonnen MM, Hoekstra AY. Four billion people facing severe water scarcity. Science Advances. 2016 Feb 12;2(2):e1500323–e1500323.

For peer review only - http://bmjopen.bmj.com/site/about/guidelines.xhtml

12. Falkenmark M, Lundqvist J, Widstrand C. Macro-scale water scarcity requires micro-scale approaches. Aspects of vulnerability in semi-arid development. Nat Resour Forum. 1989 Nov;13(4):258–67.

13. Rijsberman FR. Water scarcity: Fact or fiction? Agricultural Water Management. 2006;80(1–3):5–22.

14. Sullivan CA, Meigh JR, Giacamello, AM. The Water Poverty Index: Development and application at the community scale. Natural Resources Forum. 2003;189–99.

15. Global Water Partnership. Proceedings from the GWP workshop: Assessing Water Security with Appropriate Indicators [Internet]. Stockholm; 2014 p. 120. Available from: http://www.gwp.org/Global/ToolBox/Publications/P763_GWP_Proceedings_Paper.pdf

16. Jepson W. Measuring no-win waterscapes: Experience-based scales and classification approaches to assess household water security in colonias on the US--Mexico border. Geoforum. 2014;51(C):107–20.

17. Wutich A, Ragsdale K. Water insecurity and emotional distress: coping with supply, access, and seasonal variability of water in a Bolivian squatter settlement. Social science & medicine. 2008;67(12):2116–25.

18. Tsai AC, Kakuhikire B, Mushavi R, Vořechovská D, Perkins JM, McDonough AQ, et al. Population-based study of intra-household gender differences in water insecurity: reliability and validity of a survey instrument for use in rural Uganda. Journal of Water and Health. 2015; 14(2): 280-292.

19. Stevenson EGJ, Greene LE, Maes KC, Ambelu A, Tesfaye YA, Rheingans R, et al. Water insecurity in 3 dimensions: an anthropological perspective on water and women's psychosocial distress in Ethiopia. Social Science & Medicine. 2012;75(2):392–400.

20. Pérez-Escamilla R, Melgar-Quiñonez H, Nord M, Alvarez Uribe MC, Segall-Correa A. Escala Latinoamericana y Caribeña de Seguridad Alimentaria (ELCSA) [Latinamerican and Caribbean Food Security Scale]. Perspectivas en Nutrición Humana. 2007;117–34.

21. Coates J, Swindale A, Bilinsky P. Household food insecurity access scale (HFIAS) for measurement of food access: Indicator guide. Washington DC: Food and Nutrition Technical Assistance Project; 2007.

22. Ballard T, Kepple AW, Cafiero C. The Food Insecurity Experience Scale: development of a global standard for monitoring hunger worldwide. Technical Paper. Rome, Italy: Food & Agriculture Organization; 2013. Available from: http://www.fao.org/economic/ess/ess-fs/voices/en.

23. Haynes SN, Richard DC, Kubany ES. Content Validity in Psychological Assessment: A Functional Approach to Concepts and Methods. Psychological Assessment. 1995;7(3):238–47. doi: http://dx.doi.org/10.1037/1040-3590.7.3.238.

For peer review only - http://bmjopen.bmj.com/site/about/guidelines.xhtml

24. Krumdieck N, Collins S, Wekesa P, Mbullo P, Boateng G, Onono M, et al. Household water insecurity is associated with a range of negative consequences among pregnant Kenyan women of mixed HIV status. Journal of Water and Health. 2016; 14(6):1028-1031. doi:10.2166/wh.2016.079.

25. McCoach DB, Gable RK, Madura JP. Defining, Measuring, and Scaling Affective Constructs. In: Instrument Development in the Affective Domain [Internet]. New York, NY: Springer New York; 2013 [cited 2018 Mar 23]. p. 33–90. Available from: http://link.springer.com/10.1007/978-1-4614-7135-6_2

26. Webb P, Iskandarani M. Water insecurity and the poor: issues and research needs. 1998 [cited 2016 Apr 20]; Available from: http://www.iatp.org/files/Water_Insecurity_and_the_Poor_Issues_and_Resea.htm

27. Kim ES, Cao C, Wang Y, Nguyen DT. Measurement Invariance Testing with Many Groups: A Comparison of Five Approaches. Structural Equation Modeling: A Multidisciplinary Journal. 2017 Jul 4;24(4):524–44.

28. Guadagnoli E, Velicer WF. Relation of sample size to the stability of component patterns. Psychological Bulletin. 1988 Mar;103(2):265–75.

29. Turner AG. Handbook on designing of household sample surveys. Geneva, Switzerland: United Nations Statistics Division; 2003.

30. Collins SM, Young SL. The Household Water InSecurity Experiences (HWISE) Scale Study Manual. 2018. doi: 10.21985/N2M10F.

31. Jamaluddine Z, Khabsa J, Ghattas H. The Household Water InSecurity Experiences (HWISE) Scale Manual (Arabic). 2017. doi:10.21985/N2FX17.

32. Asiki G, Pearson A. The Household Water InSecurity Experiences (HWISE) Scale Study Manual (Uganda). 2017. doi: 10.21985/N25D7V.

33. Cohen S, Kamarck T, Mermelstein R. A global measure of perceived stress. J Health Soc Behav. 1983;24(4):385–96.

34. Brewis A, Rosinger A, Wutich A, Adams E, Cronk L, Pearson A, et al. Household Water Sharing Favors Charitable Transfers to Neighbors: Evidence from Eight Water-Insecure Sub-Saharan African Sites. Economic Anthropology. Under review.

35. Brunette W, Sundt M, Dell N, Chaudhri R, Breit N, Borriello G. Open data kit 2.0: expanding and refining information services for developing regions. In: Proceedings of the 14th Workshop on Mobile Computing Systems and Applications. ACM; 2013. p. 10.

36. Ericsson KA, Simon HA. Verbal Reports as Data. Psychological Review. 1980;87(3):215–51.

For peer review only

For peer review only - http://bmjopen.bmj.com/site/about/guidelines.xhtml

37. Cappelleri JC, Jason Lundy J, Hays RD. Overview of Classical Test Theory and Item Response Theory for the Quantitative Assessment of Items in Developing Patient-Reported Outcomes Measures. Clinical Therapeutics. 2014 May;36(5):648–62.

38. Harvey RJ, Hammer AL. Item Response Theory. The Counseling Psychologist. 1999 May;27(3):353–83.

39. Raykov T, Marcoulides GA. Introduction to psychometric theory. New York, NY: Routledge; 2011.

40. Boateng GO, Neilands TB, Frongillo EA, Melgar-Quiñonez H, Young SL. Best Practices for Developing and Validating Scales for Public Health and Nutrition Research: A Primer. Frontiers in Public Health. Under review;

41. Wright BD, Stone M. Best Test Design: Rasch Measurement. Chicago, IL: Mesa Press; 1979.

42. Smith EV. Evidence for the reliability of measures and validity of measure interpretation: a Rasch measurement perspective. J Appl Meas. 2001;2(3):281–311.

43. Linacre JM, Wright BD. A user's guide to BIGSTEPS: Rasch-model computer program. Chicago: Mesa Press; 1993.

44. Byrne B, Watkins D. The issue of measurement invariance revisited. Journal of cross-cultural psychology. 2003;34(2):155–75.

45. Harkness JA, Vijver FJR van de, Mohler PP, editors. Cross-cultural survey methods. Hoboken, N.J: J. Wiley; 2003. 420 p. (Wiley series in survey methodology).

46. Leroy JL, Ruel M, Frongillo EA, Harris J, Ballard TJ. Measuring the Food Access Dimension of Food Security: A Critical Review and Mapping of Indicators. Food and Nutrition Bulletin. 2015 Jun;36(2):167–95.

47. Sideridis GD, Tsaousis I, Al-harbi KA. Multi-Population Invariance With Dichotomous Measures: Combining Multi-Group and MIMIC Methodologies in Evaluating the General Aptitude Test in the Arabic Language. Journal of Psychoeducational Assessment. 2015 Sep;33(6):568–84.

48. Cronbach LJ. Coefficient alpha and the internal structure of tests. Psychometrika. 1951 Sep;16(3):297–334.

49. Fowler FJ. Improving survey questions: design and evaluation. Thousand Oaks: Sage Publications; 1995. 191 p. (Applied social research methods series).

50. Angoff W. Use of difficulty and discrimination indices for detecting item bias. In: Handbook of Methods for Detecting Test Bias. Baltimore, MD: Johns Hopkins University Press; 1982. p. 96–116.

For peer review only - http://bmjopen.bmj.com/site/about/guidelines.xhtml

51. Gregorich SE. Do self-report instruments allow meaningful comparisons across diverse population groups? Testing measurement invariance using the confirmatory factor analysis framework. Medical care. 2006;44(11 Suppl 3):S78.

For peer review only

For peer review only - http://bmjopen.bmj.com/site/about/guidelines.xhtml

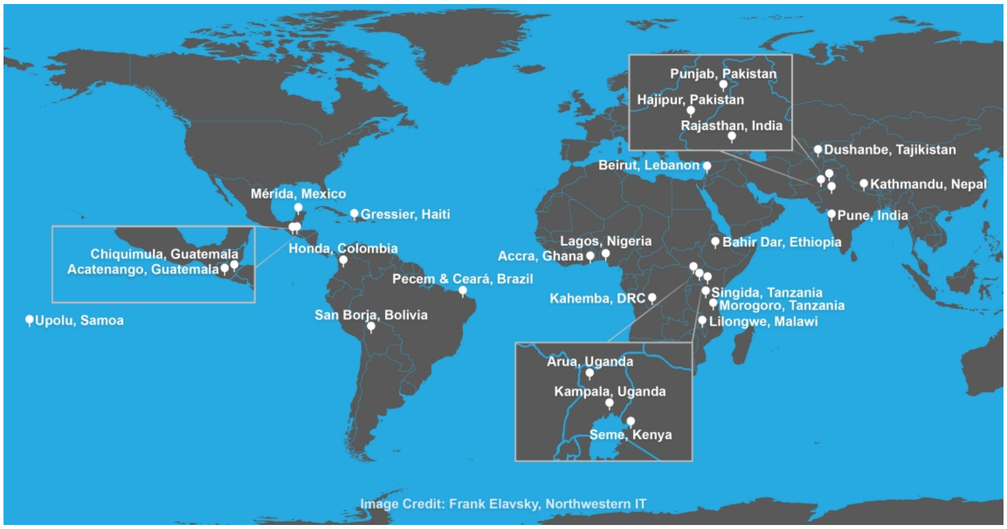

Figure 1. Map of HWISE study sites

1322x687mm (72 x 72 DPI)

Supplemental Table 1. Overview of HWISE survey items, by implementation phase

<table>
<tr><th></th><th>HWISE 1.0</th><th>HWISE 2.0</th><th>HWISE 3.0</th></tr>
<tr><td>Response Options:</td><td>Never (0 times), Rarely (1-2 times), Sometimes (3-10t times), Often (11-20 times), Always (more than 20 times)</td><td>Never (0 times), Rarely (1-2 times), Sometimes (3-10t times), Often (11-20 times), Always (more than 20 times)</td><td>Never (0 times), Rarely (1-2 times), Sometimes (3-10t times), Often (11-20 times), Always (more than 20 times)</td></tr>
<tr><td>Number of Questions:</td><td>32</td><td>29</td><td>30</td></tr>
<tr><td>Dimensions:</td><td>Multidimensional: psychological, disease, nutrition, economic, social</td><td>Multidimensional: psychological, disease, nutrition, economic, social, cultural</td><td>Multidimensional: psychological, disease, nutrition, economic, social, cultural</td></tr>
<tr><th>Domains</th><th>HWISE 1.0</th><th>HWISE 2.0</th><th>HWISE 3.0</th></tr>
<tr><td rowspan="5">PSYCHOLOGICAL</td><td>In the last 4 weeks, how frequently did you or anyone in your household worry you would not have enough water for all of your household needs?</td><td colspan="2">In the last 4 weeks, how frequently did you or anyone in your household worry you would not have enough water for all of your household needs?</td></tr>
<tr><td>In the last 4 weeks, how frequently have you or anyone in your household worried about the safety of the person getting water for your household?</td><td colspan="2">In the last 4 weeks, how frequently have you or anyone in your household worried about the safety of the person getting water for your household?</td></tr>
<tr><td>In the last 4 weeks, how frequently did you or anyone in your household feel upset about your water situation?</td><td colspan="2">In the last 4 weeks, how frequently did you or anyone in your household feel angry about your water situation?</td></tr>
<tr><td>In the last 4 weeks, how frequently has your or anyone in your household's day been interrupted by your water situation, including getting or distributing water within the household?</td><td colspan="2">In the last 4 weeks, how frequently has you or anyone in your household had to change schedules/plans due to problems with your water situation, such as problems getting or distributing water within the household? Activities that may have been interrupted include caring for others, doing household chores, etc.</td></tr>
<tr><td>–</td><td colspan="2">How satisfied are you with your water situation on a scale of 1-5? (1 is not at all satisfied and 5 is completely satisfied).</td></tr>
<tr><td rowspan="6">SOCIAL</td><td>In the last 4 weeks, how frequently have problems with water prevented you or anyone in your household from attending social events (i.e. church, funerals, community gatherings, etc.)?</td><td colspan="2">In the last 4 weeks, how frequently have problems with water prevented you or anyone in your household from attending social or cultural events (e.g. church, funerals, community gatherings, cultural practices, etc.)?</td></tr>
<tr><td>In the last 4 weeks, how frequently did the children in your household miss school because they were getting water?</td><td colspan="2">In the last 4 weeks, how frequently did the children in your household miss school or go to school late because of problems with water (e.g. time spent fetching water, lack of water for bathing, etc.)?</td></tr>
<tr><td>In the last 4 weeks, how frequently did you or anyone in your household have problems with water that caused difficulties with neighbors or others in the community?</td><td colspan="2">In the last 4 weeks, how frequently did you or anyone in your household have problems with water that caused difficulties with neighbors, water providers, or others in the community?</td></tr>
<tr><td>In the last 4 weeks, how frequently did you or anyone in your household have problems with water that caused difficulties within your household?</td><td colspan="2">In the last 4 weeks, how frequently did you or anyone in your household have problems with water that caused difficulties within your household?</td></tr>
<tr><td>–</td><td colspan="2">In the last 4 weeks, how frequently have you or anyone in your household been unable to access the water that you preferred?</td></tr>
<tr><td>–</td><td colspan="2">In the last 4 weeks, how frequently have problems with water caused you or anyone in your household to feel ashamed/excluded/stigmatized?</td></tr>
<tr><th>Domains</th><th>HWISE 1.0</th><th>HWISE 2.0</th><th>HWISE 3.0</th></tr>
<tr><td rowspan="6">ECONOMIC</td><td>In the last 4 weeks, how frequently has the time spent getting water prevented you or anyone in your household from earning money (e.g. engaging in paid work, economic activities)?</td><td colspan="2">In the last 4 weeks, how frequently have problems with water prevented you or anyone in your household from earning money (e.g. engaging in paid work, economic activities)?</td></tr>
<tr><td>In the last 4 weeks, how frequently have you or anyone in your household lacked money needed to buy water?</td><td colspan="2">In the last 4 weeks, how frequently have you or anyone in your household lacked money needed to buy water?</td></tr>
<tr><td>In the last 4 weeks, how frequently have you or anyone in your household thought of leaving [name of town] because there was no water there?</td><td colspan="2">In the last 4 weeks, how frequently have you or anyone in your household thought of moving dwellings because of the water situation there?</td></tr>
<tr><td>In the last 4 weeks, how frequently did you or anyone in your household want to buy water but there was nowhere to buy it from?</td><td colspan="2">In the last 4 weeks, how frequently did you or anyone in your household want to buy water but there was nowhere to buy it from?</td></tr>
<tr><td>In the last 4 weeks, how frequently have you or anyone in your household asked to borrow water from other people?</td><td colspan="2">In the last 4 weeks, how frequently have you or anyone in your household asked to borrow water from other people?</td></tr>
<tr><td>In the last 4 weeks, how frequently have you or anyone in your household loaned water to anyone?</td><td>–</td><td>In the last 4 weeks, how frequently have you or anyone in your household loaned water to anyone?</td></tr>
<tr><td rowspan="12">LIVELIHOODS/HEALTH</td><td>In the last 4 weeks, how frequently has the time spent getting water prevented you or anyone in your household from caring for children in the household?</td><td>–</td><td>–</td></tr>
<tr><td>In the last 4 weeks, how frequently has the time spent getting water prevented you or anyone in your household from doing household chores (such as cooking, preparing food, washing clothes, etc.)?</td><td>–</td><td>–</td></tr>
<tr><td>In the last 4 weeks, how frequently has there not been enough water in the household to wash clothes?</td><td colspan="2">In the last 4 weeks, how frequently has there not been enough water in the household to wash clothes?</td></tr>
<tr><td>In the last 4 weeks, how frequently have you or anyone in your household had to go without washing hands after dirty activities (e.g., defecating or changing diapers, cleaning animal dung) because you didn't have enough water?</td><td colspan="2">In the last 4 weeks, how frequently have you or anyone in your household had to go without washing hands after dirty activities (e.g., defecating or changing diapers, cleaning animal dung) because of problems with water?</td></tr>
<tr><td>In the last 4 weeks, how frequently have you or anyone in your household not had enough water to wash the faces and hands of children in your household?</td><td colspan="2">In the last 4 weeks, how frequently have you or anyone in your household not washed the faces and hands of children because of problems with water?</td></tr>
<tr><td>In the last 4 weeks, how frequently have you or anyone in your household had to go without washing their body because there wasn't enough water?</td><td colspan="2">In the last 4 weeks, how frequently have you or anyone in your household had to go without washing their body because of problems with water (e.g. not enough water, dirty, unsafe)?</td></tr>
<tr><td>In the last 4 weeks, how frequently did you or anyone in your household want to treat your water, but couldn't? By treat, I mean boiling, using chemicals to treat, or other ways you make your water safe to use or drink.</td><td>–</td><td>–</td></tr>
<tr><td>In the last 4 weeks, how frequently have you or anyone in your household drank water that tasted bad?</td><td colspan="2">In the last 4 weeks, how frequently have you or anyone in your household drank water that looked, tasted, and/or smelled bad?</td></tr>
<tr><td>In the last 4 weeks, how frequently have you or anyone in your household actually drank water that you thought was unsafe?</td><td colspan="2">In the last 4 weeks, how frequently have you or anyone in your household drank water that you thought was unsafe?</td></tr>
<tr><td>In the last 4 weeks, how frequently have you or anyone in your household not had enough water to take medications?</td><td>–</td><td>–</td></tr>
<tr><td>In the last 4 weeks, how frequently have you or anyone in your household not gotten water where you wanted to because you were too sick or weak to get water?</td><td>–</td><td>–</td></tr>
<tr><th>Domains</th><th>HWISE 1.0</th><th>HWISE 2.0</th><th>HWISE 3.0</th></tr>
<tr><td rowspan="4">NUTRITION</td><td>In the last 4 weeks, how frequently have you or anyone in your household had to change what was being eaten because there wasn't enough water (e.g. for washing foods, cooking, etc.)?</td><td colspan="2">In the last 4 weeks, how frequently have you or anyone in your household had to change what was being eaten because there were problems with water (e.g. for washing foods, cooking, etc.)?</td></tr>
<tr><td>In the last 4 weeks, how frequently has there not been as much water to drink as you would like for you or anyone in your household?</td><td colspan="2">In the last 4 weeks, how frequently has there not been as much water to drink as you would like for you or anyone in your household?</td></tr>
<tr><td>In the last 4 weeks, how frequently have you or anyone in your household gone to sleep thirsty?</td><td colspan="2">In the last 4 weeks, how frequently have you or anyone in your household gone to sleep thirsty because there wasn't any water to drink?</td></tr>
<tr><td>In the last 4 weeks, how frequently has there been no water whatsoever in your household?</td><td colspan="2">In the last 4 weeks, how frequently has there been no useable or drinkable water whatsoever in your household?</td></tr>
<tr><td rowspan="2">AGRICULTURE</td><td>In the last 4 weeks, how frequently has your household not had enough water for your garden, crops, or fruit trees?</td><td colspan="2">In the last 4 weeks, how frequently has your household water situation impacted the cultivation of your garden, crops, or fruit trees?</td></tr>
<tr><td>In the last 4 weeks, how frequently has your household not had enough water to give to your animals and poultry?</td><td colspan="2">In the last 4 weeks, how frequently has your household water situation impacted your raising of animals and poultry?</td></tr>
<tr><td>OTHER</td><td>In the last 4 weeks, how frequently has your household water supply from your main water source been interrupted?</td><td colspan="2">In the last 4 weeks, how frequently has your household water supply from your main water source been interrupted or limited (e.g. water pressure, less water than expected)?</td></tr>
</table>

HWISE 3.0 Consent Script, Survey, and Sources

Household Water Insecurity Experiences

Version 24, March 6, 2018

For peer review only - http://bmjopen.bmj.com/site/about/guidelines.xhtml

Household Water Insecurity Experiences

HWISE Consent Script

You are being asked to take part in a research study about the different factors affecting water insecurity and health for people in your area. We are asking you to take part because you live in this area and have unique insight into how water is acquired and used in your community.

What this study is about: The purpose of this study is to learn about the various ways people acquire and use water and any consequences that arise from household water insecurity. From this study, we will be able to develop a scale to measure household water insecurity in many settings across the world.
What we will ask you to do: If you agree to be in this study, we will ask you to participate in a brief survey during which we will ask you questions about the composition of your household, water acquisition and use, food insecurity, and stress.

Risks and benefits: There is the risk that you may find some of the questions about your household to be sensitive. There are no immediate benefits to you, but the information learned in this study will be useful for researchers who are studying household water insecurity. The information we learn through this survey may be shared with researchers at other institutions, but your personal identifying information will not be shared or linked to your responses.

Compensation. There is no compensation for taking part in this study.

Your answers will be confidential. We will write or record your responses to this survey using tablets, your name and information will not be included in any part of the survey. The written records and any information you share will be kept private. In any sort of report we make public we will not include any information that will make it possible to identify you. Research records will be kept in a locked file or in secure, password-protected online storage; only researchers will have access to the records.

Taking part is voluntary. Taking part in this study is completely voluntary. You may skip any questions that you do not want to answer. If you decide not to take part or to skip some of the questions, it will not affect your future relationship with Arizona State University or Northwestern University. If you decide to take part, you are free to withdraw at any time.

If you have questions: The lead researcher conducting this study is Dr. Sera Young at Northwestern University. If you have questions later, you can contact Dr. Young at sera.young@northwestern.edu. If you have any questions or concerns regarding your rights as a subject in this study, you may contact the Northwestern University Institutional Review Board (IRB) at +1 (847) 467-1723 or http://www.northwestern.edu.
Now I would like to ask if you agree to participate in this study.
Do you agree to participate in this study? YES/NO
Do you agree to allow me to record your responses to questions? YES/NO

Household Water Insecurity Experiences

HWISE 3.0 Survey

Cross-cultural study of household water insecurity	
Interviewer________________________________	Date___________________
Time at start of interview_____________________	Time at end of interview________________
Country ____________________	Region/District___________________
Neighborhood___________________	
Participant ID:_______________	Language of interview: __________________
Participant gender: (0) Male (1) Female	
Participant place of residence: (0) Rural (2) Peri-urban (3) Urban	
Participant ethnicity:	
Participant ID should start with 1st two letters of country, eg. BA, NE, GU, GH…	

1. Screening Questions

Code Name	Questions	Coding Classification
SQ1a	Do you agree to participate in this survey?	0…No<br>1…Yes
SQ2	Are you 16 years of age or older?	0…No<br>1…Yes
SQ3	Would you consider yourself knowledgeable about water acquisition and use within your household?	0…No<br>1…Yes

2. Socio Demographic Questions

Code Name	Questions	Coding Classification
SD1	Role in household: What is your relationship to the head of your household? By household, I mean all people who sleep under the same roof and take food from the same pot.	1….Self<br>2….Spouse/partner<br>3….Adult child<br>4….Other
SD2	What is the gender of household head?	0…Male<br>1…Female
SD9	What is your current relationship status?	1…Single/separated or divorced<br>2…Widowed<br>3…Have a partner who lives separately<br>4…Have a partner who lives with you<br>5…Married
SD3	How old are you?	
SD4	Who is primarily responsible for making sure there is enough water in the house? By	1…Self<br>2…Spouse/partner

	household, I mean all people who sleep under the same roof and take food from the same pot.<br>*If primary responsibility is shared, choose (5) and then also circle the two categories of people who share the responsibility.*	3…Children<br>4…Other family members<br>5…Shared responsibility
SD5	How many children (≤16 years) live in your household? By household, I mean all people who sleep under the same roof and take food from the same pot.	
SD6	How many adults (>16 years) including yourself live in your household? By household, I mean all people who sleep under the same roof and take food from the same pot.	
SD7	What type of housing do you live in?	1….House/condominium (owned)<br>2….House/condominium (rented)<br>3….Apartment (owned)<br>4…..Apartment (rented)<br>5….Farm (own)<br>6…..Farm (lease)<br>7…..Informal settlement/squatter community<br>8……Refugee/internally displaced person camp<br>9…..Other __________
SD8	What is your religion?	1….Christianity<br>2….Judaism<br>3….Islam<br>4…..Hinduism<br>5…..Buddhism<br>6…..Nonreligious<br>7…...Other
SD8a	What is your denomination?	

**3. Household Water Insecurity Experiences Scale (32 questions)**<br>*Now I'm going to ask you about your own personal experiences with water. For each of the items, please indicate how many times within the past 4 weeks or 30 days. Interviewer, please mark the response that best corresponds with the coding classification. For example, if participants says 15 times, you need to mark/circle code 4.*		
**Code name**	**Question**	**Coding Classification**
HWISE1	How **satisfied** are you with your water situation on a scale of 1-5? (1 is not at all satisfied and 5 is completely satisfied).	1…Not at all satisfied<br>2<br>3<br>4<br>5...Completely satisfied
HWISE2	In the last 4 weeks, how frequently have you or anyone in your household been unable to access the **water that you preferred**?	1...Never (0 times in the last 4 weeks)<br>2...Rarely (1–2 times in the last 4 weeks)<br>3...Sometimes (3–10 times in the last 4 weeks)<br>4...Often (11-20 times in the last 4 weeks)<br>5….Always (More than 20 times in the last 4 weeks)<br>99….Don't know<br>88…Not applicable/I don't have this
HWISE3	In the last 4 weeks, how frequently did you or anyone in your household **worry** you would not have enough water for all of your household needs?	1...Never (0 times in the last 4 weeks)<br>2...Rarely (1–2 times in the last 4 weeks)<br>3...Sometimes (3–10 times in the last 4 weeks)<br>4...Often (11-20 times in the last 4 weeks)<br>5….Always (More than 20 times in the last 4 weeks)<br>99….Don't know<br>88…Not applicable/I don't have this
HWISE4	In the last 4 weeks, how frequently have you or anyone in your household **worried about the safety of the person** getting water for your household? *By getting, I mean: traveling to, collecting the water, and returning with the water.*	1...Never (0 times in the last 4 weeks)<br>2...Rarely (1–2 times in the last 4 weeks)<br>3...Sometimes (3–10 times in the last 4 weeks)<br>4...Often (11-20 times in the last 4 weeks)<br>5….Always (More than 20 times in the last 4 weeks)<br>99….Don't know<br>88…Not applicable/I don't have this
HWISE5	In the last 4 weeks, how frequently has your household water supply from your main water source been **interrupted** or **limited** (e.g. water	1...Never (0 times in the last 4 weeks)<br>2...Rarely (1–2 times in the last 4 weeks)<br>3...Sometimes (3–10 times in the last 4 weeks)

	pressure, less water than expected)?	4...Often (11-20 times in the last 4 weeks)<br>5….Always (More than 20 times in the last 4 weeks)<br>99….Don't know<br>88…Not applicable/I don't have this
HWISE5a	If your supply was interrupted or limited, were these **expected** (announced/scheduled) or unexpected?	0…Unexpected<br>1…Announced/Scheduled
HWISE6	In the last 4 weeks, how frequently has your household water situation impacted the cultivation of your **garden, crops, or fruit trees**?	1...Never (0 times in the last 4 weeks)<br>2...Rarely (1–2 times in the last 4 weeks)<br>3...Sometimes (3–10 times in the last 4 weeks)<br>4...Often (11-20 times in the last 4 weeks)<br>5….Always (More than 20 times in the last 4 weeks)<br>99….Don't know<br>88…Not applicable/I don't have this
HWISE7	In the last 4 weeks, how frequently has your household water situation impacted your raising of **animals and poultry**?	1...Never (0 times in the last 4 weeks)<br>2...Rarely (1–2 times in the last 4 weeks)<br>3...Sometimes (3–10 times in the last 4 weeks)<br>4...Often (11-20 times in the last 4 weeks)<br>5….Always (More than 20 times in the last 4 weeks)<br>99….Don't know<br>88…Not applicable/I don't have this
HWISE8	In the last 4 weeks, how frequently have problems with water prevented you or anyone in your household from **earning money** (e.g. engaging in paid work, economic activities)?	1...Never (0 times in the last 4 weeks)<br>2...Rarely (1–2 times in the last 4 weeks)<br>3...Sometimes (3–10 times in the last 4 weeks)<br>4...Often (11-20 times in the last 4 weeks)<br>5….Always (More than 20 times in the last 4 weeks)<br>99….Don't know<br>88…Not applicable/I don't have this
HWISE9	In the last 4 weeks, how frequently have you or anyone in your household **lacked money** needed to **buy water**?	1...Never (0 times in the last 4 weeks)<br>2...Rarely (1–2 times in the last 4 weeks)<br>3...Sometimes (3–10 times in the last 4 weeks)<br>4...Often (11-20 times in the last 4 weeks)

		5….Always (More than 20 times in the last 4 weeks)<br>99….Don't know<br>88…Not applicable/I don't have this
HWISE10	In the last 4 weeks, how frequently did you or anyone in your household want to buy water but there was **nowhere to buy it from**?	1...Never (0 times in the last 4 weeks)<br>2...Rarely (1–2 times in the last 4 weeks)<br>3...Sometimes (3–10 times in the last 4 weeks)<br>4...Often (11-20 times in the last 4 weeks)<br>5….Always (More than 20 times in the last 4 weeks)<br>99….Don't know<br>88…Not applicable/I don't have this
HWISE11	In the last 4 weeks, how frequently did the children in your household **miss school** or **go to school late** because of problems with water (e.g. time spent fetching water, lack of water for bathing, etc.)?	1...Never (0 times in the last 4 weeks)<br>2...Rarely (1–2 times in the last 4 weeks)<br>3...Sometimes (3–10 times in the last 4 weeks)<br>4...Often (11-20 times in the last 4 weeks)<br>5….Always (More than 20 times in the last 4 weeks)<br>99….Don't know<br>88…Not applicable/I don't have this
HWISE12	In the last 4 weeks, how frequently has there not been enough water in the household to **wash clothes**?	1...Never (0 times in the last 4 weeks)<br>2...Rarely (1–2 times in the last 4 weeks)<br>3...Sometimes (3–10 times in the last 4 weeks)<br>4...Often (11-20 times in the last 4 weeks)<br>5….Always (More than 20 times in the last 4 weeks)<br>99….Don't know<br>88…Not applicable/I don't have this
HWISE13	In the last 4 weeks, how frequently have you or anyone in your household had to **change what was being eaten** because there were problems with water (e.g. for washing foods, cooking, etc.)?	1...Never (0 times in the last 4 weeks)<br>2...Rarely (1–2 times in the last 4 weeks)<br>3...Sometimes (3–10 times in the last 4 weeks)<br>4...Often (11-20 times in the last 4 weeks)<br>5….Always (More than 20 times in the last 4 weeks)<br>99….Don't know<br>88…Not applicable/I don't have this
HWISE14	In the last 4 weeks, how frequently have you or anyone in your household had to go **without**	1...Never (0 times in the last 4 weeks)<br>2...Rarely (1–2 times in the last 4 weeks)<br>3...Sometimes (3–10 times in the last 4

For peer review only - http://bmjopen.bmj.com/site/about/guidelines.xhtml

	**washing hands** after **dirty activities (**e.g., defecating or changing diapers, cleaning animal dung) because of problems with water?	weeks)<br>4...Often (11-20 times in the last 4 weeks)<br>5….Always (More than 20 times in the last 4 weeks)<br>99….Don't know<br>88…Not applicable/I don't have this
HWISE15	In the last 4 weeks, how frequently have you or anyone in your household not **washed the faces and hands of children** because of problems with water?	1...Never (0 times in the last 4 weeks)<br>2...Rarely (1–2 times in the last 4 weeks)<br>3...Sometimes (3–10 times in the last 4 weeks)<br>4...Often (11-20 times in the last 4 weeks)<br>5….Always (More than 20 times in the last 4 weeks)<br>99….Don't know<br>88…Not applicable/I don't have this
HWISE16	In the last 4 weeks, how frequently have you or anyone in your household had to go without **washing their body** because of problems with water (e.g. not enough water, dirty, unsafe)?	1...Never (0 times in the last 4 weeks)<br>2...Rarely (1–2 times in the last 4 weeks)<br>3...Sometimes (3–10 times in the last 4 weeks)<br>4...Often (11-20 times in the last 4 weeks)<br>5….Always (More than 20 times in the last 4 weeks)<br>99….Don't know<br>88…Not applicable/I don't have this
HWISE17	In the last 4 weeks, how frequently has you or anyone in your household had to **change schedules/plans** due to problems with your water situation, such as problems getting or distributing water within the household? Activities that may have been interrupted include caring for others, doing household chores, etc.	1...Never (0 times in the last 4 weeks)<br>2...Rarely (1–2 times in the last 4 weeks)<br>3...Sometimes (3–10 times in the last 4 weeks)<br>4...Often (11-20 times in the last 4 weeks)<br>5….Always (More than 20 times in the last 4 weeks)<br>99….Don't know<br>88…Not applicable/I don't have this
HWISE18	In the last 4 weeks, how frequently have problems with water prevented you or anyone in your household from **attending social or cultural events** (e.g. church, funerals, community gatherings, cultural practices, etc.)?	1...Never (0 times in the last 4 weeks)<br>2...Rarely (1–2 times in the last 4 weeks)<br>3...Sometimes (3–10 times in the last 4 weeks)<br>4...Often (11-20 times in the last 4 weeks)<br>5….Always (More than 20 times in the last 4 weeks)<br>99….Don't know<br>88…Not applicable/I don't have this

For peer review only - http://bmjopen.bmj.com/site/about/guidelines.xhtml

HWISE19	In the last 4 weeks, how frequently have you or anyone in your household drank water that **looked, tasted, and/or smelled bad**?	1...Never (0 times in the last 4 weeks)<br>2...Rarely (1–2 times in the last 4 weeks)<br>3...Sometimes (3–10 times in the last 4 weeks)<br>4...Often (11-20 times in the last 4 weeks)<br>5….Always (More than 20 times in the last 4 weeks)<br>99….Don't know<br>88…Not applicable/I don't have this
HWISE20	In the last 4 weeks, how frequently have you or anyone in your household drank water that you thought was **unsafe**?	1...Never (0 times in the last 4 weeks)<br>2...Rarely (1–2 times in the last 4 weeks)<br>3...Sometimes (3–10 times in the last 4 weeks)<br>4...Often (11-20 times in the last 4 weeks)<br>5….Always (More than 20 times in the last 4 weeks)<br>99….Don't know<br>88…Not applicable/I don't have this
HWISE21c	If you needed to borrow water, from how many people could you borrow water?	
HWISE21	In the last 4 weeks, how frequently have you or anyone in your household asked to **borrow** water from other people?	1...Never (0 times in the last 4 weeks)<br>2...Rarely (1–2 times in the last 4 weeks)<br>3...Sometimes (3–10 times in the last 4 weeks)<br>4...Often (11-20 times in the last 4 weeks)<br>5….Always (More than 20 times in the last 4 weeks)<br>99….Don't know<br>88…Not applicable/I don't have this
HWISE21a	From whom? Please list all the ways you are connected to these people. For example, neighbor, family member, or both neighbor and family member.	Person #1:<br><br>Person #2:<br><br>Person #3:
HWISE21b	What were you expected to give in return?	
HWISE30	In the last 4 weeks, how frequently have you or anyone in your household loaned water to anyone?	1...Never (0 times in the last 4 weeks)<br>2...Rarely (1–2 times in the last 4 weeks)<br>3...Sometimes (3–10 times in the last 4 weeks)<br>4...Often (11-20 times in the last 4 weeks)<br>5….Always (More than 20 times in the last 4

		weeks)<br>99….Don't know<br>88…Not applicable/I don't have this
HWISE22	In the last 4 weeks, how frequently did you or anyone in your household have problems with water that caused **difficulties with neighbors, water providers, or others** in the community?	1...Never (0 times in the last 4 weeks)<br>2...Rarely (1–2 times in the last 4 weeks)<br>3...Sometimes (3–10 times in the last 4 weeks)<br>4...Often (11-20 times in the last 4 weeks)<br>5….Always (More than 20 times in the last 4 weeks)<br>99….Don't know<br>88…Not applicable/I don't have this
HWISE23	In the last 4 weeks, how frequently did you or anyone in your household have problems with water that caused **difficulties within your household**?	1...Never (0 times in the last 4 weeks)<br>2...Rarely (1–2 times in the last 4 weeks)<br>3...Sometimes (3–10 times in the last 4 weeks)<br>4...Often (11-20 times in the last 4 weeks)<br>5….Always (More than 20 times in the last 4 weeks)<br>99….Don't know<br>88…Not applicable/I don't have this
HWISE24	In the last 4 weeks, how frequently did you or anyone in your household feel **angry** about your water situation?	1...Never (0 times in the last 4 weeks)<br>2...Rarely (1–2 times in the last 4 weeks)<br>3...Sometimes (3–10 times in the last 4 weeks)<br>4...Often (11-20 times in the last 4 weeks)<br>5….Always (More than 20 times in the last 4 weeks)<br>99….Don't know<br>88…Not applicable/I don't have this
HWISE25	In the last 4 weeks, how frequently has there not been **as much water to drink** as you would like for you or anyone in your household?	1...Never (0 times in the last 4 weeks)<br>2...Rarely (1–2 times in the last 4 weeks)<br>3...Sometimes (3–10 times in the last 4 weeks)<br>4...Often (11-20 times in the last 4 weeks)<br>5….Always (More than 20 times in the last 4 weeks)<br>99….Don't know<br>88…Not applicable/I don't have this
HWISE26	In the last 4 weeks, how frequently have you or anyone in your household gone to **sleep thirsty**	1...Never (0 times in the last 4 weeks)<br>2...Rarely (1–2 times in the last 4 weeks)<br>3...Sometimes (3–10 times in the last 4 weeks)

For peer review only - http://bmjopen.bmj.com/site/about/guidelines.xhtml

	because there wasn't any water to drink?	4...Often (11-20 times in the last 4 weeks)<br>5….Always (More than 20 times in the last 4 weeks)<br>99….Don't know<br>88…Not applicable/I don't have this
HWISE27	In the last 4 weeks, how frequently has there been **no useable or drinkable water** whatsoever in your household?	1...Never (0 times in the last 4 weeks)<br>2...Rarely (1–2 times in the last 4 weeks)<br>3...Sometimes (3–10 times in the last 4 weeks)<br>4...Often (11-20 times in the last 4 weeks)<br>5….Always (More than 20 times in the last 4 weeks)<br>99….Don't know<br>88…Not applicable/I don't have this
HWISE28	In the last 4 weeks, how frequently have you or anyone in your household thought of **moving dwellings** because of the water situation there?	1...Never (0 times in the last 4 weeks)<br>2...Rarely (1–2 times in the last 4 weeks)<br>3...Sometimes (3–10 times in the last 4 weeks)<br>4...Often (11-20 times in the last 4 weeks)<br>5….Always (More than 20 times in the last 4 weeks)<br>99….Don't know<br>88…Not applicable/I don't have this
HWISE29	In the last 4 weeks, how frequently have problems with water caused you or anyone in your household to **feel ashamed**/excluded/stigmatized?	1...Never (0 times in the last 4 weeks)<br>2...Rarely (1–2 times in the last 4 weeks)<br>3...Sometimes (3–10 times in the last 4 weeks)<br>4...Often (11-20 times in the last 4 weeks)<br>5….Always (More than 20 times in the last 4 weeks)<br>99….Don't know<br>88…Not applicable/I don't have this

For peer review only - http://bmjopen.bmj.com/site/about/guidelines.xhtml

4. Water Access<br>*Now I would like to learn how and where you acquire water.*		
Wat1	What is currently the **primary source** of drinking water for your household? (Choose only ONE)	1…Piped water<br>2…Stand pipe<br>3…Borehole/tubewell<br>4…Protected dug well<br>5…Unprotected dug well<br>6…Protected spring<br>7…Unprotected spring<br>8…Rainwater collection<br>9…Small water vendor<br>10…Tanker truck<br>11…Bottled water<br>12…Bagged/sachet water<br>13…Surface water (pond, river, lake)<br>14…Other person ____________<br>15…Other_________
Wat2	What is currently the **primary source** of non- drinking water for your household? (Choose only ONE)	1…Piped water<br>2…Stand pipe<br>3…Borehole/tubewell<br>4…Protected dug well<br>5…Unprotected dug well<br>6…Protected spring<br>7…Unprotected spring<br>8…Rainwater collection<br>9…Small water vendor<br>10…Tanker truck<br>11…Bottled water<br>12…Bagged/sachet water<br>13…Surface water (pond, river, lake)<br>14…Other person ____________<br>15…Other_________
Wat3	How long (in minutes) does it take to go to the water source, get water and come back (including wait time)? *(If water source is in household/compound, record 00 minutes)*	____________ minutes
Wat4	How many trips in total are made to this site per week (not including household/compound?)?	__________ trips
Wat4a	Have you ever been injured while fetching water?	0...No<br>1…Yes
Wat4b	If so, how?	
Wat5	In the past 4 weeks, approximately how much money did you spend on getting	Units: ______ Amount: ________

For peer review only - http://bmjopen.bmj.com/site/about/guidelines.xhtml

	water for your household?	
Wat6	In the past 4 weeks, was drinking water for your household typically treated in any way to make it safer?	0…No<br>1…Yes<br>DK…Don't know
Wat7	What is the primary way that your household treats your **drinking** water?	1…Do not treat it<br>2…Boil<br>3…Filter<br>4…Add chemicals<br>5…Other (Specify):<br>______________________________
Wat8	In the past 4 weeks, how much money did you spend to treat water (including money for chemicals to treat water)?	Currency: ______ Amount: ________

**5. Water Quantity, Utility and Stability**<br>*Now I would like to ask you questions on the quantity of water you have in your household and the amount you drink.*		
Wat9	What is the estimate of the current amount of drinking water (liters) stored in your household?	________ Liters
Wat10	What is the estimate of the current amount of non-drinking water (liters) stored in your household?	________ Liters
Wat11	Which of the months in the year does your household mostly experience **water shortage** (circle all months)?	1…January 7…July<br>2…February 8…August<br>3…March 9…September<br>4…April 10…October<br>5…May 11…November<br>6…June 12…December
Wat12	Which of the months in the year does your household have **excessive amounts** of water (tick the specific months)?	1…January 7…July<br>2…February 8…August<br>3…March 9…September<br>4…April 10…October<br>5…May 11…November<br>6…June 12…December
Wat13	What times of day does your household experience water shortages?	0…None<br>1…Morning (Sunrise (6:00am) to 11:59am)<br>2…Afternoon (12:00 – 5:00pm)<br>3…Evening (5:01 – 8:00pm)<br>4…Night (8:01pm – 5:59am)
Wat14	What do you see as the main **cause of problems with water** in your area?	
Wat15	What do you do when you **don't have enough water** and don't have enough money to buy water?	

6. Food Insecurity (HFIAS)

Now I'm going to ask you about your experiences with access to food in the last four weeks. I will ask you about the frequencies that you have experienced a few situations, and I want you to tell me how frequently it has happened in the last four weeks.

Code Name	Questions	Coding Classification
FI1	frequently did you worry that your household would not have enough food?	1…Never (0 times in the last 4 weeks)<br>2…Rarely (1 – 2 times in the last 4 weeks)<br>3…Sometimes (3 – 10 times in the last 4 weeks)<br>4…Often (More than 10 times in the last 4 weeks)<br>9…Don't know
FI2	In the past four weeks, how frequently were you or any household member not able to eat foods you preferred because you couldn't obtain them because of a lack of resources (such as money, business, land, or any other thing that you would require to help you obtain other types of food)?	1…Never (0 times in the last 4 weeks)<br>2…Rarely (1 – 2 times in the last 4 weeks)<br>3…Sometimes (3 – 10 times in the last 4 weeks)<br>4…Often (More than 10 times in the last 4 weeks)<br>9…Don't know
FI3	In the past four weeks, how frequently did you or any household member have to eat a limited variety of food due to lack of resources (such as money, business, land, or any other thing that you would require to help you obtain other types of food)?	1…Never (0 times in the last 4 weeks)<br>2…Rarely (1 – 2 times in the last 4 weeks)<br>3…Sometimes (3 – 10 times in the last 4 weeks)<br>4…Often (More than 10 times in the last 4 weeks)<br>9…Don't know
FI4	In the past four weeks, how frequently did you or any household member have to eat some foods that you really did not want to eat because of a lack of resources to obtain other types of food?	1…Never (0 times in the last 4 weeks)<br>2…Rarely (1 – 2 times in the last 4 weeks)<br>3…Sometimes (3 – 10 times in the last 4 weeks)<br>4…Often (More than 10 times in the last 4 weeks)<br>9…Don't know
FI5	In the past four weeks, how frequently did you or any household member have to eat a smaller meal than you felt you needed, meaning a little amount of food that did not	1…Never (0 times in the last 4 weeks)<br>2…Rarely (1 – 2 times in the last 4 weeks)<br>3…Sometimes (3 – 10 times in the last 4 weeks)<br>4…Often (More than 10 times in the last 4

For peer review only - http://bmjopen.bmj.com/site/about/guidelines.xhtml

	satisfy you, because there was not enough food?	weeks)<br>9…Don't know
FI6	How many meals do you think you should eat in a day?	
FI7	In the past four weeks, how frequently did you or anyone in your household have to eat fewer meals in a day because there was not enough food?	1…Never (0 times in the last 4 weeks)<br>2…Rarely (1 – 2 times in the last 4 weeks)<br>3…Sometimes (3 – 10 times in the last 4 weeks)<br>4…Often (More than 10 times in the last 4 weeks)<br>9…Don't know
FI8	In the past four weeks, how frequently was there ever no food to eat of any kind in your household because of lack of resources to get food?	1…Never (0 times in the last 4 weeks)<br>2…Rarely (1 – 2 times in the last 4 weeks)<br>3…Sometimes (3 – 10 times in the last 4 weeks)<br>4…Often (More than 10 times in the last 4 weeks)<br>9…Don't know
FI9	In the past four weeks, how frequently did you or any household member go to sleep at night hungry because there was not enough food?	1…Never (0 times in the last 4 weeks)<br>2…Rarely (1 – 2 times in the last 4 weeks)<br>3…Sometimes (3 – 10 times in the last 4 weeks)<br>4…Often (More than 10 times in the last 4 weeks)<br>9…Don't know
FI10	In the past four weeks, how frequently did you or any household member go hungry for a whole day and night because of limited food in the house?	1…Never (0 times in the last 4 weeks)<br>2…Rarely (1 – 2 times in the last 4 weeks)<br>3…Sometimes (3 – 10 times in the last 4 weeks)<br>4…Often (More than 10 times in the last 4 weeks)<br>9…Don't know

**7. Perceived Stress Scale**<br>*The questions in this scale ask you about your feelings and thoughts during the last month. In each case, indicate how often you felt or thought a certain way.*		
PS1	In the past four weeks, how often have you felt that you were unable to control the important things in your life?	1…Never (0 times in the last 4 weeks)<br>2…Almost Never (1-2 times in the last 4 weeks)<br>3…Sometimes (3-10 times in the last 4 weeks)<br>4…Fairly Often (11-20 times in the last 4 weeks)<br>5…Very Often (More than 20 times in the last 4 weeks)
PS2	In the last month, how often have you felt confident about your ability to handle your personal problems?	1…Never (0 times in the last 4 weeks)<br>2…Almost Never (1-2 times in the last 4 weeks)

For peer review only - http://bmjopen.bmj.com/site/about/guidelines.xhtml

		3…Sometimes (3-10 times in the last 4 weeks)<br>4…Fairly Often (11-20 times in the last 4 weeks)<br>5…Very Often (More than 20 times in the last 4 weeks)
PS3	In the last month, how often have you felt that things were going your way?	1…Never (0 times in the last 4 weeks)<br>2…Almost Never (1-2 times in the last 4 weeks)<br>3…Sometimes (3-10 times in the last 4 weeks)<br>4…Fairly Often (11-20 times in the last 4 weeks)<br>5…Very Often (More than 20 times in the last 4 weeks)
PS4	In the last month, how often have you felt difficulties were piling up so high that you could not overcome them?	1…Never (0 times in the last 4 weeks)<br>2…Almost Never (1-2 times in the last 4 weeks)<br>3…Sometimes (3-10 times in the last 4 weeks)<br>4…Fairly Often (11-20 times in the last 4 weeks)<br>5…Very Often (More than 20 times in the last 4 weeks)

**8. Infant feeding**<br>*This question will ask you about infant feeding and how it can be impacted by the water situation in your area.*		
BF1	Can you tell me some ways that the water situation here affects how infants (under 12 months of age) are fed? (*Interviewer, prompt for three ways.)*	1.<br><br>2.<br><br>3.

For peer review only

2a. Socio Demographic Questions (continued)<br>*We are now going to ask you three final questions about your standing in your community.*		
SD8	What is your current occupation or how do you earn money?	
SD9	Here is a picture of a ladder. Please think of this ladder as representing the socioeconomic standing of people ***in your community.***<br><br>At the top of the ladder are those who are best off; they have the most money, the most education, the most respected jobs. At the bottom are people who have the least money, least education, and least jobs.<br><br>Where would you place yourself on this ladder? Please touch the rung/step.<br><br>Interviewer, write the corresponding number here: ________	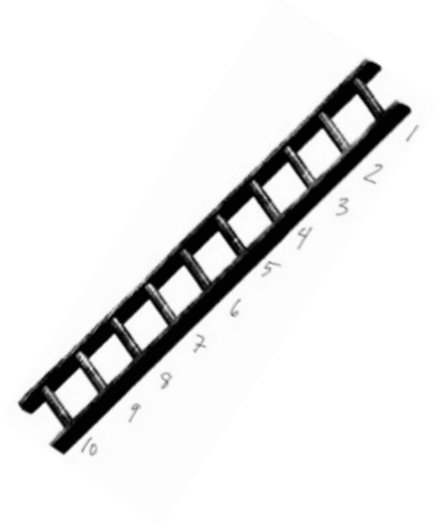
SD11	Here is a picture of a ladder. Please think of this ladder as representing the water situation of people ***in your community.***<br><br>At the top of the ladder are those who have the best water situation; they can easily get enough water for everything that they need and never have too much. At the bottom are people who have the most problems with water.<br><br>Where would you place yourself on this ladder? Please touch the rung/step.<br><br>Interviewer, write the corresponding number here: ________	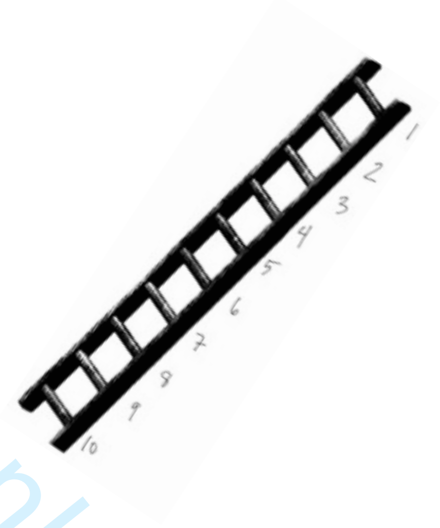
SD10	What is your primary monthly income? (/month)	Units______ Amount _________

Wrap-up Question: Is there anything else you would like to share about your experiences with water for your household and how this affects your life?

Do you have any questions for us? ***Thank you*** *for participating in this survey.*

For peer review only

Data Quality: For the interviewer to complete<br>*Please give your assessment on the quality and reliability of the data you have collected in this survey.*		
DQ01	Did the respondent show any of the following? *(check all that apply):*	___ Mistrust of you or the study<br>___ Dishonesty, lying, or inventing responses that didn't seem true<br>___ Fear of you or the study<br>___ Hostility, anger or resentment<br>___ Evasion or trying to avoid answering
DQ02	Were there any interruptions or distractions?	0…No<br>1…Yes, but I don't think it influenced responses<br>2…Yes, and I think it influenced the answers
DQ03	What is your overall assessment of the quality of the data in this survey?	0…**Excellent**. The respondent understood the survey and was engaged<br>and there were no interruptions<br>1…**Just okay**. The participant may not have understood all or part of<br>the survey well or there were some interruptions.<br>2…**Suspect**. The participant misunderstood the survey or did not<br>participate well or there were many interruptions.
DQ04	The quality of the data from this interview is really important to us. Please explain your answers above, in terms of how the data may have been affected.<br>Is there anything else we need to know?	

For peer review only

Household Water Insecurity Experiences

HWISE 3.0 Survey Sources

Topic	Brief Description	Source
Socio-demographics	Neighborhood, region, district/residence	Adapted from UNICEF Multiple Indicator Cluster Survey (MICS)[1]
	Role in household	
	Gender of Household head/respondent	
	Relationship status	Adapted from Demographic and Health Surveys (DHS) Household Questionnaire[2]
	Age of household head/respondent	
	Person responsible for collecting water in the household	Adapted from WHO & UNICEF Core Questions on Drinking Water and Sanitation for Household Surveys[3]
	Household size (# of adults & # of kids)	Adapted from UNICEF Multiple Indicator Cluster Survey (MICS)[1]
	Type of housing	Developed by investigators
	Religion and denomination	
Household Water Insecurity Experiences (HWISE) Scale	30-item household water insecurity experiences scale	Developed from extensive literature review[5] and team's knowledge of water insecurity[4,9-12]
Water sharing	Borrowing/loaning, what are you expected to give in return, from whom do you borrow?	Developed by investigators
Water Quality	Source of primary drinking water (WHO categories for improved & unimproved sources)	Adapted from WHO & UNICEF Core Questions on Drinking Water and Sanitation for Household Surveys[3]
	Source of primary non-drinking water (WHO categories for improved & unimproved sources)	
	Assessment of drinking water to be safe or unsafe	Developed by investigators
	Participants treating their water	Adapted from Demographic and Health Surveys (DHS) Household Questionnaire[2]
Water Accessibility	The amount of money spent by the household in water collection	Developed by investigators
	Estimate the time spent in collecting water from water source	Adapted from WHO & UNICEF Core Questions on Drinking Water and Sanitation for Household Surveys[3]
	Frequency of water collection	
Water Quantity	Amount of drinking water stored in household (L)	Developed by investigators
	Amount of non-drinking water stored in household (L)	
Water Utility	Amount of water drank in a day (L)	Developed by investigators
Water Stability/Reliability	Which of the months in a year do households experience water excess and scarcity?	Developed by investigators
	Which times of day do households experience water scarcity?	

For peer review only

**Food Insecurity**	9-item estimate via Household Food Insecurity Access Scale (HFIAS).	Adapted from the Household Food Insecurity Access Scale (HFIAS)[8]
**Perceived Stress**	4-item Estimate via Cohen's Perceived Stress Scale.	Adapted from the Perceived Stress Scale[7]
**Infant Feeding**	1 open-ended question on perceptions of how water insecurity may affect infant & young child feeding	Developed by investigators
**Socioeconomic Status**	Open-ended question about current occupation	Developed by investigators
	Likert ladder with degree of participants socio-economic status (scaled 1 to 10, with 1 being the best off, most educated, most money, and the most respected job; at the bottom participants with less money, education, least respected jobs)	Adapted from The MacArthur Scale of Subjective Social Status[8]
	Household monthly income	Developed by investigators
**Data Quality**	4-items on interviewer-assessed quality of responses	Developed by investigators

1.UNICEF. Multiple Indicator Cluster Survey (MICS): Household Questionnaire [Internet]. 2017. Available from: http://mics.unicef.org/tools#survey-design
2.The DHS Program, USAID. Demographic and Health Surveys (DHS): Questionnaire Modules [Internet]. 2015. Available from: https://dhsprogram.com/publications/publication-dhsqm-dhs-questionnaires-and-manuals.cfm
3.WHO, UNICEF. Core Questions on Drinking Water and Sanitation for Household Surveys [Internet]. Geneva : World Health Organization; 2006. Available from: https://extranet.who.int/iris/restricted/handle/10665/43489
4.Boateng G, Collins SM, Mbullo P, Wekesa P, Onono M, Neilands TB, et al. A Novel Household Water Insecurity Scale: Procedures and Psychometric Analysis Among Postpartum Women in Western Kenya. Biorxiv 10.1101/294298.
5.Jepson WE, Wutich A, Collins SM, Boateng GO, Young SL. Progress in household water insecurity metrics: a cross-disciplinary approach. WIREs Water. 2017 Apr 11;4(3):e1214-21.
6.Coates J, Swindale A, Bilinsky P. Household food insecurity access scale (HFIAS) for measurement of food access: Indicator guide. Washington DC: Food and Nutrition Technical Assistance Project; 2007.
7.Cohen S, Kamarck T, Mermelstein R. A global measure of perceived stress. J Health Soc Behav. 1983;24(4):385–96.
8.Adler N, Stewart J, Psychosocial Working Group. The MacArthur Scale of Subjective Social Status [Internet]. MacArthur Scale of Subjective Social Status. 2007. Available from: http://www.macses.ucsf.edu/research/psychosocial/subjective.php
9.Wutich A, Brewis A. Food, Water, and Scarcity. Current Anthropology. 2014;55(4):444–68.
10.Jepson W. Measuring no-win waterscapes: Experience-based scales and classification approaches to assess household water security in *colonias* on the US--Mexico border. Geoforum. 2014;51(C):107–20.
11.Wutich A, Ragsdale K. Water insecurity and emotional distress: coping with supply, access, and seasonal variability of water in a Bolivian squatter settlement. Social science & medicine. 2008;67(12):2116–25.
12.Krumdieck N, Collins S, Wekesa P, Mbullo P, Boateng G, Onono M, et al. Household water insecurity is associated with a range of negative consequences among pregnant Kenyan women of mixed HIV status. Journal of Water and Health. 2016 Jul 25.

For peer review only

Household Water Insecurity Experiences

HWISE Data Protocol

Data cleaning procedure and notes for using the HWISE data

Purposes of Data Cleaning

I. Identify Aberrant Data

A. There are numerous instances in which data may be entered incorrectly or missing when it should not be; for each of the following situations, the protocol is to 1) contact PIs to see if the data can be recovered and/or corrected and, if not, 2) either update the aberrant data with a reasonable value or as missing

- Categorical variables that are not within reasonable or defined bounds (e.g. age is reported to be 500)
- Data that are logically incongruent (e.g. respondent reports that he/she does not treat water but then lists a non-zero amount spent on water treatment)
- Missing data

II. De-string Numeric Variables

A. String variables (i.e. those with with non-numeric characters) are updated to be numerical

III. Split Multiple-Select Variables

A. To make multiple-select responses analyzable, they are split into multiple responses (e.g. months of water shortage are split into 12 variables)

IV. Remove Duplicates

A. If multiple entries are submitted for the same participant, these are flagged and only the most up-to-date submission is retained

V. Recode Values

A. Values may be recoded to more clearly flag data that should later be changed to missing for analysis

- 999 = “Don’t know”
- 888 = “Not applicable”
- 555 = “Refuse to answer”

B. Multiple-select response options sometimes vary across sites (e.g. ‘1’ is piped water in one site but ‘7’ in another); to account for this in aggregated datasets, values are recoded to be harmonious across sites

VI. Label Data

A. Add variable labels to describe what a variable represents

For peer review only - http://bmjopen.bmj.com/site/about/guidelines.xhtml

B. Add value labels to categorical variables to convey what each value represents (e.g. ‘0’ is male)

VII. Generate New Variables

A. Variables that are of broad interest to the group are generated based on other items within the dataset (e.g. creating a food insecurity score based on the FIAS questions)

VIII. Reorder Variables

A. Throughout the data entry and cleaning process, variables may be shifted around; these are reordered to match the order in the original survey

Stages of Data Cleaning

I. Cleaning of Site-Specific Data

A. Raw .csv data is imported and saved as a Stata dataset (.dta file)
B. All variables are then cleaned following the above guidelines; this includes variables unique to specific sites (e.g. ward in Ethiopia)
   - All changes made to aberrant data are documented in the data dictionary (see below)
C. Once cleaned, the dataset is saved

II. Cleaning of Aggregated Data

A. Cleaned datasets for each site are appended together
B. Site-specific variables are dropped
C. Multiple-select response options that vary across sites (e.g. ‘1’ is piped water in one site but ‘7’ in another) are recoded so responses across all sites are comparable
D. Questions related to money are converted to USD (exchange rate for each site based on date of last interview at the site and pulled from https://www.oanda.com/currency/converter/)
E. Once cleaned, the dataset is saved
F. Code replacing 555, 888, 999 with missing is then executed; the dataset is saved again

Using HWISE Data

I. Data Dictionary

For peer review only - http://bmjopen.bmj.com/site/about/guidelines.xhtml

A. The first tab ("Summary") provides a broad overview of the modules within the survey; blue text hyperlinks to the respective module in the more in-depth "Dictionary" tab
B. The "Dictionary" tab describes what each variables represents, data type, and the appropriate range of responses
C. The "Data Errors" section lists each error encountered while cleaning the site-specific data, as well as any corrective action taken; if you discover an error in the data that is not listed here, please contact the Northwestern Team

II. Determining Which Aggregated Dataset to Use

A. One dataset retains 555 (refuse to answer), 888 (not applicable), and 999 (don't know); this is best used for understanding the range of responses
B. The other dataset replaces 555, 888, and 999 with missing; it is best to use this dataset when performing analyses

For peer review only - http://bmjopen.bmj.com/site/about/guidelines.xhtml

Development and validation protocol for an instrument to measure household water insecurity across cultures and ecologies: the Household Water InSecurity Experiences (HWISE) Scale

Journal:	*BMJ Open*
Manuscript ID	bmjopen-2018-023558.R1
Article Type:	Protocol
Date Submitted by the Author:	10-Sep-2018
Complete List of Authors:	Young, Sera; Northwestern University, Anthropology<br>Collins, Shalean ; Northwestern University, Anthropology<br>Boateng, Godfred; Northwestern University, Anthropology<br>Neilands, Torsten; University of California San Francisco, School of Medicine<br>Jamaluddine, Zeina; American University of Beirut, Center for Research on Population and Health<br>Miller , Joshua; Northwestern University, Anthropology<br>Brewis, Alex; Arizona State University, School of Human Evolution and Social Change<br>Frongillo, EA; University of South Carolina, Department of Health Promotion, Education, and Behavior<br>Jepson, Wendy; Texas A&M University Department of Geography<br>Melgar-Quiñonez, Hugo; McGill University Institute, Institute for Global Food Security<br>Schuster, Roseanne; Arizona State University, School of Human Evolution and Social Change<br>Stoler, JB; University of Miami, Department of Geography<br>Wutich, Amber; Arizona State University, School of Human Evolution and Social Change
**Primary Subject Heading**:	Global health
Secondary Subject Heading:	Research methods, Epidemiology, Public health
Keywords:	survey, water, household, scale development, water insecurity, protocol

SCHOLARONE™
Manuscripts

Development and validation protocol for an instrument to measure household water insecurity across cultures and ecologies: the Household Water InSecurity Experiences (HWISE) Scale

Sera L. Young[1], Shalean M. Collins[1], Godfred O. Boateng[1], Torsten B. Neilands[2], Zeina Jamaluddine[3], Joshua D. Miller[1], Alexandra Brewis[6], Edward A. Frongillo[5], Wendy E. Jepson[8], Hugo Melgar-Quiñonez[4], Roseanne C. Schuster[6], Justin Stoler[7], Amber Wutich[6], the HWISE Research Coordination Network[1]

[1]Northwestern University, Department of Anthropology & Institute for Policy Research, Evanston, IL USA
[2]University of California, San Francisco, School of Medicine, San Francisco, CA USA
[3]Center for Research on Population and Health, American University of Beirut, Beirut, Lebanon
[4]Institute for Global Food Security, McGill University, Montreal, QC, Canada
[5]University of South Carolina, Department of Health Promotion, Education, and Behavior, Columbia, SC USA
[6]Arizona State University, School of Human Evolution and Social Change, Tempe, AZ USA
[7]University of Miami, Department of Geography, Coral Gables, FL USA
[8]Texas A&M University, Department of Geography, College Station, TX USA

Corresponding Author
Sera L. Young
1819 Hinman Ave
Evanston, IL 60208
sera.young@northwestern.edu

[1] HWISE Research Coordination Network authors: Ellis Adams, Farooq Ahmed, Mallika Alexander, Mobolanle Balogun, Michael Boivin, Genny Carrillo, Kelly Chapman, Stroma Cole, Hassan Eini-Zinab, Jorge Escobar-Vargas, Matthew Freeman, Hala Ghattas, Ashley Hagaman, Nicola Hawley, Kenneth Maes, Jyoti Mathad, Patrick Mbullo Owour, Javier Moran, Nasrin Omidvar, Amber Pearson, Asher Rosinger, Luisa Samayoa-Figueroa, Ernesto Sánchez-Rodriguez, Jader Santos, Marriane V. Santoso, Sonali Srivastava, Chad Staddon, Andrea Sullivan, Yihenew Tesfaye, Nathaly Triviño-León, Alex Trowell, Desire Tshala-Katumbay, Raymond Tutu, Felipe Uribe-Salas, Elizabeth Wood, Cassandra Workman

For peer review only - http://bmjopen.bmj.com/site/about/guidelines.xhtml

ABSTRACT

Introduction: A wide range of water-related problems contribute to the global burden of disease. Despite the many plausible consequences for health and wellbeing, there is no validated tool to measure water insecurity equivalently at the individual- or household-levels across varying cultural and ecological settings. Accordingly, we are developing the Household Water InSecurity Experiences (HWISE) Scale to measure household-level water insecurity in multiple contexts.

Methods and analysis: After domain specification and item development, items were assessed for both content and face validity. Retained items are being asked in surveys in 28 sites globally in which water-related problems have been reported (e.g. shortages, excess water, issues with quality), with a target of at least 250 participants from each site. Scale development will draw on analytic methods from both Classic Test and Item Response Theories, and include item reduction and factor structure identification and replication. Scale evaluation will entail assessments of reliability, and predictive, convergent, and discriminant validity, as well as the assessment of differentiation across known groups.

Ethics and dissemination: Study activities received necessary ethical approvals from institutional review bodies relevant to each site. We anticipate that the final HWISE Scale will be completed by late 2018 and made available through open-access publication. Associated findings will be disseminated to public health professionals, scientists, practitioners, and policymakers through peer-reviewed journals, scientific presentations, and meetings with various stakeholders. Measures to quantify household food insecurity have transformed policy, research, and humanitarian aid efforts globally, and we expect that an analogous measure for water insecurity will be similarly impactful.

Keywords: water insecurity, survey, water, household, scale development

For peer review only - http://bmjopen.bmj.com/site/about/guidelines.xhtml

For peer review only

ARTICLE SUMMARY

Strengths and limitations of this study

*This study is based on rigorous, multi-disciplinary formative research on water insecurity by anthropologists, geographers, nutritionists, statisticians, and epidemiologists, among others.

*Data on household water insecurity experiences are being collected in 28 sites across four continents by local partners in widely varying ecological, cultural, and political settings.

*Analytic methods from both Classic Test and Item Response Theories will be used to develop and evaluate the eventual scale.

*The HWISE Scale will be validated for assessing water insecurity in low- and middle-income countries. Additional scale assessments necessary for validation in high income countries are planned.

3759 words

INTRODUCTION

Water insecurity, the inability to "access and benefit from affordable, adequate, reliable and safe water for wellbeing and a healthy life",[1] has manifold adverse effects on physical and psychosocial health;[2,3] undermines productivity;[4] triggers and perpetuates domestic, social, and political tensions and conflicts;[5,6] and reinforces environmental and social inequities.[7] Water insecurity has been shown to co-occur with food insecurity, malnutrition, and communicable diseases, and to produce syndemics, or systemically exacerbating epidemics[8,9], much like food insecurity and HIV.[10] Further, water insecurity is projected to worsen in many regions due to climate change and increased inequalities in resource distribution.[7]

However, we do not know with any precision how many households or individuals globally are affected by water insecurity. Estimates of available surface water derived from satellite imagery suggest that 4 billion people worldwide experience severe water scarcity for at least one month of every year[11], and this is likely an underestimation given issues with infrastructure and access. Additionally, chronic flooding[12] and poor water quality[13] mean that many more individuals are experiencing water insecurity. Currently, measures of water at the national, regional, and community levels are used and are referred to as indicators of water scarcity, water

For peer review only - http://bmjopen.bmj.com/site/about/guidelines.xhtml

poverty, or water security.[14–17] These measures do not capture the range and granularity of how households experience water insecurity, including factors such as perceptions of quality[18], instances of water excess[19], or perceived consequences for psychosocial[20,21] and physical health and wellbeing[22]. Further, while household-level scales to measure water insecurity have been developed for several sites, for example, in the United States,[23] Bolivia,[20] Uganda,[24] Ethiopia,[21] and Kenya,[8] their comparability, comprehensiveness, and applicability to other sites have not been systematically investigated or validated.

As such, a comprehensive, validated scale to measure the experiences of household or individual water insecurity would enable researchers, practitioners, and policymakers to: improve estimates of water insecurity prevalence, identify factors that shape this phenomenon, recognize direct consequences of water insecurity, understand how to more effectively distribute resources, evaluate the impacts and cost-effectiveness of interventions, and monitor progress toward the Sustainable Development Goals.[25] Indeed, in March 2018, the UN's High-Level Panel on Water cited lack of data on water in many parts of the world as a major challenge, and the need for better data on water as one of nine priority actions.[26] Given that measures of household food insecurity (e.g. Latin American and Caribbean Food Security Scale,[27] Household Food Insecurity Access Scale,[28] Food Insecurity Experience Scale[29]) have proven vital to implementation and evaluation of policy and programs[30–32], development of an analogous water insecurity scale is overdue and urgently needed, particularly for assessing water insecurity in low- and middle-income settings where household water problems tend to be most pronounced and frequent.

Therefore, our objective is to develop and validate the first household water insecurity scale with broad applicability across low- and middle-income settings. The Household Water

For peer review only - http://bmjopen.bmj.com/site/about/guidelines.xhtml

InSecurity Experiences (HWISE, pronounced *H-wise*) Research Coordination Network (RCN) was formed to facilitate the multi-country, collaborative data collection process required to validate the planned tool ("the HWISE Scale"). The HWISE RCN brings together a large team of anthropologists, geographers, public health practitioners, physicians, epidemiologists, statisticians, sociologists, nutritionists, *inter alia*, all of whom have experience with water insecurity, food insecurity, and/or scale development across a wide array of settings (http://www.hwise.org).

METHODS AND ANALYSIS

Phase 1: Item Development

1.1 Domain specification

Specifying the domains for a scale is the first step in item development (Table 1, 1.1).[33,34] The boundary of the domain of water insecurity, i.e. the underlying construct that the scale will attempt to measure, was based on extensive literature review[1] and draws on the team's expertise in water insecurity, e.g. [6,20,23,35]. We defined water insecurity as the condition where "affordability, reliability, adequacy, and safety [of water] is significantly reduced or unattainable so as to threaten or jeopardize well-being".[1]

A best practice is to clearly articulate sub-domains of the eventual scale, if they are known.[34,36] Although some sub-domains of water insecurity have been proposed [1,9,21,37], there is currently no consensus in the literature. Therefore, we will assess sub-domains as the scale is developed.

For peer review only - http://bmjopen.bmj.com/site/about/guidelines.xhtml

Table 1. Overview of planned methods and analyses for the development of the HWISE Scale. Adapted from [34].

Scale Development Activity	Procedures
**Phase 1: Item development**	
1.1 Domain specification	Literature review
1.2 Item generation	Literature review, Delphi methodology
1.3 Content validity	By target population: Two styles of cognitive interviews were used in the first 12 sites, building on Delphi methodology
1.4 Face validity	Pre-testing and debriefing with enumerators at each site
**Phase 2: Scale development**	
2.1 Data collection	Enumerator training and survey implementation
2.2 Item reduction	Drop items with cumulative missing cases >30% (i.e. "don't know," "non-applicable," or true missing responses)
	Assess the performance of each item's variation with other items in the scale using a correlation matrix; items with very low (<0.30) inter-item correlation coefficients and very low (<0.30) item-total correlation coefficients across multiple sites will be considered for deletion, as will items that misfit the model, i.e. with residual correlations >0.2
	Item reduction in Rasch paradigm: item severity, item discrimination test
2.3 Identify factor structure	Use factor analysis across multiple sites to test for factor structure; items with very low factor loadings (<0.30), split factor loadings (high factor loadings (>0.50) in two domains), and high residual variances will be considered for deletion
2.4 Assess measurement equivalence	Using multi-group confirmatory analysis (a form of measurement invariance) on data from multiple sites to test for exact invariance in the hypothetical scale; invariance will be assessed in terms of factor structure (configural model), factor loadings (matrix model), mean intercepts (scalar model), and factor means (strict model)
	Use confirmatory factor analysis alignment optimization to estimate the group-specific factor means and variances of scale items across all sites; it assesses proximate invariance of scale items across multiple sites
**Phase 3: Scale evaluation**	
3.1 Score scale items	Finalized scale items will be used in their unweighted form as sum scores, or in weighted form as factor scores
3.2 Assess reliability (internal consistency) of scale items	We will use Cronbach's alpha and the Rasch reliability statistic to test the internal consistency of the scale items within each site and aggregated across sites
3.3 Assess scale validity	We will measure predictive, convergent, and discriminant validity of the final scale items using criteria that were selected based on their strong theoretical relevance in the water insecurity literature; tests of water insecurity differences between "known groups" will also be performed

For peer review only - http://bmjopen.bmj.com/site/about/guidelines.xhtml

1.2 Item generation

We then identified initial scale items deductively, based on an extensive literature review of items used in prior site-specific household water insecurity scales[1] (Table 1, 1.2). This included team members' prior work implemented in *colonias* in Texas, United States;[23] a squatter settlement in Cochabamba, Bolivia;[20] in rural, peri-urban, and urban Kenya;[8] and elsewhere, including rural areas in Ethiopia[21] and Uganda[24]. Scale items include experiences of water insecurity that have consequences for psychosocial and physical health, nutrition, impacts on livelihoods and household economy, and agriculture (Supplemental Material 1: Overview of HWISE items).

Each question is phrased to elicit experiences within the prior four weeks or month (i.e. "In the last four weeks, how frequently have…"). This recall period was systematically determined using the Delphi method of consensus building with international and local experts in water insecurity, food insecurity, and scale development, and by comparing the responses in this recall period to a prospective daily recall of water insecurity experiences.[8] Items are ordered by what we expect to be increasing severity of water insecurity across access, reliability, adequacy, and safety. Response options are "never" (0 times), "rarely" (1-2 times), "sometimes" (3-10 times), "often" (11-20 times), "always" (more than 20 times), "not applicable", "don't know", or refused. Response intervals were also determined using the Delphi method.[8]

The initial set of 32 items is referred to as "HWISE 1.0". This set of items was modified slightly in August 2017 (cf. "Mid-study Evaluations") based on feedback received from consortium members, survey implementers, and other water security experts during a three-day conference at Northwestern University. Modifications included slight rephrasing of 18 items to improve comprehension by participants and elicit experiences related to water overabundance,

For peer review only - http://bmjopen.bmj.com/site/about/guidelines.xhtml

161 two questions were added in an effort to capture cultural components of water, and six items were eliminated for being too rare or idiosyncratic. The resultant set of 28 items is referred to as "HWISE 2.0" (Supplemental Material 1: Overview of HWISE items).

1.3 Content validity

Content validity (i.e. if items adequately measure the domain of interest; Table 1, 1.3) was assessed in the first eight sites through cognitive interviews with 12 purposively-selected individuals. Participants were asked to "think aloud" or "tell [the enumerator] about" their understanding of each of the HWISE items as they completed the pilot survey, and the interviewer recorded any issues and probes in detail on each as they responded to the items.[38] This content validation builds on Delphi methodology used to develop the Kenya-specific scale.[8]

1.4 Face validity

Face validity, also part of item development, is assessed at each site (Table 1, 1.4). First, the survey is translated from English into the language(s) of implementation and then back-translated. Then, enumerators, the predominance of whom are recruited from the target population, pre-test surveys with one another to ensure that questions are appropriate to the setting, the concept of water insecurity is understood, and translations are consistent with local dialects, i.e. that they are linguistically and culturally appropriate translations.[29]

Face validity is further ensured when site leads debrief enumerators after each day of data collection and record all the details as project field notes. The debrief is centered on experiences in the community, survey questions that are difficult to administer, and any other problems encountered. At the end of data collection for the site, enumerators engage in a final debrief, and in some cases, use a semi-structured survey that pulls the same information from across the entire site. Site leads are also orally interviewed at the end of study activities by members of the HWISE RCN about their experiences with project implementation, perceptions of questions by

For peer review only - http://bmjopen.bmj.com/site/about/guidelines.xhtml

enumerators and participants, and any additional topics that should be included or excluded in the final survey. These debriefing interviews with site leads provide additional feedback to iteratively improve training and item refinement.

Phase 2: Scale development

2.1 Data collection

Sites

Cross-sectional surveys were initially planned for six sites that would leverage investigators' active research: Bangladesh, Brazil, Guatemala, Kenya, Nepal, and Tajikistan, i.e. they were selected out of convenience. Subsequently, 22 more were added because additional sites would allow us to test the instrument across more heterogeneous cultural and geographic settings, permit an iterative analysis of the instrument (cf. "Mid-study evaluation"), and make a number of statistical analyses possible (Table 2).[39] These additional 22 sites were added by soliciting professional networks across academic institutions as well as non-governmental and governmental organizations using convenience sampling. In selecting sites, we sought maximal heterogeneity in region of the world, infrastructure (e.g. urban and rural, formal and informal settlements), and problems with water (e.g. flooding, drought, chronic scarcity, intermittent supplies). We also considered cost and feasibility of timely implementation.

For peer review only

Table 2. Characteristics of HWISE sites for scale development, by region.

World Bank Region	Site, (HWISE survey version)	Primary sources of drinking water, %	Predominant climate, precipitation at site (Köppen climate type)[1]	GNI per capita (USD)[2]	National income classification[3]	Urbanicity of site
Africa	Accra, Ghana (1.0)	Bagged/sachet water, 86.0<br>Borehole/tubewell, 5.7<br>Other, 8.3	Equatorial, winter dry (AW)	1380	Lower middle income	Urban
	Lagos, Nigeria 1.0)	Bagged/sachet water, 48.9<br>Borehole/tubewell, 34.7<br>Other, 16.4	Equatorial, winter dry (Aw)	2450	Lower middle income	Urban
	Kahemba, DRC (1.0)	Surface water, 99.7<br>Other, 0.3	Equatorial, winter dry (Aw)	420	Low income	Rural
	Bahir Dar, Ethiopia (1.0)	Unprotected dug well, 25.1<br>Rainwater collection, 20.9<br>Standpipe, 13.5<br>Surface water, 13.5<br>Protected dug well, 12.4<br>Unprotected spring, 10.0	Warm temperature, winter dry, warm summer (Cwb)	660	Low income	Rural

		Other, 4.6				
	Singida, Tanzania (1.0)	Standpipe, 48.6<br>Unprotected dug well, 17.4<br>Borehole/tubewell, 12.9<br>Other, 12.8<br>Unprotected spring, 8.3	Equatorial, winter dry (Aw)	900	Low income	Rural
	Lilongwe, Malawi (1.0)	Standpipe, 45.4<br>Piped water, 42.1<br>Other, 12.5	Warm temperature, winter dry, hot summer (Cwa)	320	Low income	Peri-urban
	Arua, Uganda (1.0)	Protected dug well, 64.8<br>Unprotected spring, 19.6<br>Other, 15.6	Equatorial, winter dry (Aw)	660	Low income	Rural
	Kisumu, Kenya (1.0)	Surface water, 17.4<br>Borehole/tubewell, 16.2<br>Rainwater, 13.8<br>Piped water, 11.3<br>Standpipe, 10.9<br>Protected dug well, 10.1<br>Unprotected dug well, 7.7<br>Unprotected spring, 6.1<br>Other, 6.5	Equatorial, fully humid (Af)	1380	Lower middle income	Rural
	Kampala, Uganda (1.0)	Standpipe, 68.3<br>Other, 21.1<br>Unprotected dug well, 10.6	Equatorial, winter dry (Aw)	660	Low income	Urban
	Morogoro, Tanzania (2.0)	Standpipe, 70.7<br>Other, 29.3	Equatorial, winter dry (Aw)	900	Low income	Rural and peri-urban
**East Asia and Pacific**	Upolu, Samoa (1.0)	Piped water, 81.4<br>Standpipe, 12.5<br>Other, 6.1	n.d.	4100	Upper middle income	Unknown
	Labuan Bajo, Indonesia (2.0)	Bagged/sachet water, 36.9<br>Protected spring, 12.9<br>Piped water, 10.0<br>Tanker truck, 9.7<br>Standpipe, 9.3<br>Protected dug well, 6.5<br>Borehole/tubewell, 5.7<br>Other, 9.0	Equatorial, fully humid (Af)	3400	Lower middle income	Urban
**Europe & Central Asia**	Dushanbe, Tajikistan (1.0)	Piped water, 58.2<br>Standpipe, 24.0<br>Tanker truck, 9.3<br>Other, 8.5	Warm temperature, summer dry, warm summer (Csb)	1110	Lower middle income	Urban
**Latin America and the Caribbean**	Ceará, Brazil (1.0)	Piped water, 59.5<br>Protected dug well, 33.9<br>Bottled water, 5.5<br>Other, 1.1	Equatorial, summer dry (As)	8840	Upper middle income	Urban
	Mérida, Mexico (1.0)	Bagged/sachet water, 50.0<br>Other, 33.6<br>Piped water, 14.4<br>Other, 2.0	Arid steppe, hot arid (BSh)	9040	Upper middle income	Urban
	Acatenango, Guatemala (1.0)	Piped water, 38.4<br>Standpipe, 31.3<br>Tanker truck, 16.2<br>Other, 14.1	Warm temperature, winter dry, warm summer (Cwb)	3790	Lower middle income	Peri-urban
	Honda, Colombia (1.0)	Piped water, 74.5<br>Standpipe, 20.4<br>Other, 5.1	Equatorial, fully humid (Af)	6320	Upper middle income	Peri-urban
	Torreón, Mexico (2.0)	Bottled water, 70.2<br>Piped water, 27.0<br>Other, 2.8	Arid steppe, hot arid (BSh)	9040	Upper middle income	Urban
	San Borja, Bolivia (2.0)	Standpipe, 41.6<br>Tanker truck, 19.3<br>Other, 10.1<br>Borehole/tubewell, 8.0<br>Piped water, 7.6<br>Rainwater collection, 6.7<br>Bottled water, 6.7	Equatorial, monsoonal (Am)	3070	Lower middle income	Rural
	Chiquimula, Guatemala (2.0)	Piped water, 65.0<br>Unprotected spring, 15.3<br>Standpipe, 12.7<br>Other, 7.0	Equatorial, monsoonal (Am)	3790	Lower middle income	Rural

For peer review only - http://bmjopen.bmj.com/site/about/guidelines.xhtml

	Gressier & Léogâne, Haiti (2.0)	Standpipe, 26.8<br>Small water vendor, 14.1<br>Bagged/sachet water, 13.1<br>Other, 10.9<br>Bottled water, 10.7<br>Borehole/tubewell, 9.3<br>Protected dug well, 7.9<br>Tanker truck, 7.2	Equatorial, winter dry (Aw)	780	Low income	Rural
	Cartagena, Colombia (2.0)	Piped water, 46.2<br>Standpipe, 34.6<br>Other, 12.4<br>Small water vendor, 6.8	Equatorial, winter dry (Aw)	6320	Upper middle income	Urban
**Middle East and North Africa**	Beirut, Lebanon (2.0)	Small water vendor, 54.5<br>Bottled water, 39.7<br>Other, 5.8	Warm temperature, summer dry, hot summer (Csa)	7680	Upper middle income	Urban
	Sistan & Balochistan, Iran (2.0)	Small water vendor, 48.0<br>Other, 30.1<br>Piped water, 21.9	Arid, desert, hot Arid (BWh)	5470	Upper middle income	Urban and peri-urban
**South Asia**	Kathmandu, Nepal (1.0)	Bottled water, 49.8<br>Piped water, 31.2<br>Tanker truck, 10.7<br>Other, 8.3	Warm temperature, winter dry, hot summer (Cwa)	730	Low income	Urban
	Pune, India (1.0)	Piped water, 89.4<br>Other, 10.6	Equatorial, winter dry (Aw)	1680	Lower middle income	Urban
	Punjab, Pakistan (2.0)	Standpipe, 26.6<br>Borehole/tubewell, 23.2<br>Piped water, 15.9<br>Rainwater collection, 14.2<br>Small water vendor, 10.3	Arid, desert, hot arid (BWh)	1510	Lower middle income	Rural
	Rajasthan, India (2.0)	Tanker truck, 55.2<br>Borehole/tubewell, 26.2<br>Other, 13.4<br>Piped water, 5.2	Arid steppe, hot arid (BSh)	1680	Lower middle income	Urban

[1]Köppen climate classification predicted using Scenario A1F1 for 2001-2025, projected to December 31, 2020 used for reference point (ESRI, ArcGIS)
[2]Gross National Income in USD from World Bank classification, data from 2017
[3]Income Classification from World Bank, data from 2017

Participant selection

To participate, individuals must be 16 or 18 years of age or older (depending on age ofconsent in each site), identify themselves to the interviewer as the person who is most knowledgeable about water acquisition and use within their households, and consent to participate. Participants are not remunerated for participation in the survey.

The target sample size at each site is 250 individuals. We consider this sample size as the minimum needed for assessing the magnitude of correlation between the observed variables and associated factor(s), and obtaining a sample pattern that is stable and approximates the population pattern.[40] If sites cannot achieve the target sample size, variation of estimated statistics will be reviewed to determine if the data can be included in the final validation of the scale.

For peer review only

For peer review only - http://bmjopen.bmj.com/site/about/guidelines.xhtml

The preferred sampling strategy for the study is random sampling of mutually exclusive and exhaustive categories of participants in areas of known high, moderate, and low water insecurity. In standalone HWISE surveys, participant selection follows a simple randomized or cluster-randomized sampling strategy (Table 3). In several sites, however, the HWISE survey is administered as part of a larger ongoing project with a predetermined survey design (e.g. in Singida, Tanzania: NCT02761876; Kahemba, Democratic Republic of Congo: NCT03157336), such that simple random sampling is not possible.

For peer review only

For peer review only - http://bmjopen.bmj.com/site/about/guidelines.xhtml

Table 3. Overview of data collection activities at each HWISE study site.

World Bank Region	Site	HWISE Survey version	Implementing partners	Month(s) and year of data collection	Final sample size	Female respondents, %	Season of data collection	Language(s) of data collection	Sampling strategy	Data collection method (software)	Details of larger study; supplementary data collected	IRB of record	Cognitive interviewing (Y/N)
Africa	Accra, Ghana	1.0	University of Miami, Delaware State University, Ghana Water Company, Northwestern University	June 2017	229	78.2	Rainy season	English	Simple random	Tablet (ODK)	Standalone	University of Miami; Delaware University, reliant on Northwestern University & Ghana Water Company	Yes
	Lagos, Nigeria	1.0	College of Medicine at the University of Lagos & Northwestern University	June-August 2017	239	73.5	Rainy season	English, Yoruba, Pidgin	Multi-stage random	Paper, entered into database (Enketo)	Standalone; adolescent menstrual hygiene	Northwestern University & University of Lagos	Yes
	Kahemba, DRC	1.0	Oregon Health Sciences University, Michigan State University, Institut National de Recherche Biomedicale	June-September 2017	392	65.6	Dry season	Kikongo, Lingala	Cluster randomized control trial	Paper/tablet hybrid (ODK)	NCT03157336: Toxicodietary and genetic determinants of susceptibility to neurodegeneration	Oregon Health Sciences University & Ministry of Health, DRC	No
	Bahir Dar, Ethiopia	1.0	Oregon State University, Emory University, Emory Ethiopia	July-August 2017	259	100	Rainy season	Amharic	Stratified random	Tablet (KoboToolbox)	NCT03075436: The Impact of Enhanced, Demand-side Sanitation and Hygiene Promotion on Sustained Behavior Change and Health in Ethiopia	Amhara Regional Health Bureau, Emory University; Oregon State University, reliant on Northwestern University	No
	Singida, Tanzania	1.0	Cornell University & Northwestern University	July-August 2017	1006	56.7	Dry season	Swahili	Purposive, community-led	Tablet (ODK)	NCT02761876: Singida Nutrition and Agroecology Project	Cornell University	No
	Lilongwe, Malawi	1.0	Georgia State University	July 2017	302	86.8	Neither rainy nor dry season	Chichewa, English	Cluster random	Tablet (ODK)	Standalone	Georgia State University	No
	Kisumu, Kenya	1.0	Pamoja Community Based Organization & Northwestern University	July 2017	247	81.3	Neither rainy nor dry season	Luo, Swahili, English	Simple random	Tablet (ODK)	Standalone; moringa	Northwestern University & African Medical Research Foundation (AMREF)	Yes
	Arua, Uganda	1.0	Michigan State University	August-September 2017	250	85.6	Rainy season	Lugbara, English	Cluster random	Paper, entered into database (Enketo)	Standalone	Michigan State University, reliant on	No

For peer review only - http://bmjopen.bmj.com/site/about/guidelines.xhtml

												Northwestern	
	Kampala, Uganda	1.0	T-Group Kampala, University of Amsterdam, Makerere University	August 2017	246	69.1	Dry season	Luganda, English	Purposive	Paper, entered into database (Enketo)	Standalone	Northwestern University & T-Group Kampala	No
	Morogoro, Tanzania	2.0	Workman Consulting & Northwestern University	March-May 2018	300	78.3	Dry season	Swahili	Cluster random	Paper, entered into database (Enketo)	Standalone; water for sanitation and hygiene (WASH)	Northwestern University & Sokoine University of Agriculture	No
**East Asia and Pacific**	Upolu, Samoa	1.0	Yale University	April 2018-Present	176^	63.4^	TBD	Samoan	Purposive community sample	Tablet (REDCap)	NIH R01HL093093: Integrated cellular, mouse and human research on a novel missense variant influencing adiposity in Samoans	Yale University	No
	Labuan Bajo, Indonesia	2.0	University of the West of England	May 2018	279	44.8	n.d.	Indonesian	Cluster random	Tablet (ODK)	Standalone	Exempt	No
**Europe & Central Asia**	Dushanbe, Tajikistan	1.0	Arizona State University & M-Vector	July-August 2017	225	73.3	Dry season	Tajik, Russian	Communities purposively selected, every 3rd residence sampled for freestanding buildings, every 5th residence within apartment buildings	Tablet (CSPro)	Global Ethnohydrology Study—ASU	Arizona State University	Yes
**Latin America and the Caribbean**	Pecem, Brazil	1.0	Texas A&M University	March 2017-February 2018	255	70.2	Neither rainy nor dry season	Portuguese	Cluster random	Paper, entered into database (Enketo)	NSF1560962: Urban water provisioning systems and household water security	Texas A&M University	No
	Mérida, Mexico	1.0	Michigan State University	July-August 2017	251	63.4	Dry season	Spanish	Cluster random	Paper, entered into database (Enketo)	Standalone	Michigan State University & Northwestern University	No
	Honda, Colombia	1.0	Pontificia Universidad Javeriana & Northwestern University	August 2017	252	63.5	Dry season	Spanish	Cluster random	Tablet (ODK)	Standalone	Northwestern University & Pontificia Universidad Javeriana	No
	Acatenango, Guatemala	1.0	Arizona State University	September-October 2017	101	93.0	Rainy season	Spanish	Cluster random	Paper, entered into database (Excel)	Global Ethnohydrology Study—ASU	Arizona State University	No
	San Borja, Bolivia	2.0	Pennsylvania State University	November-December	247	58.6	Middle/end of dry	Spanish	Simple random	Paper, entered into database	Standalone	Northwestern University	No

For peer review only - http://bmjopen.bmj.com/site/about/guidelines.xhtml

				2017			season			(Excel)			
	Chiquimula, Guatemala	2.0	McGill University & Action Against Hunger-Guatemala	January-February 2018	314	86.6	Dry season	Spanish	Single-stage systematic sampling	Tablet (ODK)	Standalone	Action Against Hunger-Guatemala & Northwestern University	No
	Gressier, Haiti	2.0	University of Florida	February-March 2018	292	98.6	Middle/end of dry season	Creole	Cluster random	Paper, entered into database (Excel)	Standalone; perceived water quality and disease	University of Florida	No
	Torreón, Mexico	2.0	Texas A&M University	April 2018	249	n.d.	Dry season	Spanish	n.d.	Paper, entered into database (Excel)	Standalone	Texas A&M University	No
	Cartagena, Colombia	2.0	University of Miami	July 2018	266	69.2	Dry season	Spanish	Simple random	Paper, entered into database (SPSS)	Standalone	University of Miami	No
**Middle East and North Africa**	Beirut, Lebanon	2.0	American University of Beirut	December 2017-January 2018	574	63.8	Rainy season	Arabic	Cluster random	Tablet (ODK)	Standalone	Northwestern University & American University of Beirut	Yes
	Sistan & Balochistan, Iran	2.0	Shahid Beheshti University of Medical Sciences	January-February 2018	306	99.0	Dry season	Farsi	Cluster random	Paper, entered into database (SPSS)	Standalone; cash transfers, demography, health center access	Exempt	No
**South Asia**	Kathmandu, Nepal	1.0	Arizona State University & Environmental and Public Health Organization (ENPHO)	June 2017	263	71.5	Rainy season	Nepali	Cluster random	Paper, entered into database(ODK)	Global Ethnohydrology Study—ASU	Arizona State University	Yes
	Punjab, Pakistan	2.0	University of Washington	February-March 2018	235	57.5	Dry season	Seraikee, Urdu	Cluster random	Paper, entered into database (Excel)	Standalone; socio-cultural practices, infant feeding practices, maternal and child malnutrition	Exempt	No
	Pune, India	1.0	Cornell University, Johns Hopkins University, BJ Government Medical College	February-Present	180^	100^	TBD	Marathi, Hindi	Parallel assignment, non-randomized	Paper, entered into database (Excel)	NIH R01HD081929: PRACHITi (Pregnancy associated changes in TB immunology); NIH K23AI129854: Effect of pregnancy and HIV on the development of tuberculosis	Cornell University, Pune IRB, Johns Hopkins University	No
	Rajasthan, India	2.0	Anode Governance Lab	March 2018	248	27.0	n.d.	Hindi	Cluster random	Paper, entered into database (Excel)	Standalone	Exempt	No

^Data collection ongoing, values based on data available as of August 2018; abbreviations used: ODK (open data kit), TBD (to be determined), n.d. (no data)

For peer review only - http://bmjopen.bmj.com/site/about/guidelines.xhtml

Sites with simple randomized sampling employ a random-walk sampling method.[41] With the simple randomized sampling strategy, a random number generator (e.g. dice or random number generating application) with set parameters (i.e. less than 20, less than 30, etc.) determines which households are surveyed (and, if needed, the direction of the random walk). Surveys are administered to each household corresponding to the random number, such that a random draw of the number 3 indicates that every third household should be sampled. Site investigators employing a cluster-randomized sampling strategy first map regions using a grid or satellite imagery (e.g. Google Maps) to identify population density based on the number of habitable structures. Clusters are selected from this grid, and households within clusters are randomly sampled in proportion to structure or population density using a random number generator, similar to the simple randomized sampling. Cluster-randomization is preferred, but simple random sampling is used when cluster data are not available, typically in sparsely populated settings.

Participant Involvement

Although formative work drew on ethnographic work that included participant involvement and the idea to develop this scale came from experiences with participants in Kenya[1], no participants were involved in developing the actual protocol. Participant involvement (e.g. cognitive interviewing; Table 1, 1.3) began with refinement of survey items once the initial list was created. Participants were not involved in developing plans for the design or implementation of the study, nor will any participants be involved in the interpretation of results or write-up of the manuscript. There are no plans to disseminate the final scale to study participants, as identifiable data were not collected in most sites. The final scale and other findings will be made available via open access publication, and be publicized through public relations and media outreach at our respective institutions.

For peer review only - http://bmjopen.bmj.com/site/about/guidelines.xhtml

Training

An HWISE training manual was developed to provide guidance on implementation.[42] This manual outlines preferred sampling strategy, minimum sample size, instructions for collecting data and choosing unique participant identification numbers, and detailed information explaining the rationale and supplementary information for each HWISE item and survey section. This manual has been subsequently translated from English into Arabic and was also adapted for use in Uganda.

Each site has at least one formally-appointed lead investigator responsible for consistent training, sampling, recruitment, and data collection. In each site, 5-10 enumerators with survey implementation experience, knowledge of the area and context, and fluency in the local language(s) are recruited. Enumerators at all sites attend a 1-2-day training. The first portion of the training curriculum is didactic and follows the survey manual. The rest of the training is interactive and tactile, with enumerators piloting the survey with one another and troubleshooting any issues that arise. After the initial training, the site lead and/or study coordinator accompany enumerators during data collection and provide feedback until enumerators are sufficiently comfortable with the survey to administer it with minimal guidance.

Data collection and management

After consent, enumerators conduct interviews with the person who identifies themselves to the enumerator as being the most knowledgeable about water acquisition and use in her or his household. In addition to the water insecurity experience items described above (HWISE 1.0 or 2.0), data are collected on sociodemographic characteristics; water acquisition, use, and storage; household food insecurity (using the Household Food Insecurity Access Scale[28]); perceived stress (using a modified, 4-item perceived stress scale[43]); and data quality (Supplemental Material 2: HWISE 2.0 survey). These additional data will be used to validate the scale and explore other

For peer review only

water insecurity phenomena in a cross-cultural manner.[44] Each interview lasts approximately 45 minutes, and we expect data collection to last approximately 10-14 days in each standalone survey site (Table 3).

Implementation of HWISE data collection began in March 2017 and is expected to end in late 2018. Data collection with HWISE 1.0 began in March 2017 and is ongoing (Table 3, currently n=4,817). Data collection using HWISE 2.0 began in November 2017 and is also ongoing (currently n=3,310).

Data are collected using both paper and tablet-based collection platforms, i.e. Open Data Kit (ODK), opendatakit.org;[45] CSPro, csprousers.org; KOBO Toolbox (Cambridge, MA), kobotoolbox.org. To reduce data collection errors, tablet-based platforms are programmed to include permissible ranges of responses, skips for questions that are not applicable, and survey items in the language(s) most common to each study site. Most responses from paper surveys are entered by enumerators, study coordinators, data managers, and/or site PIs into an online data collection platform (Enketo, enketo.org). Microsoft Excel is used when reliable internet access is unavailable.

Data are uploaded to a secure centralized aggregate server (Google App Engine). Stata14 (StataCorp, College Station, TX, USA) is used for data cleaning following a data cleaning protocol agreed upon by the HWISE RCN (Supplemental Material 3: Data cleaning protocol).

Implementation fidelity

To ensure implementation fidelity, enumerators are debriefed daily following data collection. Both enumerators and site PIs are debriefed post-implementation (Supplemental Material 4: Interviewer debriefing guide). Further, each survey contains a module on perceived data quality (e.g. explanation of missing data, distractions, and issues with recruitment) that is filled in by the enumerator immediately post-interview.

For peer review only - http://bmjopen.bmj.com/site/about/guidelines.xhtml

Analytic strategy

Three software packages will be used for analyses: Stata14 (StataCorp, College Station, TX, USA) to run basic descriptive statistics; Mplus version 8 (Muthén & Muthén, Los Angeles, CA, USA) for Classical Test Theory analysis; and WINSTEPS (Winsteps®, Beaverton, OR, USA) for Item Response Theory (Rasch) analysis.

Scale development (Table 1, 2.1-2.4) and evaluation (Table 1, 3.1-3.3) will be informed by analyses corresponding to two scaling theories: Classical Test Theory[46], implemented by factor analysis, and Item Response Theory[47], using Rasch models.

2.2 Item reduction

First, items with large cumulative missing cases (>30%), i.e. “don’t know,” “non-applicable,” or true missing responses, will be dropped (Table 1, 2.2). This will help to eliminate items that are not understood or are not widely applicable, and therefore do not reflect cross-cultural experiences of water insecurity.

Thereafter, items will be further dropped based on low correlation coefficients. In Classical Test Theory, we will identify items with low (<0.30) inter-item and item-total correlation coefficients across the multiple sites in this study.[8,48]

Within the Rasch paradigm, we will identify and remove items that misfit the models by assessing infit and outfit.[49,50] Condition item independence (i.e. items conditional on the scale that are not correlated) will be assessed using residual correlation metrics. Items will be dropped if residual correlation is >0.2.[51]

2.3 Identify factor structure

Factor analysis with data from multiple sites will be used to identify the optimal latent structure (Table 1, 2.3). We will examine this structure site-by-site, comparing factor structures, magnitudes of factor loadings, eigenvalues for sample correlation matrices, and global model

For peer review only - http://bmjopen.bmj.com/site/about/guidelines.xhtml

fitness statistics. Items with low factor loadings (<0.30), split factor loadings, and high residual variances (>0.50) will be considered for deletion.[34]

2.4 Assess measurement equivalence

Measurement equivalence concerns the extent to which the psychometric properties of the observed indicators are generalizable across groups or over time.[52–55] It holds "when a test measures a construct in the same way regardless of group membership and is violated when individuals from different groups respond to the test in a dissimilar manner".[39] A violation of equivalence implies our inability to make comparisons about the measurement and meaning of scale values across groups (e.g. sites, cultures, languages).[39] To determine measurement equivalence across sites in HWISE 1.0 and 2.0, we will conduct confirmatory factor analysis using multi-group confirmatory factor analysis and alignment optimization.[56–58]

Phase 3: Scale evaluation

3.1 Score scale items Once a water insecurity scale that is equivalent across sites is provisionally identified, we will use scale scores in both weighted forms (factor scores) and unweighted forms (sum scores) to assess the external validity of our scale.

3.2 Reliability

To test for the reliability (internal consistency) of the items, we will estimate Cronbach's alpha for the aggregated data and each site.[59] The Rasch reliability statistic is analogous to Cronbach's alpha. In our analyses, we will consider reliability to be ideal if it is greater than 0.90.[51]

3.3 Validity

We will then examine 3 types of validity: predictive, convergent, and discriminant validity. Predictive validity is "the extent to which a measure predicts the answers to some other question or a result which it ought to be related with".[60] Using both linear regression and

For peer review only - http://bmjopen.bmj.com/site/about/guidelines.xhtml

structural equation models, we will test for predictive validity by regressing scores of food insecurity, perceived stress, and income on HWISE scores.

Convergent validity is the “degree to which scores on a studied instrument are related to measures of other constructs that can be expected on theoretical grounds and accumulated knowledge to be close to the one tapped into by this instrument”.[48] To test for convergent validity, we will assess the relationships between HWISE Scale scores and individual items that have shown to be closely related to the concept of water insecurity. Specifically, we will use correlations and linear regression to examine the strength of the relationships between the sum scores of HWISE Scale and time to water source, number of trips to water source, and amount of money spent purchasing water. Larger correlation and regression coefficients and smaller standard deviations of residuals will be indicative of support for convergent validity.

Discriminant validity is the “degree to which scores on a studied instrument are differentiated from behavioral manifestations of other constructs”.[48] Using correlations and linear regression, we will examine the strength of the relationships between the water insecurity scale scores and total amount of water stored (in liters). Non-significant correlation and regression coefficients or weak associations will be indicative of support for discriminant validity.

A test of differentiation between a range of “known groups” will be conducted using the Student’s *t*-test and analysis of variance;[34,48] these groups will be based on accumulated knowledge. We will determine the distribution of household water insecurity scores across known groups, including primary source of drinking water (improved vs. unimproved sources), water treatment (treated vs. untreated), gender of household head (male vs. female), and injuries associated with water acquisition (yes vs. no).[8,20,21,24,61] Under the Rasch measurement model,

For peer review only - http://bmjopen.bmj.com/site/about/guidelines.xhtml

differentiating between known groups will also be conducted using differential item functioning. We will determine whether each scale item performs differently in each of the subgroups. Differential item functioning is attained when the probabilities of an item being endorsed is unequal for the two subgroups.[8,62]

In sum, selection of the set of items to be included in the final scale will be based on several criteria. The criteria for inclusion of an item are: reliable in each site, fits theoretically and empirically with concepts related to water insecurity, has face and content validity in each site, shows equivalent measurement and meaning across sites, and contributes to predictive, convergent, and discriminant validity in each site.[63] We anticipate that not every item will meet each criterion perfectly, and judgment about tradeoffs of which items to include will be required. These judgments will be made considering the additional criteria of having a diversity of items in the final scale that cover as many facets of water insecurity as reasonably possible. We anticipate that the final scale will have fewer than 20 items, which will reduce the likelihood of participant fatigue and make its widespread application more feasible.

Mid-study evaluations

In August 2017, five months after data collection began in 8 of 16 HWISE 1.0 sites, HWISE RCN members met at Northwestern University to review and discuss data received to date and thematically sort HWISE items. This led to the reduction and refinement of HWISE for the second wave of survey implementation (HWISE 2.0), which was ultimately administered across 12 sites (Tables 2 & 3). In February 2018, HWISE RCN members involved in scale validation met at McGill University to review HWISE 2.0 responses to date and further refine the HWISE module. Members of the analytic team also hold regular conference calls to review subsequent results and complete the scale validation process.

For peer review only - http://bmjopen.bmj.com/site/about/guidelines.xhtml

ETHICS AND DISSEMINATION

All participants were verbally consented by enumerators in their language of choice using a standardized script (Supplemental Material 2: HWISE 2.0 survey). Study activities are reviewed and approved by all appropriate ethical review boards (Table 3).

The final HWISE Scale will be presented at scientific, programmatic, and policy venues. The survey will be made available through open-access publication. Data will eventually be made available upon request from the HWISE RCN. Associated findings will be disseminated to scientists, public health professionals, and policy makers through peer-reviewed journals and conference presentations. Measures to quantify household food insecurity have been transformative for policy, research, and humanitarian aid efforts globally, and we expect that an analogous measure for water insecurity will achieve similar impacts.[30–32]

ACKNOWLEDGEMENTS

We are very grateful to our participants, without whom this scale would not be possible. We would also like to warmly and sincerely thank the field teams for their hard work and dedication to this project: Velly Emina, Victoria Yesufu, Annah Adakhilan, Adekunbi Adejokun, Adebari Adewunmi, Damola Adelakun, Nike Odunaike, Anthony Sekoni, Ramon Babamole, Kayode Badru (Lagos, Nigeria); Prashant Rimal, Sarita Lawaju, Roshna Twanabasu, Renuka Baidhya, Ayaswori Byanju, Menuka Prajapati, Ranju Magar (Kathmandu, Nepal); Andrew Mvula, Wisdom Mwale, Faith Kanyika, Wyson Samata, Fanney Kanyenda, Mcdonad Mpangwe (Lilongwe, Malawi); Maxwell Akosah-Kusi, David Okai Nunoo, Rita Antanah, Michael Nyoagbe (Accra, Ghana); Gulnoza Sharipova, Ganjina Hudoieva, Gulsara Nozirova, Shahobiddin Murodov, Navrasta Shoeva, Nasiba Gadoeva, Markhabo Ibragimova, Vahidova Saodat (Dushanbe, Tajikistan); Milton Marin Morales (San Borja, Bolivia); Wicklife Odhiambo Orero, Judith Atieno Owuour, Philip Otieno Orude, Sylvia Achieng Odhiambo, Kennedy Oduor (Kisumu, Kenya); Daniel Guerrero, Daniela Avila, Kelly Johana Diaz Ceballos, Valentina Giraldo Bohorquez, Pedro Castillo (Honda, Colombia); Moses Mwebaza, Dorren Bamanya, Alines Mpandu, Alex Kazooza, John Ssemwogerer, Olivia Nakamya, Kimbugwe Muhammed, Simon Kyagera, Gerald Ssozi, Solomon Wakida, Matteo Andrea Corsini, Ann Apio, Atim Catu, Nahwera Julie (Kampala, Uganda); Alonzi Francis, Candia Alex, Alesi Christine, Adjonye Doreen, Aputru Florence, Ayakaka Beatrice (Arua, Uganda); Michel Lupamba, Mary Aziza Mulumba, Smith Tshibulenu, Kevis Kamanda, Thérèse Hosa (Kahemba, Democratic Republic of Congo); Eliwaza Mpeko, James Raphael, Emmanuel Katabi, Raziki Amon, Theresia Ononga, Eliofoo Yohana, Neilu Issack, Faudhia Kitiku, Janeth Kacholi, Nyambuli Deus, Oliver

For peer review only

Mwanjati, Faith Titus, Fadhali Nyasiro, Mwantum Mkama (Singida, Tanzania); Robinson Bernier, Berlyne Bien-Aimé, Claude Civil (Gressier & Léogâne, Haiti); Luambano Kihoma, Generoza Amos, Patricia Msolla, Peter Amandus, Cyril Lissu, Fredy Bernard, Joylight Mbitta, Raphael Chelele, Alan Kimbita, Elizabeth Msiuike (Morogoro, Tanzania); Daniel Eduardo Lemaitre, Saray Noel Tarra, Luis Murillo Ortega, Natalia Yepes Montes, Jairo Andres Aviles Rojano, Juan Jose de la Espriella Correa, Marcela Florez, Juan Andres Barrios, Stephanie Escobar Diaz, Yuriza Martinez, Carlos Anibal Batista Ruiz (Cartagena, Colombia). Further, we are grateful to Northwestern University Information Technology's Research Computing Services team, especially Frank Elavsky for his creation of Figure 1.

FUNDING

We gratefully acknowledge our funders: the Competitive Research Grants to Develop Innovative Methods and Metrics for Agriculture and Nutrition Actions (IMMANA). IMMANA is funded with UK Aid from the UK government. This project was also supported by the Buffett Institute for Global Studies and the Center for Water Research at Northwestern University; Arizona State University's Center for Global Health at the School of Human Evolution and Social Change and Decision Center for a Desert City (National Science Foundation SES-1462086); the Office of the Vice Provost for Research of the University of Miami; the National Institutes of Health grant NIEHS/FIC R01ES019841 for the Kahemba Study, DRC. Sera Young was supported by the National Institutes of Health (NIMH R21 MH108444; NIMH K01 MH098902). Wendy Jepson was supported by the National Science Foundation (BCS-1560962).

AUTHOR CONTRIBUTIONS

SLY conceptualized the study, developed HWISE items, wrote the manuscript, obtained funding, and oversaw data collection and analysis. SMC helped develop HWISE items, wrote the manuscript, and managed data. GB developed the data analysis and validation plan, and helped write the analytic section of the manuscript. TBN assisted with study design and supported scale analysis and validation. ZJ proposed data analysis and helped write the analytic section of the manuscript. JDM developed tools for data collection and managed data. AB proposed analyses for item development. EAF and HMQ proposed data analysis. WEJ developed HWISE items. RCS supported development of HWISE items and assisted with preparation of the manual. JS proposed analyses for item development. AW developed HWISE items and proposed analyses for item development. HWISE RCN members provided substantial contributions to data acquisition and interpretation. All authors critically reviewed and approved the final draft of the manuscript.

CONFLICTS OF INTEREST

None declared

DATA STATEMENT

Data are currently being collected and are not yet available for access.

For peer review only

REFERENCES

1. Jepson WE, Wutich A, Collins SM, Boateng GO, Young SL. Progress in household water insecurity metrics: a cross-disciplinary approach. WIREs Water. 2017 Apr 11;4(3):e1214-21.

2. Geere J-AL, Hunter PR, Jagals P. Domestic water carrying and its implications for health: a review and mixed methods pilot study in Limpopo Province, South Africa. Environmental Health. 2010 Jan 1;9(1):52–13.

3. Prüss-Ustün A, Bartram J, Clasen T, Colford JM, Cumming O, Curtis V, et al. Burden of disease from inadequate water, sanitation and hygiene in low- and middle-income settings: a retrospective analysis of data from 145 countries. Tropical Medicine & International Health. 2014 Aug;19(8):894–905.

4. Sorenson SB, Morssink C, Campos PA. Safe access to safe water in low income countries: Water fetching in current times. Social Science & Medicine. 2011 May;72(9):1522–6.

5. Mason LR. Examining Relationships between Household Resources and Water Security in an Urban Philippine Community. Journal of the Society for Social Work and Research. 2014 Dec 1;5(4):489–512.

6. Wutich A, Brewis A. Food, Water, and Scarcity: Toward a Broader Anthropology of Resource Insecurity. Current Anthropology. 2014 Aug;55(4):444–68.

7. Cook C, Bakker K. Water security: debating an emerging paradigm. Global Environmental Change. 2012;22(1):94–102.

8. Boateng GO, Collins SM, Mbullo P, Wekesa P, Onono M, Neilands TB, et al. A novel household water insecurity scale: Procedures and psychometric analysis among postpartum women in western Kenya. Gnambs T, editor. PLOS ONE. 2018 Jun 8;13(6):e0198591.

9. Workman CL, Ureksoy H. Water insecurity in a syndemic context: Understanding the psycho-emotional stress of water insecurity in Lesotho, Africa. Social Science & Medicine. 2017 Apr;179:52–60.

10. Weiser SD, Young SL, Cohen CR, Kushel MB, Tsai AC, Tien PC, et al. Conceptual framework for understanding the bidirectional links between food insecurity and HIV/AIDS. Am J Clin Nutr. 2011 Dec;94(6):1729S-1739S.

11. Mekonnen MM, Hoekstra AY. Four billion people facing severe water scarcity. Science Advances. 2016 Feb 12;2(2):e1500323–e1500323.

12. Hirabayashi Y, Mahendran R, Koirala S, Konoshima L, Yamazaki D, Watanabe S, et al. Global flood risk under climate change. Nature Climate Change. 2013 Sep;3(9):816–21.

13. Bain R, Cronk R, Hossain R, Bonjour S, Onda K, Wright J, et al. Global assessment of exposure to faecal contamination through drinking water based on a systematic review. Tropical Medicine & International Health. 2014 Aug;19(8):917–27.

For peer review only

14. Falkenmark M, Lundqvist J, Widstrand C. Macro-scale water scarcity requires micro-scale approaches. Aspects of vulnerability in semi-arid development. Nat Resour Forum. 1989 Nov;13(4):258–67.

15. Rijsberman FR. Water scarcity: Fact or fiction? Agricultural Water Management. 2006;80(1–3):5–22.

16. Sullivan CA, Meigh JR, et al. The Water Poverty Index: Development and application at the community scale. Natural Resources Forum. 2003;189–99.

17. Global Water Partnership. Proceedings from the GWP workshop: Assessing Water Security with Appropriate Indicators [Internet]. Stockholm; 2014 p. 120. Available from: http://www.gwp.org/Global/ToolBox/Publications/P763_GWP_Proceedings_Paper.pdf

18. de França Doria M. Factors influencing public perception of drinking water quality. Water Policy. 2010 Feb;12(1):1–19.

19. Grey D, Sadoff CW. Sink or Swim? Water security for growth and development. Water Policy. 2007 Dec;9(6):545–71.

20. Wutich A, Ragsdale K. Water insecurity and emotional distress: coping with supply, access, and seasonal variability of water in a Bolivian squatter settlement. Social science & medicine (1982). 2008;67(12):2116–25.

21. Stevenson EGJ, Greene LE, Maes KC, Ambelu A, Tesfaye YA, Rheingans R, et al. Water insecurity in 3 dimensions: an anthropological perspective on water and women's psychosocial distress in Ethiopia. Soc Sci Med. 2012;75(2):392–400.

22. Bartram J, Lewis K, Lenton R, Wright A. Focusing on improved water and sanitation for health. The Lancet. 2005 Feb;365(9461):810–2.

23. Jepson W. Measuring no-win waterscapes: Experience-based scales and classification approaches to assess household water security in colonias on the US--Mexico border. Geoforum. 2014;51(C):107–20.

24. Tsai AC, Kakuhikire B, Mushavi R, Vo echovska D, Perkins JM, McDonough AQ, et al. Population-based study of intra-household gender differences in water insecurity: reliability and validity of a survey instrument for use in rural Uganda. Journal of Water and Health. 2016 Apr 22;14(2):280–92.

25. UN General Assembly. Transforming our world: the 2030 Agenda for Sustainable Development [Internet]. 2015 Oct. Report No.: a/RES/70/1. Available from: https://sustainabledevelopment.un.org/content/documents/21252030%20Agenda%20for%20Sustainable%20Development%20web.pdf

26. High Level Panel on Water. Making Every Drop Count: An Agenda for Water Action [Internet]. 2018 Mar. Available from: https://sustainabledevelopment.un.org/content/documents/17825HLPW_Outcome.pdf

For peer review only - http://bmjopen.bmj.com/site/about/guidelines.xhtml

27. Pérez-Escamilla R, Melgar-Quiñonez H, Nord M, Alvarez Uribe MC, Segall-Correa A. Escala Latinoamericana y Caribeña de Seguridad Alimentaria (ELCSA) [Latinamerican and Caribbean Food Security Scale]. Perspectivas en Nutrición Humana. 2007;117–34.

28. Coates J, Swindale A, Bilinsky P. Household food insecurity access scale (HFIAS) for measurement of food access: Indicator guide. Washington DC: Food and Nutrition Technical Assistance Project; 2007.

29. Ballard T, Kepple AW, Cafiero C. The Food Insecurity Experience Scale. Rome, Italy: Food & Agriculture Organization; 2013.

30. Cafiero C, Viviani S, Nord M. Food security measurement in a global context: The food insecurity experience scale. Measurement. 2018 Feb;116:146–52.

31. Pérez-Escamilla R. Can experience-based household food security scales help improve food security governance? Global Food Security. 2012 Dec;1(2):120–5.

32. Pérez-Escamilla R, Guberta MB, Rogers BL, Hromi-Fiedler A. Food security measurement and governance: Assessment of the usefulness of diverse food insecurity indicators for policy makers. Global Food Security. 2017 Sep;14:96–104.

33. Haynes SN, Richard DC, Kubany ES. Content Validity in Psychological Assessment: A Functional Approach to Concepts and Methods. Psychological Assessment. 1995;7(3):238–47.

34. Boateng GO, Neilands TB, Frongillo EA, Melgar-Quiñonez H, Young SL. Best Practices for Developing and Validating Scales for Public Health and Nutrition Research: A Primer. Frontiers in Public Health. 2018 Jun 11;1–18.

35. Krumdieck NR, Collins SM, Wekesa P, Mbullo P, Boateng GO, Onono M, et al. Household water insecurity is associated with a range of negative consequences among pregnant Kenyan women of mixed HIV status. Journal of Water and Health. 2016 Dec 1;14(6):1028–31.

36. McCoach DB, Gable RK, Madura JP. Defining, Measuring, and Scaling Affective Constructs. In: Instrument Development in the Affective Domain [Internet]. New York, NY: Springer New York; 2013 [cited 2018 Mar 23]. p. 33–90. Available from: http://link.springer.com/10.1007/978-1-4614-7135-6_2

37. Webb P, Iskandarani M. Water insecurity and the poor: issues and research needs. 1998 [cited 2016 Apr 20]; Available from: http://www.iatp.org/files/Water_Insecurity_and_the_Poor_Issues_and_Resea.htm

38. Ericsson KA, Simon HA. Verbal Reports as Data. Psychological Review. 1980;87(3):215–51.

For peer review only

For peer review only - http://bmjopen.bmj.com/site/about/guidelines.xhtml

578 39. Kim ES, Cao C, Wang Y, Nguyen DT. Measurement Invariance Testing with Many
579 Groups: A Comparison of Five Approaches. Structural Equation Modeling: A
580 Multidisciplinary Journal. 2017 Jul 4;24(4):524–44.

581 40. Guadagnoli E, Velicer WF. Relation of sample size to the stability of component patterns.
582 Psychological Bulletin. 1988 Mar;103(2):265–75.

583 41. Designing Household Survey Samples: Practical Guidelines [Internet]. Geneva,
584 Switzerland: United Nations Statistics Division; 2005. Available from:
585 https://unstats.un.org/unsd/demographic/sources/surveys/Handbook23June05.pdf

586 42. Collins SM, Young SL. The Household Water InSecurity Experiences (HWISE) Scale
587 Study Manual [Internet]. 2018. Available from: https://doi.org/10.21985/N2M10F

588 43. Cohen S, Kamarck T, Mermelstein R. A global measure of perceived stress. J Health Soc
589 Behav. 1983;24(4):385–96.

590 44. Brewis A, Rosinger A, Wutich A, Adams E, Cronk L, Pearson A, et al. Household Water
591 Sharing Favors Charitable Transfers to Neighbors: Evidence from Eight Water-Insecure
592 Sub-Saharan African Sites. Under review.

593 45. Brunette W, Sundt M, Dell N, Chaudhri R, Breit N, Borriello G. Open data kit 2.0:
594 expanding and refining information services for developing regions. In: Proceedings of the
595 14th Workshop on Mobile Computing Systems and Applications. ACM; 2013. p. 10.

596 46. Cappelleri JC, Jason Lundy J, Hays RD. Overview of Classical Test Theory and Item
597 Response Theory for the Quantitative Assessment of Items in Developing Patient-Reported
598 Outcomes Measures. Clinical Therapeutics. 2014 May;36(5):648–62.

599 47. Harvey RJ, Hammer AL. Item Response Theory. The Counseling Psychologist. 1999
600 May;27(3):353–83.

601 48. Raykov T, Marcoulides GA. Introduction to psychometric theory. New York, NY:
602 Routledge; 2011. 335 p.

603 49. Wright BD, Stone M. Best Test Design: Rasch Measurement. Chicago, IL: Mesa Press;
604 1979.

605 50. Smith EV. Evidence for the reliability of measures and validity of measure interpretation: a
606 Rasch measurement perspective. J Appl Meas. 2001;2(3):281–311.

607 51. Linacre JM, Wright BD. A user's guide to BIGSTEPS: Rasch-model computer program.
608 Chicago: Mesa Press; 1993.

609 52. Byrne B, Watkins D. The issue of measurement invariance revisited. Journal of cross-
610 cultural psychology. 2003;34(2):155–75.

For peer review only

For peer review only - http://bmjopen.bmj.com/site/about/guidelines.xhtml

53. Harkness JA, Vijver FJR van de, Mohler PP, editors. Cross-cultural survey methods. Hoboken, N.J: J. Wiley; 2003.

54. Leroy JL, Ruel M, Frongillo EA, Harris J, Ballard TJ. Measuring the Food Access Dimension of Food Security: A Critical Review and Mapping of Indicators. Food and Nutrition Bulletin. 2015 Jun;36(2):167–95.

55. Sideridis GD, Tsaousis I, Al-harbi KA. Multi-Population Invariance With Dichotomous Measures: Combining Multi-Group and MIMIC Methodologies in Evaluating the General Aptitude Test in the Arabic Language. Journal of Psychoeducational Assessment. 2015 Sep;33(6):568–84.

56. Asparouhov T, Muthén B. Multiple-Group Factor Analysis Alignment. Structural Equation Modeling: A Multidisciplinary Journal. 2014 Oct 2;21(4):495–508.

57. Kim ES, Yoon M. Testing Measurement Invariance: A Comparison of Multiple-Group Categorical CFA and IRT. Structural Equation Modeling: A Multidisciplinary Journal. 2011 Apr 6;18(2):212–28.

58. Lomazzi V. Using Alignment Optimization to Test the Measurement Invariance of Gender Role Attitudes in 59 Countries. methods, data, analyses, Vol 12, No 1 (2018) [Internet]. 2018 Jan 31 [cited 2018 Aug 15]; Available from: http://mda.gesis.org/index.php/mda/article/view/2017.09

59. Cronbach LJ. Coefficient alpha and the internal structure of tests. Psychometrika. 1951 Sep;16(3):297–334.

60. Fowler FJ. Improving survey questions: design and evaluation. Thousand Oaks: Sage Publications; 1995.

61. Hadley C, Freeman MC. Assessing reliability, change after intervention, and performance of a water insecurity scale in rural Ethiopia. Food Security. 2016 Jan 1;8(4):855–64.

62. Angoff W. Use of difficulty and discrimination indices for detecting item bias. In: Handbook of Methods for Detecting Test Bias. Baltimore, MD: Johns Hopkins University Press; 1982. p. 96–116.

63. Gregorich SE. Do self-report instruments allow meaningful comparisons across diverse population groups? Testing measurement invariance using the confirmatory factor analysis framework. Medical care. 2006;44(11 Suppl 3):S78.

For peer review only - http://bmjopen.bmj.com/site/about/guidelines.xhtml

647 Table 1. Overview of planned methods and analyses for the development of the HWISE Scale. Adapted from [34].
649 Table 2. Characteristics of HWISE sites for scale development, by region.
650 Table 3. Overview of data collection activities at each HWISE study site.

652 Figure 1. Map of HWISE study sites. Image credit: Frank Elavsky, Northwestern University Information Technology, Research Computing Services.

655 Supplemental Material 1. Overview of HWISE survey items, by implementation phase
656 Supplemental Material 2. HWISE 2.0 survey and consent form
657 Supplemental Material 3. HWISE data cleaning protocol
658 Supplemental Material 4. Interviewer debriefing guide

For peer review only

For peer review only - http://bmjopen.bmj.com/site/about/guidelines.xhtml

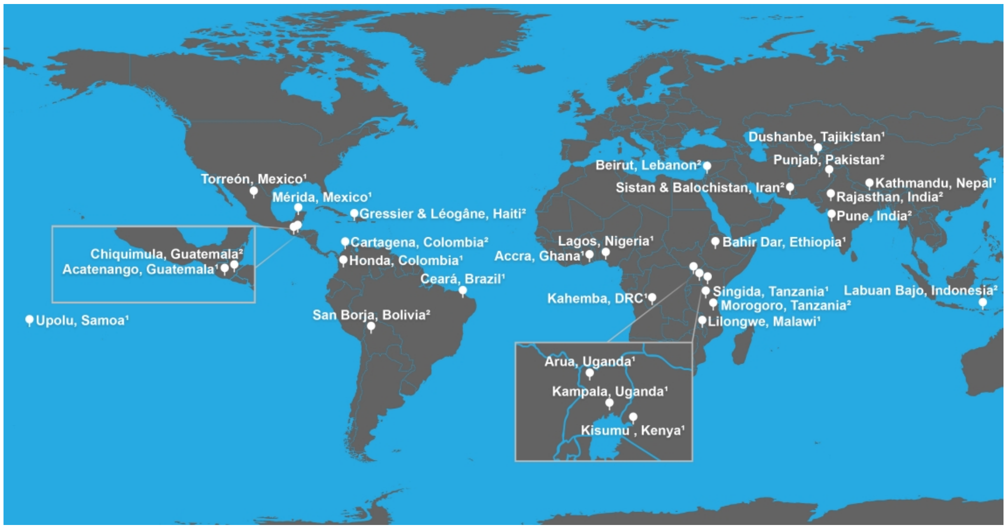

Figure 1. Map of HWISE study sites. Image credit: Frank Elavsky, Northwestern University Information Technology, Research Computing Services.

1322x687mm (72 x 72 DPI)

Supplemental Material 1. Overview of HWISE survey items, by implementation phase.

	HWISE 1.0	HWISE 2.0	Rationale for Modifications to HWISE 1.0
**Response Options:**	Never (0), Rarely (1), Sometimes (2), Often (3), Always (4)	Never (0), Rarely (1), Sometimes (2), Often (3), Always (4)	
**Number of Questions:**	32	30	
**Dimensions:**	Multidimensional: psychological, disease, nutrition, economic, social	Multidimensional: psychological, disease, nutrition, economic, social, cultural	
**Domains**	**HWISE 1.0**	**HWISE 2.0**	
**PSYCHOLOGICAL**	In the last 4 weeks, how frequently did you or anyone in your household **worry** you would not have **enough water for all of** your household needs?	In the last 4 weeks, how frequently did you or anyone in your household **worry** you would not have enough water for all of your household needs?	—
	In the last 4 weeks, how frequently have you or anyone in your household **worried about the safety** of the person getting water for your household?	In the last 4 weeks, how frequently have you or anyone in your household **worried about the safety of the person** getting water for your household?	—
	In the last 4 weeks, how frequently did you or anyone in your household **feel upset** about your water situation?	In the last 4 weeks, how frequently did you or anyone in your household feel **angry** about your water situation?	Word choice changed since upset does not translate well in many contexts and is tied to religiosity.
	In the last 4 weeks, how frequently has your or anyone in your household's **day been interrupted by your water situation**, including getting or distributing water within the household?	In the last 4 weeks, how frequently has you or anyone in your household had to **change schedules/plans** due to problems with your water situation, such as problems getting or distributing water within the household? Activities that may have been interrupted include caring for others, doing household chores, etc.	HWISE 1.0 items related to schedules subsumed into this question. Rephrased from "interrupted" to "changed schedules" because the former confused many participants in cognitive interviews.
		How **satisfied** are you with your water situation on a scale of 1-5? (1 is not at all satisfied and 5 is completely satisfied).	Starting with a question framed in the negative may bias people towards over-exaggerating their difficulties with water.
**SOCIAL**	In the last 4 weeks, how frequently have problems with water prevented you or anyone in your household from **attending social events** (i.e. church, funerals, community gatherings, etc.)?	In the last 4 weeks, how frequently have problems with water prevented you or anyone in your household from **attending social or cultural events** (e.g. church, funerals, community gatherings, cultural practices, etc.)?	Revised to include cultural components of water.
	In the last 4 weeks, how frequently did the **children** in your household **miss school** because they were getting water?	In the last 4 weeks, how frequently did the children in your household **miss school** or **go to school late** because of problems with water (e.g. time spent fetching water, lack of water for bathing, etc.)?	In many contexts, children only miss part of the school day as a consequence of water problems (e.g. time spent fetching water, cleanliness). analyses.
	In the last 4 weeks, how frequently did you or anyone in your household have problems with water that caused **difficulties with neighbors** or others in the community?	In the last 4 weeks, how frequently did you or anyone in your household have problems with water that caused **difficulties with neighbors, water providers, or others** in the community?	In many contexts, individuals face difficulties with their water providers.
	In the last 4 weeks, how frequently did you or anyone in your household have problems with water that caused **difficulties within your household**?	In the last 4 weeks, how frequently did you or anyone in your household have problems with water that caused **difficulties within your household**?	—
		In the last 4 weeks, how frequently have you or anyone in your household been unable to access the **water that you preferred**?	Previous version did not captures the concept of accessing a dignified, culturally-preferred water source.
		In the last 4 weeks, how frequently have problems with water caused you or anyone in your household to **feel ashamed**/excluded/stigmatized?	There are many uses and dimensions of water; currently, we were previously lacking a specific cultural component.
**ECONOMIC**	In the last 4 weeks, how frequently has the **time spent getting** water prevented you or anyone in your household from **earning money** (e.g. engaging in paid work, economic activities)?	In the last 4 weeks, how frequently have problems with water prevented you or anyone in your household from **earning money** (e.g. engaging in paid work, economic activities)?	Changed language to be applicable for flood and drought conditions.
	In the last 4 weeks, how frequently have you or anyone in your household **lacked money needed** to buy water?	In the last 4 weeks, how frequently have you or anyone in your household **lacked money** needed to **buy water**?	—
	In the last 4 weeks, how frequently have you or anyone in your household **thought of leaving** [*name of town*] because there was no water there?	In the last 4 weeks, how frequently have you or anyone in your household thought of **moving dwellings** because of the water situation there?	Language deemed too extreme; people tend not to leave town since they have family nearby but *are* likely to switch households. Also, rephrased to say water situation instead of no water, e.g. in case of flooding or contaminated water.
	In the last 4 weeks, how frequently did you or anyone in your household want to **buy water but there was nowhere to buy it from**?	In the last 4 weeks, how frequently did you or anyone in your household want to buy water but there was **nowhere to buy it from**?	—
	In the last 4 weeks, how frequently have you or anyone in your household asked to **borrow water** from other people?	In the last 4 weeks, how frequently have you or anyone in your household asked to **borrow** water from other people?	—
	In the last 4 weeks, how frequently have you or anyone in your household **loaned water** to anyone?	In the last 4 weeks, how frequently have you or anyone in your household **loaned water** to anyone? **Only asked in a subset of HWISE 2.0 sites*	—
**CARE/HEALTH**	In the last 4 weeks, how frequently has the **time spent getting** water prevented you or anyone in your household from **caring for children in the household**?		Subsumed under "change schedules/plans" in HWISE 2.0.
	In the last 4 weeks, how frequently has the **time spent getting** water prevented you or anyone in your household from **doing household chores** (such as cooking, preparing food, washing clothes, etc.)?		Subsumed under "change schedules/plans" in HWISE 2.0.
	In the last 4 weeks, how frequently has there **not been enough** water in the household to **wash clothes**?	In the last 4 weeks, how frequently has there not been enough water in the household to **wash clothes**?	—
	In the last 4 weeks, how frequently have you or anyone in your household had to go **without washing hands** after **dirty activities** (e.g., defecating or changing diapers, cleaning animal dung) because you didn't have enough water?	In the last 4 weeks, how frequently have you or anyone in your household had to go **without washing hands** after **dirty activities** (e.g., defecating or changing diapers, cleaning animal dung) because of problems with water?	Changed language to be applicable for flood and drought conditions.
	In the last 4 weeks, how frequently have you or anyone in your household not had enough water to **wash the faces and hands of children in your household?**	In the last 4 weeks, how frequently have you or anyone in your household not **washed the faces and hands of children** because of problems with water?	Changed language to account for flood events.
	In the last 4 weeks, how frequently have you or anyone in your household had to go without **washing their body** because there wasn't enough water?	In the last 4 weeks, how frequently have you or anyone in your household had to go without **washing their body** because of problems with water (e.g. not enough water, dirty, unsafe)?	Changed language to be applicable for flood and drought conditions.
	In the last 4 weeks, how frequently did you or anyone in your household want to **treat your water**, but couldn't? By treat, I mean boiling, using chemicals to treat, or other ways you make your water safe to use or drink.		Not many people experienced this.
	In the last 4 weeks, how frequently have you or anyone in your household **drank water** that **tasted bad**?	In the last 4 weeks, how frequently have you or anyone in your household drank water that **looked, tasted, and/or smelled bad**?	Participants mentioned that water can taste, look, and smell bad; the question is more encompassing by including all of these organoleptic properties.
	In the last 4 weeks, how frequently have you or anyone in your household **actually drank water** that you thought was unsafe?	In the last 4 weeks, how frequently have you or anyone in your household drank water that you thought was **unsafe**?	"Actually" does not add anything to the question, therefore removed.
	In the last 4 weeks, how frequently have you or anyone in your household **not had enough water to take medications**?		Not enough people experienced this; too specific.
	In the last 4 weeks, how frequently have you or anyone in your household not gotten water where you wanted to because you were **too sick or weak** to get water?		Too rare.
**NUTRITIONAL**	In the last 4 weeks, how frequently have you or anyone in your household had to **change what was being eaten** because there wasn't enough water (e.g. for washing foods, cooking, etc.)?	In the last 4 weeks, how frequently have you or anyone in your household had to **change what was being eaten** because there were problems with water (e.g. for washing foods, cooking, etc.)?	Changed language to be applicable for flood and drought conditions.
	In the last 4 weeks, how frequently has there not been as much water to drink as you would like for you or anyone in your household?	In the last 4 weeks, how frequently has there not been **as much water to drink** as you would like for you or anyone in your household?	—
	In the last 4 weeks, how frequently have you or anyone in your household gone to **sleep thirsty**?	In the last 4 weeks, how frequently have you or anyone in your household gone to **sleep thirsty** because there wasn't any water to drink?	Ensures that the question is measuring lack of water.
	In the last 4 weeks, how frequently has there been **no water whatsoever** in your household?	In the last 4 weeks, how frequently has there been **no useable or drinkable water** whatsoever in your household?	Changed language to be applicable for flood and drought conditions.
**AGRI-CULTURAL**	In the last 4 weeks, how frequently has your household **not had enough** water for your **garden, crops, or fruit trees**?	In the last 4 weeks, how frequently has your household water situation impacted the cultivation of your **garden, crops, or fruit trees**?	Changed language to be applicable for flood and drought conditions.
	In the last 4 weeks, how frequently has your household **not had enough water** to give to your **animals and poultry?**	In the last 4 weeks, how frequently has your household water situation impacted your raising of **animals and poultry**?	Changed language to be applicable for flood and drought conditions.
**OTHER**	In the last 4 weeks, how frequently has your household **water supply** from your main water source **been interrupted?**	In the last 4 weeks, how frequently has your household water supply from your main water source been **interrupted** or **limited** (e.g. water pressure, less water than expected)?	Include "limited" to cover instances where water is not available in normal or anticipated quantities.

HWISE 2.0 Consent Script, Survey, and Sources

Household
Water
Insecurity
Experiences

Version 25, June 13, 2018

For peer review only - http://bmjopen.bmj.com/site/about/guidelines.xhtml

Household Water Insecurity Experiences

HWISE Consent Script

You are being asked to take part in a research study about the different factors affecting water insecurity and health for people in your area. We are asking you to take part because you live in this area and have unique insight into how water is acquired and used in your community.

What this study is about: The purpose of this study is to learn about the various ways people acquire and use water and any consequences that arise from household water insecurity. From this study, we will be able to develop a scale to measure household water insecurity in many settings across the world.

What we will ask you to do: If you agree to be in this study, we will ask you to participate in a brief survey during which we will ask you questions about the composition of your household, water acquisition and use, food insecurity, and stress.

Risks and benefits: There is the risk that you may find some of the questions about your household to be sensitive. There are no immediate benefits to you, but the information learned in this study will be useful for researchers who are studying household water insecurity. The information we learn through this survey may be shared with researchers at other institutions, but your personal identifying information will not be shared or linked to your responses.

Compensation. There is no compensation for taking part in this study.

Your answers will be confidential. We will write or record your responses to this survey using tablets, your name and information will not be included in any part of the survey. The written records and any information you share will be kept private. In any sort of report we make public we will not include any information that will make it possible to identify you. Research records will be kept in a locked file or in secure, password-protected online storage; only researchers will have access to the records.

Taking part is voluntary. Taking part in this study is completely voluntary. You may skip any questions that you do not want to answer. If you decide not to take part or to skip some of the questions, it will not affect your future relationship with Arizona State University or Northwestern University. If you decide to take part, you are free to withdraw at any time.

If you have questions: The lead researcher conducting this study is Dr. Sera Young at Northwestern University. If you have questions later, you can contact Dr. Young at sera.young@northwestern.edu. If you have any questions or concerns regarding your rights as a subject in this study, you may contact the Northwestern University Institutional Review Board (IRB) at +1 (847) 467-1723 or http://www.northwestern.edu.

Now I would like to ask if you agree to participate in this study.
Do you agree to participate in this study? **YES/NO**
Do you agree to allow me to record your responses to questions? **YES/NO**

Cross-cultural study of household water insecurity	
Interviewer____	Date__________________
Time at start of interview____________________	Time at end of interview____________________
Country ___________________	Region/District__________________
Neighborhood__________________	
Participant ID:_______________	Language of interview: _________________
Participant gender: (0) Male (1) Female	
Participant place of residence: (0) Rural (2) Peri-urban (3) Urban	
Participant ethnicity:	
Participant ID should start with 1st two letters of country, eg. BA, NE, GU, GH…	

1. Screening Questions		
**Code Name**	**Questions**	**Coding Classification**
SQ1a	Do you agree to participate in this survey?	0…No<br>1…Yes
SQ2	Are you 16 years of age or older?	0…No<br>1…Yes
SQ3	Would you consider yourself knowledgeable about water acquisition and use within your household?	0…No<br>1…Yes
SQ4	If no, who is most knowledgeable about water acquisition and use within your household? Who should I talk to?	

2. Socio Demographic Questions		
SD1	Role in household: What is your relationship to the head of your household? By household, I mean all people who sleep under the same roof and take food from the same pot.	1….Self<br>2….Spouse/partner<br>3….Adult child<br>4….Other
SD2	What is the gender of household head?	0…Male<br>1…Female
SD9	What is your current relationship status?	1…Single/separated or divorced<br>2…Widowed<br>3…Have a partner who lives separately<br>4…Have a partner who lives with you<br>5…Married
SD3	How old are you?	
SD4	Who is primarily responsible for making sure there is enough water in the house? By household, I mean all people who sleep under the same roof and take food from the same pot.<br><br>*If primary responsibility is shared, choose (5) and then also circle the two categories of people who share the responsibility.*	1…Self<br>2…Spouse/partner<br>3…Children<br>4…Other family members<br>5…Shared responsibility

For peer review only - http://bmjopen.bmj.com/site/about/guidelines.xhtml

SD5	How many children (≤16 years) live in your household? By household, I mean all people who sleep under the same roof and take food from the same pot.	
SD6	How many adults (>16 years) including yourself live in your household? By household, I mean all people who sleep under the same roof and take food from the same pot.	
SD7	What type of housing do you live in?	1….House/condominium (owned)<br>2….House/condominium (rented)<br>3….Apartment (owned)<br>4…..Apartment (rented)<br>5….Farm (own)<br>6…..Farm (lease)<br>7…..Informal settlement/squatter community<br>8……Refugee/internally displaced person camp<br>9…..Other ____________________
SD8	What is your religion?	1….Christianity<br>2….Judaism<br>3….Islam<br>4…..Hinduism<br>5…..Buddhism<br>6…..Nonreligious<br>7…...Other
SD8a	What is your denomination?	

**3. Household Water Insecurity Experiences Scale (32 questions)**<br>*Now I'm going to ask you about your own personal experiences with water. For each of the items, please indicate how many times within the past 4 weeks or 30 days. Interviewer, please mark the response that best corresponds with the coding classification. For example, if participants says 15 times, you need to mark/circle code 4.*		
**Code name**	**Question**	**Coding Classification**
HWISE1	How **satisfied** are you with your water situation on a scale of 1-5? (1 is not at all satisfied and 5 is completely satisfied).	1…Not at all satisfied<br>2<br>3<br>4<br>5...Completely satisfied
HWISE2	In the last 4 weeks, how frequently have you or anyone in your household been unable to access the **water that you preferred**?	1...Never (0 times in the last 4 weeks)<br>2...Rarely (1–2 times in the last 4 weeks)<br>3...Sometimes (3–10 times in the last 4 weeks)<br>4...Often (11-20 times in the last 4 weeks)<br>5….Always (More than 20 times in the last 4 weeks)

For peer review only - http://bmjopen.bmj.com/site/about/guidelines.xhtml

		99….Don't know<br>88…Not applicable/I don't have this
HWISE3	In the last 4 weeks, how frequently did you or anyone in your household **worry** you would not have enough water for all of your household needs?	1...Never (0 times in the last 4 weeks)<br>2...Rarely (1–2 times in the last 4 weeks)<br>3...Sometimes (3–10 times in the last 4 weeks)<br>4...Often (11-20 times in the last 4 weeks)<br>5….Always (More than 20 times in the last 4 weeks)<br>99….Don't know<br>88…Not applicable/I don't have this
HWISE4	In the last 4 weeks, how frequently have you or anyone in your household **worried about the safety of the person** getting water for your household? *By getting, I mean: traveling to, collecting the water, and returning with the water.*	1...Never (0 times in the last 4 weeks)<br>2...Rarely (1–2 times in the last 4 weeks)<br>3...Sometimes (3–10 times in the last 4 weeks)<br>4...Often (11-20 times in the last 4 weeks)<br>5….Always (More than 20 times in the last 4 weeks)<br>99….Don't know<br>88…Not applicable/I don't have this
HWISE5	In the last 4 weeks, how frequently has your household water supply from your main water source been **interrupted** or **limited** (e.g. water pressure, less water than expected)?	1...Never (0 times in the last 4 weeks)<br>2...Rarely (1–2 times in the last 4 weeks)<br>3...Sometimes (3–10 times in the last 4 weeks)<br>4...Often (11-20 times in the last 4 weeks)<br>5….Always (More than 20 times in the last 4 weeks)<br>99….Don't know<br>88…Not applicable/I don't have this
HWISE5a	If your supply was interrupted or limited, were these **expected** (announced/scheduled) or unexpected?	0…Unexpected<br>1…Announced/Scheduled
HWISE6	In the last 4 weeks, how frequently has your household water situation impacted the cultivation of your **garden, crops, or fruit trees**?	1...Never (0 times in the last 4 weeks)<br>2...Rarely (1–2 times in the last 4 weeks)<br>3...Sometimes (3–10 times in the last 4 weeks)<br>4...Often (11-20 times in the last 4 weeks)<br>5….Always (More than 20 times in the last 4 weeks)<br>99….Don't know<br>88…Not applicable/I don't have this
HWISE7	In the last 4 weeks, how frequently has your household water situation impacted your raising of **animals and poultry**?	1...Never (0 times in the last 4 weeks)<br>2...Rarely (1–2 times in the last 4 weeks)<br>3...Sometimes (3–10 times in the last 4 weeks)<br>4...Often (11-20 times in the last 4 weeks)

For peer review only - http://bmjopen.bmj.com/site/about/guidelines.xhtml

		5….Always (More than 20 times in the last 4 weeks)<br>99….Don't know<br>88…Not applicable/I don't have this
HWISE8	In the last 4 weeks, how frequently have problems with water prevented you or anyone in your household from **earning money** (e.g. engaging in paid work, economic activities)?	1...Never (0 times in the last 4 weeks)<br>2...Rarely (1–2 times in the last 4 weeks)<br>3...Sometimes (3–10 times in the last 4 weeks)<br>4...Often (11-20 times in the last 4 weeks)<br>5….Always (More than 20 times in the last 4 weeks)<br>99….Don't know<br>88…Not applicable/I don't have this
HWISE9	In the last 4 weeks, how frequently have you or anyone in your household **lacked money** needed to **buy water**?	1...Never (0 times in the last 4 weeks)<br>2...Rarely (1–2 times in the last 4 weeks)<br>3...Sometimes (3–10 times in the last 4 weeks)<br>4...Often (11-20 times in the last 4 weeks)<br>5….Always (More than 20 times in the last 4 weeks)<br>99….Don't know<br>88…Not applicable/I don't have this
HWISE10	In the last 4 weeks, how frequently did you or anyone in your household want to buy water but there was **nowhere to buy it from**?	1...Never (0 times in the last 4 weeks)<br>2...Rarely (1–2 times in the last 4 weeks)<br>3...Sometimes (3–10 times in the last 4 weeks)<br>4...Often (11-20 times in the last 4 weeks)<br>5….Always (More than 20 times in the last 4 weeks)<br>99….Don't know<br>88…Not applicable/I don't have this
HWISE11	In the last 4 weeks, how frequently did the children in your household **miss school** or **go to school late** because of problems with water (e.g. time spent fetching water, lack of water for bathing, etc.)?	1...Never (0 times in the last 4 weeks)<br>2...Rarely (1–2 times in the last 4 weeks)<br>3...Sometimes (3–10 times in the last 4 weeks)<br>4...Often (11-20 times in the last 4 weeks)<br>5….Always (More than 20 times in the last 4 weeks)<br>99….Don't know<br>88…Not applicable/I don't have this
HWISE12	In the last 4 weeks, how frequently has there not been enough water in the household to **wash clothes**?	1...Never (0 times in the last 4 weeks)<br>2...Rarely (1–2 times in the last 4 weeks)<br>3...Sometimes (3–10 times in the last 4 weeks)<br>4...Often (11-20 times in the last 4 weeks)<br>5….Always (More than 20 times in the last 4 weeks)<br>99….Don't know<br>88…Not applicable/I don't have this

For peer review only - http://bmjopen.bmj.com/site/about/guidelines.xhtml

HWISE13	In the last 4 weeks, how frequently have you or anyone in your household had to **change what was being eaten** because there were problems with water (e.g. for washing foods, cooking, etc.)?	1...Never (0 times in the last 4 weeks)<br>2...Rarely (1–2 times in the last 4 weeks)<br>3...Sometimes (3–10 times in the last 4 weeks)<br>4...Often (11-20 times in the last 4 weeks)<br>5….Always (More than 20 times in the last 4 weeks)<br>99….Don't know<br>88…Not applicable/I don't have this
HWISE14	In the last 4 weeks, how frequently have you or anyone in your household had to go **without washing hands** after **dirty activities** (e.g., defecating or changing diapers, cleaning animal dung) because of problems with water?	1...Never (0 times in the last 4 weeks)<br>2...Rarely (1–2 times in the last 4 weeks)<br>3...Sometimes (3–10 times in the last 4 weeks)<br>4...Often (11-20 times in the last 4 weeks)<br>5….Always (More than 20 times in the last 4 weeks)<br>99….Don't know<br>88…Not applicable/I don't have this
HWISE15	In the last 4 weeks, how frequently have you or anyone in your household not **washed the faces and hands of children** because of problems with water?	1...Never (0 times in the last 4 weeks)<br>2...Rarely (1–2 times in the last 4 weeks)<br>3...Sometimes (3–10 times in the last 4 weeks)<br>4...Often (11-20 times in the last 4 weeks)<br>5….Always (More than 20 times in the last 4 weeks)<br>99….Don't know<br>88…Not applicable/I don't have this
HWISE16	In the last 4 weeks, how frequently have you or anyone in your household had to go without **washing their body** because of problems with water (e.g. not enough water, dirty, unsafe)?	1...Never (0 times in the last 4 weeks)<br>2...Rarely (1–2 times in the last 4 weeks)<br>3...Sometimes (3–10 times in the last 4 weeks)<br>4...Often (11-20 times in the last 4 weeks)<br>5….Always (More than 20 times in the last 4 weeks)<br>99….Don't know<br>88…Not applicable/I don't have this
HWISE17	In the last 4 weeks, how frequently has you or anyone in your household had to **change schedules/plans** due to problems with your water situation, such as problems getting or distributing water within the household? Activities that may have been interrupted include caring for others, doing household chores, etc.	1...Never (0 times in the last 4 weeks)<br>2...Rarely (1–2 times in the last 4 weeks)<br>3...Sometimes (3–10 times in the last 4 weeks)<br>4...Often (11-20 times in the last 4 weeks)<br>5….Always (More than 20 times in the last 4 weeks)<br>99….Don't know<br>88…Not applicable/I don't have this
HWISE18	In the last 4 weeks, how frequently have problems with water prevented you or anyone in your household from **attending social or cultural events**	1...Never (0 times in the last 4 weeks)<br>2...Rarely (1–2 times in the last 4 weeks)<br>3...Sometimes (3–10 times in the last 4 weeks)<br>4...Often (11-20 times in the last 4 weeks)

For peer review only - http://bmjopen.bmj.com/site/about/guidelines.xhtml

	(e.g. church, funerals, community gatherings, cultural practices, etc.)?	5….Always (More than 20 times in the last 4 weeks)<br>99….Don't know<br>88…Not applicable/I don't have this
HWISE19	In the last 4 weeks, how frequently have you or anyone in your household drank water that **looked, tasted, and/or smelled bad**?	1...Never (0 times in the last 4 weeks)<br>2...Rarely (1–2 times in the last 4 weeks)<br>3...Sometimes (3–10 times in the last 4 weeks)<br>4...Often (11-20 times in the last 4 weeks)<br>5….Always (More than 20 times in the last 4 weeks)<br>99….Don't know<br>88…Not applicable/I don't have this
HWISE20	In the last 4 weeks, how frequently have you or anyone in your household drank water that you thought was **unsafe**?	1...Never (0 times in the last 4 weeks)<br>2...Rarely (1–2 times in the last 4 weeks)<br>3...Sometimes (3–10 times in the last 4 weeks)<br>4...Often (11-20 times in the last 4 weeks)<br>5….Always (More than 20 times in the last 4 weeks)<br>99….Don't know<br>88…Not applicable/I don't have this
HWISE21c	If you needed to borrow water, from how many people could you borrow water?	
HWISE21	In the last 4 weeks, how frequently have you or anyone in your household asked to **borrow** water from other people?	1...Never (0 times in the last 4 weeks)<br>2...Rarely (1–2 times in the last 4 weeks)<br>3...Sometimes (3–10 times in the last 4 weeks)<br>4...Often (11-20 times in the last 4 weeks)<br>5….Always (More than 20 times in the last 4 weeks)<br>99….Don't know<br>88…Not applicable/I don't have this
HWISE21a	From whom? Please list all the ways you are connected to these people. For example, neighbor, family member, or both neighbor and family member.	Person #1:<br><br>Person #2:<br><br>Person #3:
HWISE21b	What were you expected to give in return?	
HWISE30	In the last 4 weeks, how frequently have you or anyone in your household loaned water to anyone?	1...Never (0 times in the last 4 weeks)<br>2...Rarely (1–2 times in the last 4 weeks)<br>3...Sometimes (3–10 times in the last 4 weeks)<br>4...Often (11-20 times in the last 4 weeks)<br>5….Always (More than 20 times in the last 4 weeks)

For peer review only - http://bmjopen.bmj.com/site/about/guidelines.xhtml

		99….Don't know<br>88…Not applicable/I don't have this
HWISE22	In the last 4 weeks, how frequently did you or anyone in your household have problems with water that caused **difficulties with neighbors, water providers, or others** in the community?	1...Never (0 times in the last 4 weeks)<br>2...Rarely (1–2 times in the last 4 weeks)<br>3...Sometimes (3–10 times in the last 4 weeks)<br>4...Often (11-20 times in the last 4 weeks)<br>5….Always (More than 20 times in the last 4 weeks)<br>99….Don't know<br>88…Not applicable/I don't have this
HWISE23	In the last 4 weeks, how frequently did you or anyone in your household have problems with water that caused **difficulties within your household**?	1...Never (0 times in the last 4 weeks)<br>2...Rarely (1–2 times in the last 4 weeks)<br>3...Sometimes (3–10 times in the last 4 weeks)<br>4...Often (11-20 times in the last 4 weeks)<br>5….Always (More than 20 times in the last 4 weeks)<br>99….Don't know<br>88…Not applicable/I don't have this
HWISE24	In the last 4 weeks, how frequently did you or anyone in your household feel **angry** about your water situation?	1...Never (0 times in the last 4 weeks)<br>2...Rarely (1–2 times in the last 4 weeks)<br>3...Sometimes (3–10 times in the last 4 weeks)<br>4...Often (11-20 times in the last 4 weeks)<br>5….Always (More than 20 times in the last 4 weeks)<br>99….Don't know<br>88…Not applicable/I don't have this
HWISE25	In the last 4 weeks, how frequently has there not been **as much water to drink** as you would like for you or anyone in your household?	1...Never (0 times in the last 4 weeks)<br>2...Rarely (1–2 times in the last 4 weeks)<br>3...Sometimes (3–10 times in the last 4 weeks)<br>4...Often (11-20 times in the last 4 weeks)<br>5….Always (More than 20 times in the last 4 weeks)<br>99….Don't know<br>88…Not applicable/I don't have this
HWISE26	In the last 4 weeks, how frequently have you or anyone in your household gone to **sleep thirsty** because there wasn't any water to drink?	1...Never (0 times in the last 4 weeks)<br>2...Rarely (1–2 times in the last 4 weeks)<br>3...Sometimes (3–10 times in the last 4 weeks)<br>4...Often (11-20 times in the last 4 weeks)<br>5….Always (More than 20 times in the last 4 weeks)<br>99….Don't know<br>88…Not applicable/I don't have this
HWISE27	In the last 4 weeks, how frequently has there been **no useable or drinkable water** whatsoever in your household?	1...Never (0 times in the last 4 weeks)<br>2...Rarely (1–2 times in the last 4 weeks)<br>3...Sometimes (3–10 times in the last 4 weeks)

For peer review only - http://bmjopen.bmj.com/site/about/guidelines.xhtml

		4...Often (11-20 times in the last 4 weeks)<br>5….Always (More than 20 times in the last 4 weeks)<br>99….Don't know<br>88…Not applicable/I don't have this
HWISE28	In the last 4 weeks, how frequently have you or anyone in your household thought of **moving dwellings** because of the water situation there?	1...Never (0 times in the last 4 weeks)<br>2...Rarely (1–2 times in the last 4 weeks)<br>3...Sometimes (3–10 times in the last 4 weeks)<br>4...Often (11-20 times in the last 4 weeks)<br>5….Always (More than 20 times in the last 4 weeks)<br>99….Don't know<br>88…Not applicable/I don't have this
HWISE29	In the last 4 weeks, how frequently have problems with water caused you or anyone in your household to **feel ashamed**/excluded/stigmatized?	1...Never (0 times in the last 4 weeks)<br>2...Rarely (1–2 times in the last 4 weeks)<br>3...Sometimes (3–10 times in the last 4 weeks)<br>4...Often (11-20 times in the last 4 weeks)<br>5….Always (More than 20 times in the last 4 weeks)<br>99….Don't know<br>88…Not applicable/I don't have this

**4. Water Access**<br>*Now I would like to learn how and where you acquire water.*		
Wat1	What is currently the **primary source** of drinking water for your household? (Choose only ONE)	1…Piped water<br>2…Stand pipe<br>3…Borehole/tubewell<br>4…Protected dug well<br>5…Unprotected dug well<br>6…Protected spring<br>7…Unprotected spring<br>8…Rainwater collection<br>9…Small water vendor<br>10…Tanker truck<br>11…Bottled water<br>12…Bagged/sachet water<br>13…Surface water (pond, river, lake)<br>14…Other person ____________<br>15…Other_________
Wat2	What is currently the **primary source** of non-drinking water for your household? (Choose only ONE)	1…Piped water<br>2…Stand pipe<br>3…Borehole/tubewell<br>4…Protected dug well<br>5…Unprotected dug well<br>6…Protected spring<br>7…Unprotected spring<br>8…Rainwater collection

For peer review only - http://bmjopen.bmj.com/site/about/guidelines.xhtml

		9…Small water vendor<br>10…Tanker truck<br>11…Bottled water<br>12…Bagged/sachet water<br>13…Surface water (pond, river, lake)<br>14…Other person ____________<br>15…Other_________
Wat3	How long (in minutes) does it take to go to the water source, get water and come back (including wait time)? *(If water source is in household/compound, record 00 minutes)*	____________ minutes
Wat4	How many trips in total are made to this site per week (not including household/compound?)?	__________ trips
Wat4a	Have you ever been injured while fetching water?	0...No<br>1…Yes
Wat4b	If so, how?	
Wat5	In the past 4 weeks, approximately how much money did you spend on getting water for your household?	Units: ______ Amount: ________
Wat6	In the past 4 weeks, was drinking water for your household typically treated in any way to make it safer?	0…No<br>1…Yes<br>DK…Don't know
Wat7	What is the primary way that your household treats your **drinking** water?	1…Do not treat it<br>2…Boil<br>3…Filter<br>4…Add chemicals<br>5…Other (Specify):<br>___________________________
Wat8	In the past 4 weeks, how much money did you spend to treat water (including money for chemicals to treat water)?	Currency: ______ Amount: ________

only

**5. Water Quantity, Utility and Stability**<br>*Now I would like to ask you questions on the quantity of water you have in your household and the amount you drink.*		
Wat9	What is the estimate of the current amount of drinking water (liters) stored in your household?	________ Liters
Wat10	What is the estimate of the current amount of non-drinking water (liters) stored in your household?	________ Liters
Wat11	Which of the months in the year does your household mostly experience **water shortage** (circle all months)?	1…January 7…July<br>2…February 8…August<br>3…March 9…September<br>4…April 10…October<br>5…May 11…November

For peer review only - http://bmjopen.bmj.com/site/about/guidelines.xhtml

		6…June 12…December
Wat12	Which of the months in the year does your household have **excessive amounts** of water (tick the specific months)?	1…January 7…July<br>2…February 8…August<br>3…March 9…September<br>4…April 10…October<br>5…May 11…November<br>6…June 12…December
Wat13	What times of day does your household experience water shortages?	0…None<br>1…Morning (Sunrise (6:00am) to 11:59am)<br>2…Afternoon (12:00 – 5:00pm)<br>3…Evening (5:01 – 8:00pm)<br>4…Night (8:01pm – 5:59am)
Wat14	What do you see as the main **cause of problems with water** in your area?	
Wat15	What do you do when you **don't have enough water** and don't have enough money to buy water?	

6. Food Insecurity (HFIAS)

Now I'm going to ask you about your experiences with access to food in the last four weeks. I will ask you about the frequencies that you have experienced a few situations, and I want you to tell me how frequently it has happened in the last four weeks.

Code Name	Questions	Coding Classification
FI1	In the past four weeks, how frequently did you worry that your household would not have enough food?	1…Never (0 times in the last 4 weeks)<br>2…Rarely (1 – 2 times in the last 4 weeks)<br>3…Sometimes (3 – 10 times in the last 4 weeks)<br>4…Often (More than 10 times in the last 4 weeks)<br>9…Don't know
FI2	In the past four weeks, how frequently were you or any household member not able to eat foods you preferred because you couldn't obtain them because of a lack of resources (such as money, business, land, or any other thing that you would require to help you obtain other types of food)?	1…Never (0 times in the last 4 weeks)<br>2…Rarely (1 – 2 times in the last 4 weeks)<br>3…Sometimes (3 – 10 times in the last 4 weeks)<br>4…Often (More than 10 times in the last 4 weeks)<br>9…Don't know
FI3	In the past four weeks, how frequently did you or any household member have to eat a limited variety of food due to lack of resources (such as money, business, land, or any other thing that you would require to help you obtain other types of food)?	1…Never (0 times in the last 4 weeks)<br>2…Rarely (1 – 2 times in the last 4 weeks)<br>3…Sometimes (3 – 10 times in the last 4 weeks)<br>4…Often (More than 10 times in the last 4 weeks)<br>9…Don't know
FI4	In the past four weeks, how frequently did you or any household member have to eat some foods that you really did not want to eat because of a lack of resources to obtain other types of food?	1…Never (0 times in the last 4 weeks)<br>2…Rarely (1 – 2 times in the last 4 weeks)<br>3…Sometimes (3 – 10 times in the last 4 weeks)<br>4…Often (More than 10 times in the last 4 weeks)<br>9…Don't know
FI5	In the past four weeks, how frequently did	1…Never (0 times in the last 4 weeks)

For peer review only - http://bmjopen.bmj.com/site/about/guidelines.xhtml

	you or any household member have to eat a smaller meal than you felt you needed, meaning a little amount of food that did not satisfy you, because there was not enough food?	2…Rarely (1 – 2 times in the last 4 weeks)<br>3…Sometimes (3 – 10 times in the last 4 weeks)<br>4…Often (More than 10 times in the last 4 weeks)<br>9…Don't know
FI6	How many meals do you think you should eat in a day?	
FI7	In the past four weeks, how frequently did you or anyone in your household have to eat fewer meals in a day because there was not enough food?	1…Never (0 times in the last 4 weeks)<br>2…Rarely (1 – 2 times in the last 4 weeks)<br>3…Sometimes (3 – 10 times in the last 4 weeks)<br>4…Often (More than 10 times in the last 4 weeks)<br>9…Don't know
FI8	In the past four weeks, how frequently was there ever no food to eat of any kind in your household because of lack of resources to get food?	1…Never (0 times in the last 4 weeks)<br>2…Rarely (1 – 2 times in the last 4 weeks)<br>3…Sometimes (3 – 10 times in the last 4 weeks)<br>4…Often (More than 10 times in the last 4 weeks)<br>9…Don't know
FI9	In the past four weeks, how frequently did you or any household member go to sleep at night hungry because there was not enough food?	1…Never (0 times in the last 4 weeks)<br>2…Rarely (1 – 2 times in the last 4 weeks)<br>3…Sometimes (3 – 10 times in the last 4 weeks)<br>4…Often (More than 10 times in the last 4 weeks)<br>9…Don't know
FI10	In the past four weeks, how frequently did you or any household member go hungry for a whole day and night because of limited food in the house?	1…Never (0 times in the last 4 weeks)<br>2…Rarely (1 – 2 times in the last 4 weeks)<br>3…Sometimes (3 – 10 times in the last 4 weeks)<br>4…Often (More than 10 times in the last 4 weeks)<br>9…Don't know

7. Perceived Stress Scale

The questions in this scale ask you about your feelings and thoughts during the last month. In each case, indicate how often you felt or thought a certain way.

PS1	In the past four weeks, how often have you felt that you were unable to control the important things in your life?	1…Never (0 times in the last 4 weeks)<br>2…Almost Never (1-2 times in the last 4 weeks)<br>3…Sometimes (3-10 times in the last 4 weeks)<br>4…Fairly Often (11-20 times in the last 4 weeks)<br>5…Very Often (More than 20 times in the last 4 weeks)
PS2	In the last month, how often have you felt confident about your ability to handle your personal problems?	1…Never (0 times in the last 4 weeks)<br>2…Almost Never (1-2 times in the last 4 weeks)<br>3…Sometimes (3-10 times in the last 4 weeks)<br>4…Fairly Often (11-20 times in the last 4 weeks)<br>5…Very Often (More than 20 times in the last 4 weeks)
PS3	In the last month, how often have you felt that things were going your way?	1…Never (0 times in the last 4 weeks)<br>2…Almost Never (1-2 times in the last 4 weeks)<br>3…Sometimes (3-10 times in the last 4 weeks)<br>4…Fairly Often (11-20 times in the last 4 weeks)<br>5…Very Often (More than 20 times in the last 4 weeks)
PS4	In the last month, how often have you felt difficulties were piling up so high that you could not overcome them?	1…Never (0 times in the last 4 weeks)<br>2…Almost Never (1-2 times in the last 4 weeks)<br>3…Sometimes (3-10 times in the last 4 weeks)

For peer review only - http://bmjopen.bmj.com/site/about/guidelines.xhtml

		4…Fairly Often (11-20 times in the last 4 weeks)<br>5…Very Often (More than 20 times in the last 4 weeks)

8. Infant feeding

This question will ask you about infant feeding and how it can be impacted by the water situation in your area.

BF1	Can you tell me some ways that the water situation here affects how infants (under 12 months of age) are fed? (*Interviewer, prompt for three ways.)*	1.<br>2.<br>3.

2a. Socio Demographic Questions (continued)

We are now going to ask you three final questions about your standing in your community.

SD8	What is your current occupation or how do you earn money?	
SD9	Here is a picture of a ladder. Please think of this ladder as representing the socioeconomic standing of people ***in your community.***<br><br>At the top of the ladder are those who are best off; they have the most money, the most education, the most respected jobs. At the bottom are people who have the least money, least education, and least jobs.<br><br>Where would you place yourself on this ladder? Please touch the rung/step.<br><br>Interviewer, write the corresponding number here: ________	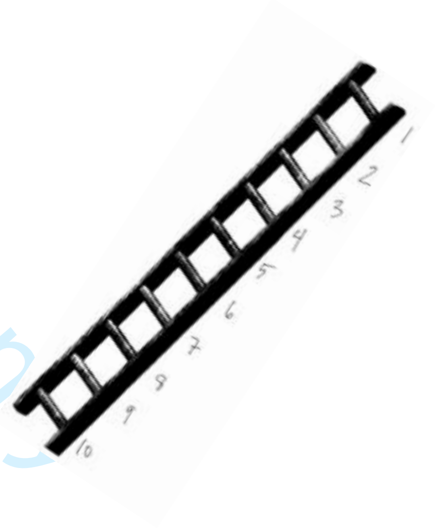

For peer review only - http://bmjopen.bmj.com/site/about/guidelines.xhtml

SD11	Here is a picture of a ladder. Please think of this ladder as representing the water situation of people ***in your community.***<br><br>At the top of the ladder are those who have the best water situation; they can easily get enough water for everything that they need and never have too much. At the bottom are people who have the most problems with water.<br><br>Where would you place yourself on this ladder? Please touch the rung/step.<br><br>Interviewer, write the corresponding number here: ________	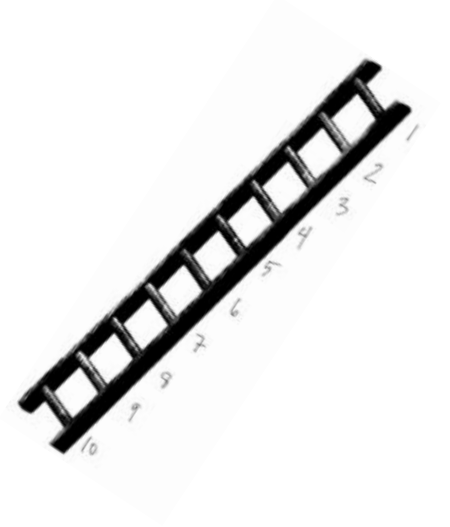
SD10	What is the primary monthly income for your household? (/month)	Units______ Amount ________

Wrap-up Question: Is there anything else you would like to share about your experiences with water for your household and how this affects your life?

Do you have any questions for us? ***Thank you*** *for participating in this survey.*

**Data Quality: For the interviewer to complete**<br>*Please give your assessment on the quality and reliability of the data you have collected in this survey.*		
DQ01	Did the respondent show any of the following? *(check all that apply):*	___ Mistrust of you or the study<br>___ Dishonesty, lying, or inventing responses that didn't seem true<br>___ Fear of you or the study<br>___ Hostility, anger or resentment<br>___ Evasion or trying to avoid answering
DQ02	Were there any interruptions or distractions?	0…No<br>1…Yes, but I don't think it influenced responses<br>2…Yes, and I think it influenced the answers
DQ03	What is your overall assessment of the quality of the data in this survey?	0…**Excellent**. The respondent understood the survey and was engaged and there were no interruptions<br>1…**Just okay**. The participant may not have understood all or part of the survey well or there were some interruptions.<br>2…**Suspect**. The participant misunderstood the survey or did not participate well or there were many interruptions.
DQ04	The quality of the data from this interview is really important to us. Please explain your answers above, in terms of how the data may have been affected.<br>Is there anything else we need to know?	

For peer review only - http://bmjopen.bmj.com/site/about/guidelines.xhtml

HWISE 2.0 Survey Sources

Topic	Brief Description	Source
**Socio-demographics**	Neighborhood, region, district/residence	Adapted from UNICEF Multiple Indicator Cluster Survey (MICS)[1]
	Role in household	
	Gender of Household head/respondent	
	Relationship status	Adapted from Demographic and Health Surveys (DHS) Household Questionnaire[2]
	Age of household head/respondent	
	Person responsible for collecting water in the household	Adapted from WHO & UNICEF Core Questions on Drinking Water and Sanitation for Household Surveys[3]
	Household size (# of adults & # of kids)	Adapted from UNICEF Multiple Indicator Cluster Survey (MICS)[1]
	Type of housing	Developed by investigators
	Religion and denomination	
**Household Water Insecurity Experiences (HWISE) Scale**	30-item household water insecurity experiences scale	Developed from extensive literature review[5] and team's knowledge of water insecurity[4,9-12]
**Water sharing**	Borrowing/loaning, what are you expected to give in return, from whom do you borrow?	Developed by investigators
**Water Quality**	Source of primary drinking water (WHO categories for improved & unimproved sources)	Adapted from WHO & UNICEF Core Questions on Drinking Water and Sanitation for Household Surveys[3]
	Source of primary non-drinking water (WHO categories for improved & unimproved sources)	
	Assessment of drinking water to be safe or unsafe	Developed by investigators
	Participants treating their water	Adapted from Demographic and Health Surveys (DHS) Household Questionnaire[2]
**Water Accessibility**	The amount of money spent by the household in water collection	Developed by investigators
	Estimate the time spent in collecting water from water source	Adapted from WHO & UNICEF Core Questions on Drinking Water and Sanitation for Household Surveys[3]
	Frequency of water collection	
**Water Quantity**	Amount of drinking water stored in household (L)	Developed by investigators
	Amount of non-drinking water stored in household (L)	
**Water Utility**	Amount of water drank in a day (L)	Developed by investigators
**Water Stability/Reliability**	Which of the months in a year do households experience water excess and scarcity?	Developed by investigators
	Which times of day do households experience water scarcity?	

For peer review only

**Food Insecurity**	9-item estimate via Household Food Insecurity Access Scale (HFIAS).	Adapted from the Household Food Insecurity Access Scale (HFIAS)[8]
**Perceived Stress**	4-item Estimate via Cohen's Perceived Stress Scale.	Adapted from the Perceived Stress Scale[7]
**Infant Feeding**	1 open-ended question on perceptions of how water insecurity may affect infant & young child feeding	Developed by investigators
**Socioeconomic Status**	Open-ended question about current occupation	Developed by investigators
	Likert ladder with degree of participants socio-economic status (scaled 1 to 10, with 1 being the best off, most educated, most money, and the most respected job; at the bottom participants with less money, education, least respected jobs)	Adapted from The MacArthur Scale of Subjective Social Status[8]
	Household monthly income	Developed by investigators
**Data Quality**	4-items on interviewer-assessed quality of responses	Developed by investigators

1.UNICEF. Multiple Indicator Cluster Survey (MICS): Household Questionnaire [Internet]. 2017. Available from: http://mics.unicef.org/tools#survey-design
2.The DHS Program, USAID. Demographic and Health Surveys (DHS): Questionnaire Modules [Internet]. 2015. Available from: https://dhsprogram.com/publications/publication-dhsqm-dhs-questionnaires-and-manuals.cfm
3.WHO, UNICEF. Core Questions on Drinking Water and Sanitation for Household Surveys [Internet]. Geneva : World Health Organization; 2006. Available from: https://extranet.who.int/iris/restricted/handle/10665/43489
4.Boateng G, Collins SM, Mbullo P, Wekesa P, Onono M, Neilands TB, et al. A Novel Household Water Insecurity Scale: Procedures and Psychometric Analysis Among Postpartum Women in Western Kenya. Biorxiv 10.1101/294298.
5.Jepson WE, Wutich A, Collins SM, Boateng GO, Young SL. Progress in household water insecurity metrics: a cross-disciplinary approach. WIREs Water. 2017 Apr 11;4(3):e1214-21.
6.Coates J, Swindale A, Bilinsky P. Household food insecurity access scale (HFIAS) for measurement of food access: Indicator guide. Washington DC: Food and Nutrition Technical Assistance Project; 2007.
7.Cohen S, Kamarck T, Mermelstein R. A global measure of perceived stress. J Health Soc Behav. 1983;24(4):385–96.
8.Adler N, Stewart J, Psychosocial Working Group. The MacArthur Scale of Subjective Social Status [Internet]. MacArthur Scale of Subjective Social Status. 2007. Available from: http://www.macses.ucsf.edu/research/psychosocial/subjective.php
9.Wutich A, Brewis A. Food, Water, and Scarcity. Current Anthropology. 2014;55(4):444–68.
10.Jepson W. Measuring no-win waterscapes: Experience-based scales and classification approaches to assess household water security in *colonias* on the US--Mexico border. Geoforum. 2014;51(C):107–20.
11.Wutich A, Ragsdale K. Water insecurity and emotional distress: coping with supply, access, and seasonal variability of water in a Bolivian squatter settlement. Social science & medicine. 2008;67(12):2116–25.
12.Krumdieck N, Collins S, Wekesa P, Mbullo P, Boateng G, Onono M, et al. Household water insecurity is associated with a range of negative consequences among pregnant Kenyan women of mixed HIV status. Journal of Water and Health. 2016 Jul 25.

For peer review only - http://bmjopen.bmj.com/site/about/guidelines.xhtml

Household Water Insecurity Experiences

HWISE Data Protocol

Data cleaning procedure and notes for using the HWISE data

Purposes of Data Cleaning

I. Identify Aberrant Data

A. There are numerous instances in which data may be entered incorrectly or missing when it should not be; for each of the following situations, the protocol is to 1) contact PIs to see if the data can be recovered and/or corrected and, if not, 2) either update the aberrant data with a reasonable value or as missing

- Categorical variables that are not within reasonable or defined bounds (e.g. age is reported to be 500)
- Data that are logically incongruent (e.g. respondent reports that he/she does not treat water but then lists a non-zero amount spent on water treatment)
- Missing data

II. De-string Numeric Variables

A. String variables (i.e. those with with non-numeric characters) are updated to be numerical

III. Split Multiple-Select Variables

A. To make multiple-select responses analyzable, they are split into multiple responses (e.g. months of water shortage are split into 12 variables)

IV. Remove Duplicates

A. If multiple entries are submitted for the same participant, these are flagged and only the most up-to-date submission is retained

V. Recode Values

A. Values may be recoded to more clearly flag data that should later be changed to missing for analysis

- 999 = “Don’t know”
- 888 = “Not applicable”
- 555 = “Refuse to answer”

B. Multiple-select response options sometimes vary across sites (e.g. ‘1’ is piped water in one site but ‘7’ in another); to account for this in aggregated datasets, values are recoded to be harmonious across sites

VI. Label Data

A. Add variable labels to describe what a variable represents

For peer review only - http://bmjopen.bmj.com/site/about/guidelines.xhtml

B. Add value labels to categorical variables to convey what each value represents (e.g. '0' is male)

VII. Generate New Variables

A. Variables that are of broad interest to the group are generated based on other items within the dataset (e.g. creating a food insecurity score based on the FIAS questions)

VIII. Reorder Variables

A. Throughout the data entry and cleaning process, variables may be shifted around; these are reordered to match the order in the original survey

Stages of Data Cleaning

I. Cleaning of Site-Specific Data

A. Raw .csv data is imported and saved as a Stata dataset (.dta file)
B. All variables are then cleaned following the above guidelines; this includes variables unique to specific sites (e.g. ward in Ethiopia)
   - All changes made to aberrant data are documented in the data dictionary (see below)

C. Once cleaned, the dataset is saved

II. Cleaning of Aggregated Data

A. Cleaned datasets for each site are appended together
B. Site-specific variables are dropped
C. Multiple-select response options that vary across sites (e.g. '1' is piped water in one site but '7' in another) are recoded so responses across all sites are comparable
D. Questions related to money are converted to USD (exchange rate for each site based on date of last interview at the site and pulled from https://www.oanda.com/currency/converter/)
E. Once cleaned, the dataset is saved
F. Code replacing 555, 888, 999 with missing is then executed; the dataset is saved again

Using HWISE Data

I. Data Dictionary

For peer review only - http://bmjopen.bmj.com/site/about/guidelines.xhtml

A. The first tab ("Summary") provides a broad overview of the modules within the survey; blue text hyperlinks to the respective module in the more in-depth "Dictionary" tab
B. The "Dictionary" tab describes what each variables represents, data type, and the appropriate range of responses
C. The "Data Errors" section lists each error encountered while cleaning the site-specific data, as well as any corrective action taken; if you discover an error in the data that is not listed here, please contact the Northwestern Team

II. Determining Which Aggregated Dataset to Use

A. One dataset retains 555 (refuse to answer), 888 (not applicable), and 999 (don't know); this is best used for understanding the range of responses
B. The other dataset replaces 555, 888, and 999 with missing; it is best to use this dataset when performing analyses

For peer review only - http://bmjopen.bmj.com/site/about/guidelines.xhtml

Supplemental Material 4. Interviewer debriefing guide.

Cross-cultural study of Household Water Insecurity

Interviewer Debriefing Guide

Interviewer ID ____________________

Country __________________________ **Region/District**_________________

Gender of interviewer (participant): (0) Male (1) Female

Interviewer (participant) conducted interviews/surveys in what kind of area(s):
(0) Rural (2) Peri-urban (3) Urban

0a. This debrief is occurring after the completion of the:
(1) Cognitive interviews (2) Surveys

0b. Approximately how many of the above (surveys OR interviews) did you conduct? ________

0c. What was the primary language(s) you conducted the above (surveys or interviews) in? ____

1. What questions on the water insecurity access scale (WIAS) do you think worked best, and why?

2. Which questions on the water insecurity access scale (WIAS) were not well understood by participants? Why do you think they were hard to understand?

3. What questions didn't work in this population, and why?

4. What do you think was the most important question in understanding water insecurity here and why?

5. What question(s) do you wish we would have asked to better understand water insecurity?

For peer review only

For peer review only - http://bmjopen.bmj.com/site/about/guidelines.xhtml

BMJ Open

Development and validation protocol for an instrument to measure household water insecurity across cultures and ecologies: the Household Water InSecurity Experiences (HWISE) Scale

Journal:	*BMJ Open*
Manuscript ID	bmjopen-2018-023558.R2
Article Type:	Protocol
Date Submitted by the Author:	19-Oct-2018
Complete List of Authors:	Young, Sera; Northwestern University, Anthropology<br>Collins, Shalean ; Northwestern University, Anthropology<br>Boateng, Godfred; Northwestern University, Anthropology<br>Neilands, Torsten; University of California San Francisco, School of Medicine<br>Jamaluddine, Zeina; American University of Beirut, Center for Research on Population and Health<br>Miller , Joshua; Northwestern University, Anthropology<br>Brewis, Alex; Arizona State University, School of Human Evolution and Social Change<br>Frongillo, EA; University of South Carolina, Department of Health Promotion, Education, and Behavior<br>Jepson, Wendy; Texas A&M University Department of Geography<br>Melgar-Quiñonez, Hugo; McGill University Institute, Institute for Global Food Security<br>Schuster, Roseanne; Arizona State University, School of Human Evolution and Social Change<br>Stoler, JB; University of Miami, Department of Geography<br>Wutich, Amber; Arizona State University, School of Human Evolution and Social Change
**Primary Subject Heading**:	Global health
Secondary Subject Heading:	Research methods, Epidemiology, Public health
Keywords:	survey, water, household, scale development, water insecurity, protocol

SCHOLARONE™ Manuscripts

Supplemental Material 1. Overview of HWISE survey items, by implementation phase.

	HWISE 1.0	HWISE 2.0	Rationale for Modifications to HWISE 1.0
**Response Options:**	Never (0), Rarely (1), Sometimes (2), Often (3), Always (4)	Never (0), Rarely (1), Sometimes (2), Often (3), Always (4)	
**Number of Questions:**	32	30	
**Dimensions:**	Multidimensional: psychological, disease, nutrition, economic, social	Multidimensional: psychological, disease, nutrition, economic, social, cultural	
**Domains**	**HWISE 1.0**	**HWISE 2.0**	
**PSYCHOLOGICAL**	In the last 4 weeks, how frequently did you or anyone in your household **worry** you would not have **enough water for all of** your household needs?	In the last 4 weeks, how frequently did you or anyone in your household **worry** you would not have enough water for all of your household needs?	—
	In the last 4 weeks, how frequently have you or anyone in your household **worried about the safety** of the person getting water for your household?	In the last 4 weeks, how frequently have you or anyone in your household **worried about the safety of the person** getting water for your household?	—
	In the last 4 weeks, how frequently did you or anyone in your household **feel upset** about your water situation?	In the last 4 weeks, how frequently did you or anyone in your household feel **angry** about your water situation?	Word choice changed since upset does not translate well in many contexts and is tied to religiosity.
	In the last 4 weeks, how frequently has your or anyone in your household's **day been interrupted by your water situation**, including getting or distributing water within the household?	In the last 4 weeks, how frequently has you or anyone in your household had to **change schedules/plans** due to problems with your water situation, such as problems getting or distributing water within the household? Activities that may have been interrupted include caring for others, doing household chores, etc.	HWISE 1.0 items related to schedules subsumed into this question. Rephrased from "interrupted" to "changed schedules" because the former confused many participants in cognitive interviews.
		How **satisfied** are you with your water situation on a scale of 1-5? (1 is not at all satisfied and 5 is completely satisfied).	Starting with a question framed in the negative may bias people towards over-exaggerating their difficulties with water.
**SOCIAL**	In the last 4 weeks, how frequently have problems with water prevented you or anyone in your household from **attending social events** (i.e. church, funerals, community gatherings, etc.)?	In the last 4 weeks, how frequently have problems with water prevented you or anyone in your household from **attending social or cultural events** (e.g. church, funerals, community gatherings, cultural practices, etc.)?	Revised to include cultural components of water.
	In the last 4 weeks, how frequently did the **children** in your household **miss school** because they were getting water?	In the last 4 weeks, how frequently did the children in your household **miss school** or **go to school late** because of problems with water (e.g. time spent fetching water, lack of water for bathing, etc.)?	In many contexts, children only miss part of the school day as a consequence of water problems (e.g. time spent fetching water, cleanliness). analyses.
	In the last 4 weeks, how frequently did you or anyone in your household have problems with water that caused **difficulties with neighbors** or others in the community?	In the last 4 weeks, how frequently did you or anyone in your household have problems with water that caused **difficulties with neighbors, water providers, or others** in the community?	In many contexts, individuals face difficulties with their water providers.
	In the last 4 weeks, how frequently did you or anyone in your household have problems with water that caused **difficulties within your household**?	In the last 4 weeks, how frequently did you or anyone in your household have problems with water that caused **difficulties within your household**?	—
		In the last 4 weeks, how frequently have you or anyone in your household been unable to access the **water that you preferred**?	Previous version did not captures the concept of accessing a dignified, culturally-preferred water source.
		In the last 4 weeks, how frequently have problems with water caused you or anyone in your household to **feel ashamed**/excluded/stigmatized?	There are many uses and dimensions of water; currently, we were previously lacking a specific cultural component.
**ECONOMIC**	In the last 4 weeks, how frequently has the **time spent getting** water prevented you or anyone in your household from **earning money (**e.g. engaging in paid work, economic activities)?	In the last 4 weeks, how frequently have problems with water prevented you or anyone in your household from **earning money** (e.g. engaging in paid work, economic activities)?	Changed language to be applicable for flood and drought conditions.
	In the last 4 weeks, how frequently have you or anyone in your household **lacked money needed** to buy water?	In the last 4 weeks, how frequently have you or anyone in your household **lacked money** needed to **buy water**?	—
	In the last 4 weeks, how frequently have you or anyone in your household **thought of leaving [***name of town***]** because there was no water there?	In the last 4 weeks, how frequently have you or anyone in your household thought of **moving dwellings** because of the water situation there?	Language deemed too extreme; people tend not to leave town since they have family nearby but *are* likely to switch households. Also, rephrased to say water situation instead of no water, e.g. in case of flooding or contaminated water.
	In the last 4 weeks, how frequently did you or anyone in your household want to **buy water but there was nowhere to buy it from**?	In the last 4 weeks, how frequently did you or anyone in your household want to buy water but there was **nowhere to buy it from**?	—
	In the last 4 weeks, how frequently have you or anyone in your household asked to **borrow water** from other people?	In the last 4 weeks, how frequently have you or anyone in your household asked to **borrow** water from other people?	—
	In the last 4 weeks, how frequently have you or anyone in your household **loaned water** to anyone?	In the last 4 weeks, how frequently have you or anyone in your household **loaned water** to anyone? **Only asked in a subset of HWISE 2.0 sites*	—
**CARE/HEALTH**	In the last 4 weeks, how frequently has the **time spent getting** water prevented you or anyone in your household from **caring for children in the household**?		Subsumed under "change schedules/plans" in HWISE 2.0.
	In the last 4 weeks, how frequently has the **time spent getting** water prevented you or anyone in your household from **doing household chores** (such as cooking, preparing food, washing clothes, etc.)?		Subsumed under "change schedules/plans" in HWISE 2.0.
	In the last 4 weeks, how frequently has there **not been enough** water in the household to **wash clothes**?	In the last 4 weeks, how frequently has there not been enough water in the household to **wash clothes**?	—
	In the last 4 weeks, how frequently have you or anyone in your household had to go **without washing hands** after **dirty activities (**e.g., defecating or changing diapers, cleaning animal dung) because you didn't have enough water?	In the last 4 weeks, how frequently have you or anyone in your household had to go **without washing hands** after **dirty activities (**e.g., defecating or changing diapers, cleaning animal dung) because of problems with water?	Changed language to be applicable for flood and drought conditions.
	In the last 4 weeks, how frequently have you or anyone in your household not had enough water to **wash the faces and hands of children in your household?**	In the last 4 weeks, how frequently have you or anyone in your household not **washed the faces and hands of children** because of problems with water?	Changed language to account for flood events.
	In the last 4 weeks, how frequently have you or anyone in your household had to go without **washing their body** because there wasn't enough water?	In the last 4 weeks, how frequently have you or anyone in your household had to go without **washing their body** because of problems with water (e.g. not enough water, dirty, unsafe)?	Changed language to be applicable for flood and drought conditions.
	In the last 4 weeks, how frequently did you or anyone in your household want to **treat your water**, but couldn't? By treat, I mean boiling, using chemicals to treat, or other ways you make your water safe to use or drink.		Not many people experienced this.
	In the last 4 weeks, how frequently have you or anyone in your household **drank water** that **tasted bad**?	In the last 4 weeks, how frequently have you or anyone in your household drank water that **looked, tasted, and/or smelled bad**?	Participants mentioned that water can taste, look, and smell bad; the question is more encompassing by including all of these organoleptic properties.
	In the last 4 weeks, how frequently have you or anyone in your household **actually drank water** that you thought was unsafe?	In the last 4 weeks, how frequently have you or anyone in your household drank water that you thought was **unsafe**?	"Actually" does not add anything to the question, therefore removed.
	In the last 4 weeks, how frequently have you or anyone in your household **not had enough water to take medications**?		Not enough people experienced this; too specific.
	In the last 4 weeks, how frequently have you or anyone in your household not gotten water where you wanted to because you were **too sick or weak** to get water?		Too rare.
**NUTRITIONAL**	In the last 4 weeks, how frequently have you or anyone in your household had to **change what was being eaten** because there wasn't enough water (e.g. for washing foods, cooking, etc.)?	In the last 4 weeks, how frequently have you or anyone in your household had to **change what was being eaten** because there were problems with water (e.g. for washing foods, cooking, etc.)?	Changed language to be applicable for flood and drought conditions.
	In the last 4 weeks, how frequently has there not been as much water to drink as you would like for you or anyone in your household?	In the last 4 weeks, how frequently has there not been **as much water to drink** as you would like for you or anyone in your household?	—
	In the last 4 weeks, how frequently have you or anyone in your household gone to **sleep thirsty**?	In the last 4 weeks, how frequently have you or anyone in your household gone to **sleep thirsty** because there wasn't any water to drink?	Ensures that the question is measuring lack of water.
	In the last 4 weeks, how frequently has there been **no water whatsoever** in your household?	In the last 4 weeks, how frequently has there been **no useable or drinkable water** whatsoever in your household?	Changed language to be applicable for flood and drought conditions.
**AGRI-CULTURAL**	In the last 4 weeks, how frequently has your household **not had enough** water for your **garden, crops, or fruit trees**?	In the last 4 weeks, how frequently has your household water situation impacted the cultivation of your **garden, crops, or fruit trees**?	Changed language to be applicable for flood and drought conditions.
	In the last 4 weeks, how frequently has your household **not had enough water** to give to your **animals and poultry?**	In the last 4 weeks, how frequently has your household water situation impacted your raising of **animals and poultry**?	Changed language to be applicable for flood and drought conditions.
**OTHER**	In the last 4 weeks, how frequently has your household **water supply** from your main water source **been interrupted?**	In the last 4 weeks, how frequently has your household water supply from your main water source been **interrupted** or **limited** (e.g. water pressure, less water than expected)?	Include "limited" to cover instances where water is not available in normal or anticipated quantities.

HWISE 2.0 Consent Script, Survey, and Sources

Household Water Insecurity Experiences

Version 25, June 13, 2018

For peer review only - http://bmjopen.bmj.com/site/about/guidelines.xhtml

Household Water Insecurity Experiences

HWISE Consent Script

You are being asked to take part in a research study about the different factors affecting water insecurity and health for people in your area. We are asking you to take part because you live in this area and have unique insight into how water is acquired and used in your community.

What this study is about: The purpose of this study is to learn about the various ways people acquire and use water and any consequences that arise from household water insecurity. From this study, we will be able to develop a scale to measure household water insecurity in many settings across the world.

What we will ask you to do: If you agree to be in this study, we will ask you to participate in a brief survey during which we will ask you questions about the composition of your household, water acquisition and use, food insecurity, and stress.

Risks and benefits: There is the risk that you may find some of the questions about your household to be sensitive. There are no immediate benefits to you, but the information learned in this study will be useful for researchers who are studying household water insecurity. The information we learn through this survey may be shared with researchers at other institutions, but your personal identifying information will not be shared or linked to your responses.

Compensation. There is no compensation for taking part in this study.

Your answers will be confidential. We will write or record your responses to this survey using tablets, your name and information will not be included in any part of the survey. The written records and any information you share will be kept private. In any sort of report we make public we will not include any information that will make it possible to identify you. Research records will be kept in a locked file or in secure, password-protected online storage; only researchers will have access to the records.

Taking part is voluntary. Taking part in this study is completely voluntary. You may skip any questions that you do not want to answer. If you decide not to take part or to skip some of the questions, it will not affect your future relationship with Arizona State University or Northwestern University. If you decide to take part, you are free to withdraw at any time.

If you have questions: The lead researcher conducting this study is Dr. Sera Young at Northwestern University. If you have questions later, you can contact Dr. Young at sera.young@northwestern.edu. If you have any questions or concerns regarding your rights as a subject in this study, you may contact the Northwestern University Institutional Review Board (IRB) at +1 (847) 467-1723 or http://www.northwestern.edu.

Now I would like to ask if you agree to participate in this study.
Do you agree to participate in this study? **YES/NO**
Do you agree to allow me to record your responses to questions? **YES/NO**

Household Water Insecurity Experiences

HWISE 2.0 Survey

Cross-cultural study of household water insecurity	
Interviewer____	Date__________________
Time at start of interview____________________	Time at end of interview____________________
Country ___________________	Region/District__________________
Neighborhood__________________	
Participant ID:_______________	Language of interview: _________________
Participant gender: (0) Male (1) Female	
Participant place of residence: (0) Rural (2) Peri-urban (3) Urban	
Participant ethnicity:	
Participant ID should start with 1st two letters of country, eg. BA, NE, GU, GH…	

1. Screening Questions

Code Name	Questions	Coding Classification
SQ1a	Do you agree to participate in this survey?	0…No<br>1…Yes
SQ2	Are you 16 years of age or older?	0…No<br>1…Yes
SQ3	Would you consider yourself knowledgeable about water acquisition and use within your household?	0…No<br>1…Yes
SQ4	If no, who is most knowledgeable about water acquisition and use within your household? Who should I talk to?	

2. Socio Demographic Questions

SD1	Role in household: What is your relationship to the head of your household? By household, I mean all people who sleep under the same roof and take food from the same pot.	1….Self<br>2….Spouse/partner<br>3….Adult child<br>4….Other
SD2	What is the gender of household head?	0…Male<br>1…Female
SD9	What is your current relationship status?	1…Single/separated or divorced<br>2…Widowed<br>3…Have a partner who lives separately<br>4…Have a partner who lives with you<br>5…Married
SD3	How old are you?	
SD4	Who is primarily responsible for making sure there is enough water in the house? By household, I mean all people who sleep under the same roof and take food from the same pot.<br><br>*If primary responsibility is shared, choose (5) and then also circle the two categories of people who share the responsibility.*	1…Self<br>2…Spouse/partner<br>3…Children<br>4…Other family members<br>5…Shared responsibility

For peer review only - http://bmjopen.bmj.com/site/about/guidelines.xhtml

SD5	How many children (≤16 years) live in your household? By household, I mean all people who sleep under the same roof and take food from the same pot.	
SD6	How many adults (>16 years) including yourself live in your household? By household, I mean all people who sleep under the same roof and take food from the same pot.	
SD7	What type of housing do you live in?	1….House/condominium (owned)<br>2….House/condominium (rented)<br>3….Apartment (owned)<br>4…..Apartment (rented)<br>5….Farm (own)<br>6…..Farm (lease)<br>7…..Informal settlement/squatter community<br>8……Refugee/internally displaced person camp<br>9…..Other ____________________
SD8	What is your religion?	1….Christianity<br>2….Judaism<br>3….Islam<br>4…..Hinduism<br>5…..Buddhism<br>6…..Nonreligious<br>7…...Other
SD8a	What is your denomination?	

**3. Household Water Insecurity Experiences Scale (32 questions)**<br>*Now I'm going to ask you about your own personal experiences with water. For each of the items, please indicate how many times within the past 4 weeks or 30 days. Interviewer, please mark the response that best corresponds with the coding classification. For example, if participants says 15 times, you need to mark/circle code 4.*		
**Code name**	**Question**	**Coding Classification**
HWISE1	How **satisfied** are you with your water situation on a scale of 1-5? (1 is not at all satisfied and 5 is completely satisfied).	1…Not at all satisfied<br>2<br>3<br>4<br>5...Completely satisfied
HWISE2	In the last 4 weeks, how frequently have you or anyone in your household been unable to access the **water that you preferred**?	1...Never (0 times in the last 4 weeks)<br>2...Rarely (1–2 times in the last 4 weeks)<br>3...Sometimes (3–10 times in the last 4 weeks)<br>4...Often (11-20 times in the last 4 weeks)<br>5….Always (More than 20 times in the last 4 weeks)

For peer review only - http://bmjopen.bmj.com/site/about/guidelines.xhtml

		99….Don't know<br>88…Not applicable/I don't have this
HWISE3	In the last 4 weeks, how frequently did you or anyone in your household **worry** you would not have enough water for all of your household needs?	1...Never (0 times in the last 4 weeks)<br>2...Rarely (1–2 times in the last 4 weeks)<br>3...Sometimes (3–10 times in the last 4 weeks)<br>4...Often (11-20 times in the last 4 weeks)<br>5….Always (More than 20 times in the last 4 weeks)<br>99….Don't know<br>88…Not applicable/I don't have this
HWISE4	In the last 4 weeks, how frequently have you or anyone in your household **worried about the safety of the person** getting water for your household? *By getting, I mean: traveling to, collecting the water, and returning with the water.*	1...Never (0 times in the last 4 weeks)<br>2...Rarely (1–2 times in the last 4 weeks)<br>3...Sometimes (3–10 times in the last 4 weeks)<br>4...Often (11-20 times in the last 4 weeks)<br>5….Always (More than 20 times in the last 4 weeks)<br>99….Don't know<br>88…Not applicable/I don't have this
HWISE5	In the last 4 weeks, how frequently has your household water supply from your main water source been **interrupted** or **limited** (e.g. water pressure, less water than expected)?	1...Never (0 times in the last 4 weeks)<br>2...Rarely (1–2 times in the last 4 weeks)<br>3...Sometimes (3–10 times in the last 4 weeks)<br>4...Often (11-20 times in the last 4 weeks)<br>5….Always (More than 20 times in the last 4 weeks)<br>99….Don't know<br>88…Not applicable/I don't have this
HWISE5a	If your supply was interrupted or limited, were these **expected** (announced/scheduled) or unexpected?	0…Unexpected<br>1…Announced/Scheduled
HWISE6	In the last 4 weeks, how frequently has your household water situation impacted the cultivation of your **garden, crops, or fruit trees**?	1...Never (0 times in the last 4 weeks)<br>2...Rarely (1–2 times in the last 4 weeks)<br>3...Sometimes (3–10 times in the last 4 weeks)<br>4...Often (11-20 times in the last 4 weeks)<br>5….Always (More than 20 times in the last 4 weeks)<br>99….Don't know<br>88…Not applicable/I don't have this
HWISE7	In the last 4 weeks, how frequently has your household water situation impacted your raising of **animals and poultry**?	1...Never (0 times in the last 4 weeks)<br>2...Rarely (1–2 times in the last 4 weeks)<br>3...Sometimes (3–10 times in the last 4 weeks)<br>4...Often (11-20 times in the last 4 weeks)

For peer review only - http://bmjopen.bmj.com/site/about/guidelines.xhtml

		5….Always (More than 20 times in the last 4 weeks)<br>99….Don't know<br>88…Not applicable/I don't have this
HWISE8	In the last 4 weeks, how frequently have problems with water prevented you or anyone in your household from **earning money** (e.g. engaging in paid work, economic activities)?	1...Never (0 times in the last 4 weeks)<br>2...Rarely (1–2 times in the last 4 weeks)<br>3...Sometimes (3–10 times in the last 4 weeks)<br>4...Often (11-20 times in the last 4 weeks)<br>5….Always (More than 20 times in the last 4 weeks)<br>99….Don't know<br>88…Not applicable/I don't have this
HWISE9	In the last 4 weeks, how frequently have you or anyone in your household **lacked money** needed to **buy water**?	1...Never (0 times in the last 4 weeks)<br>2...Rarely (1–2 times in the last 4 weeks)<br>3...Sometimes (3–10 times in the last 4 weeks)<br>4...Often (11-20 times in the last 4 weeks)<br>5….Always (More than 20 times in the last 4 weeks)<br>99….Don't know<br>88…Not applicable/I don't have this
HWISE10	In the last 4 weeks, how frequently did you or anyone in your household want to buy water but there was **nowhere to buy it from**?	1...Never (0 times in the last 4 weeks)<br>2...Rarely (1–2 times in the last 4 weeks)<br>3...Sometimes (3–10 times in the last 4 weeks)<br>4...Often (11-20 times in the last 4 weeks)<br>5….Always (More than 20 times in the last 4 weeks)<br>99….Don't know<br>88…Not applicable/I don't have this
HWISE11	In the last 4 weeks, how frequently did the children in your household **miss school** or **go to school late** because of problems with water (e.g. time spent fetching water, lack of water for bathing, etc.)?	1...Never (0 times in the last 4 weeks)<br>2...Rarely (1–2 times in the last 4 weeks)<br>3...Sometimes (3–10 times in the last 4 weeks)<br>4...Often (11-20 times in the last 4 weeks)<br>5….Always (More than 20 times in the last 4 weeks)<br>99….Don't know<br>88…Not applicable/I don't have this
HWISE12	In the last 4 weeks, how frequently has there not been enough water in the household to **wash clothes**?	1...Never (0 times in the last 4 weeks)<br>2...Rarely (1–2 times in the last 4 weeks)<br>3...Sometimes (3–10 times in the last 4 weeks)<br>4...Often (11-20 times in the last 4 weeks)<br>5….Always (More than 20 times in the last 4 weeks)<br>99….Don't know<br>88…Not applicable/I don't have this

For peer review only - http://bmjopen.bmj.com/site/about/guidelines.xhtml

HWISE13	In the last 4 weeks, how frequently have you or anyone in your household had to **change what was being eaten** because there were problems with water (e.g. for washing foods, cooking, etc.)?	1...Never (0 times in the last 4 weeks)<br>2...Rarely (1–2 times in the last 4 weeks)<br>3...Sometimes (3–10 times in the last 4 weeks)<br>4...Often (11-20 times in the last 4 weeks)<br>5….Always (More than 20 times in the last 4 weeks)<br>99….Don't know<br>88…Not applicable/I don't have this
HWISE14	In the last 4 weeks, how frequently have you or anyone in your household had to go **without washing hands** after **dirty activities** (e.g., defecating or changing diapers, cleaning animal dung) because of problems with water?	1...Never (0 times in the last 4 weeks)<br>2...Rarely (1–2 times in the last 4 weeks)<br>3...Sometimes (3–10 times in the last 4 weeks)<br>4...Often (11-20 times in the last 4 weeks)<br>5….Always (More than 20 times in the last 4 weeks)<br>99….Don't know<br>88…Not applicable/I don't have this
HWISE15	In the last 4 weeks, how frequently have you or anyone in your household not **washed the faces and hands of children** because of problems with water?	1...Never (0 times in the last 4 weeks)<br>2...Rarely (1–2 times in the last 4 weeks)<br>3...Sometimes (3–10 times in the last 4 weeks)<br>4...Often (11-20 times in the last 4 weeks)<br>5….Always (More than 20 times in the last 4 weeks)<br>99….Don't know<br>88…Not applicable/I don't have this
HWISE16	In the last 4 weeks, how frequently have you or anyone in your household had to go without **washing their body** because of problems with water (e.g. not enough water, dirty, unsafe)?	1...Never (0 times in the last 4 weeks)<br>2...Rarely (1–2 times in the last 4 weeks)<br>3...Sometimes (3–10 times in the last 4 weeks)<br>4...Often (11-20 times in the last 4 weeks)<br>5….Always (More than 20 times in the last 4 weeks)<br>99….Don't know<br>88…Not applicable/I don't have this
HWISE17	In the last 4 weeks, how frequently has you or anyone in your household had to **change schedules/plans** due to problems with your water situation, such as problems getting or distributing water within the household? Activities that may have been interrupted include caring for others, doing household chores, etc.	1...Never (0 times in the last 4 weeks)<br>2...Rarely (1–2 times in the last 4 weeks)<br>3...Sometimes (3–10 times in the last 4 weeks)<br>4...Often (11-20 times in the last 4 weeks)<br>5….Always (More than 20 times in the last 4 weeks)<br>99….Don't know<br>88…Not applicable/I don't have this
HWISE18	In the last 4 weeks, how frequently have problems with water prevented you or anyone in your household from **attending social or cultural events**	1...Never (0 times in the last 4 weeks)<br>2...Rarely (1–2 times in the last 4 weeks)<br>3...Sometimes (3–10 times in the last 4 weeks)<br>4...Often (11-20 times in the last 4 weeks)

For peer review only - http://bmjopen.bmj.com/site/about/guidelines.xhtml

	(e.g. church, funerals, community gatherings, cultural practices, etc.)?	5….Always (More than 20 times in the last 4 weeks)<br>99….Don't know<br>88…Not applicable/I don't have this
HWISE19	In the last 4 weeks, how frequently have you or anyone in your household drank water that **looked, tasted, and/or smelled bad**?	1...Never (0 times in the last 4 weeks)<br>2...Rarely (1–2 times in the last 4 weeks)<br>3...Sometimes (3–10 times in the last 4 weeks)<br>4...Often (11-20 times in the last 4 weeks)<br>5….Always (More than 20 times in the last 4 weeks)<br>99….Don't know<br>88…Not applicable/I don't have this
HWISE20	In the last 4 weeks, how frequently have you or anyone in your household drank water that you thought was **unsafe**?	1...Never (0 times in the last 4 weeks)<br>2...Rarely (1–2 times in the last 4 weeks)<br>3...Sometimes (3–10 times in the last 4 weeks)<br>4...Often (11-20 times in the last 4 weeks)<br>5….Always (More than 20 times in the last 4 weeks)<br>99….Don't know<br>88…Not applicable/I don't have this
HWISE21c	If you needed to borrow water, from how many people could you borrow water?	
HWISE21	In the last 4 weeks, how frequently have you or anyone in your household asked to **borrow** water from other people?	1...Never (0 times in the last 4 weeks)<br>2...Rarely (1–2 times in the last 4 weeks)<br>3...Sometimes (3–10 times in the last 4 weeks)<br>4...Often (11-20 times in the last 4 weeks)<br>5….Always (More than 20 times in the last 4 weeks)<br>99….Don't know<br>88…Not applicable/I don't have this
HWISE21a	From whom? Please list all the ways you are connected to these people. For example, neighbor, family member, or both neighbor and family member.	Person #1:<br><br>Person #2:<br><br>Person #3:
HWISE21b	What were you expected to give in return?	
HWISE30	In the last 4 weeks, how frequently have you or anyone in your household loaned water to anyone?	1...Never (0 times in the last 4 weeks)<br>2...Rarely (1–2 times in the last 4 weeks)<br>3...Sometimes (3–10 times in the last 4 weeks)<br>4...Often (11-20 times in the last 4 weeks)<br>5….Always (More than 20 times in the last 4 weeks)

For peer review only - http://bmjopen.bmj.com/site/about/guidelines.xhtml

		99….Don't know<br>88…Not applicable/I don't have this
HWISE22	In the last 4 weeks, how frequently did you or anyone in your household have problems with water that caused **difficulties with neighbors, water providers, or others** in the community?	1...Never (0 times in the last 4 weeks)<br>2...Rarely (1–2 times in the last 4 weeks)<br>3...Sometimes (3–10 times in the last 4 weeks)<br>4...Often (11-20 times in the last 4 weeks)<br>5….Always (More than 20 times in the last 4 weeks)<br>99….Don't know<br>88…Not applicable/I don't have this
HWISE23	In the last 4 weeks, how frequently did you or anyone in your household have problems with water that caused **difficulties within your household**?	1...Never (0 times in the last 4 weeks)<br>2...Rarely (1–2 times in the last 4 weeks)<br>3...Sometimes (3–10 times in the last 4 weeks)<br>4...Often (11-20 times in the last 4 weeks)<br>5….Always (More than 20 times in the last 4 weeks)<br>99….Don't know<br>88…Not applicable/I don't have this
HWISE24	In the last 4 weeks, how frequently did you or anyone in your household feel **angry** about your water situation?	1...Never (0 times in the last 4 weeks)<br>2...Rarely (1–2 times in the last 4 weeks)<br>3...Sometimes (3–10 times in the last 4 weeks)<br>4...Often (11-20 times in the last 4 weeks)<br>5….Always (More than 20 times in the last 4 weeks)<br>99….Don't know<br>88…Not applicable/I don't have this
HWISE25	In the last 4 weeks, how frequently has there not been **as much water to drink** as you would like for you or anyone in your household?	1...Never (0 times in the last 4 weeks)<br>2...Rarely (1–2 times in the last 4 weeks)<br>3...Sometimes (3–10 times in the last 4 weeks)<br>4...Often (11-20 times in the last 4 weeks)<br>5….Always (More than 20 times in the last 4 weeks)<br>99….Don't know<br>88…Not applicable/I don't have this
HWISE26	In the last 4 weeks, how frequently have you or anyone in your household gone to **sleep thirsty** because there wasn't any water to drink?	1...Never (0 times in the last 4 weeks)<br>2...Rarely (1–2 times in the last 4 weeks)<br>3...Sometimes (3–10 times in the last 4 weeks)<br>4...Often (11-20 times in the last 4 weeks)<br>5….Always (More than 20 times in the last 4 weeks)<br>99….Don't know<br>88…Not applicable/I don't have this
HWISE27	In the last 4 weeks, how frequently has there been **no useable or drinkable water** whatsoever in your household?	1...Never (0 times in the last 4 weeks)<br>2...Rarely (1–2 times in the last 4 weeks)<br>3...Sometimes (3–10 times in the last 4 weeks)

For peer review only - http://bmjopen.bmj.com/site/about/guidelines.xhtml

		4...Often (11-20 times in the last 4 weeks)<br>5….Always (More than 20 times in the last 4 weeks)<br>99….Don't know<br>88…Not applicable/I don't have this
HWISE28	In the last 4 weeks, how frequently have you or anyone in your household thought of **moving dwellings** because of the water situation there?	1...Never (0 times in the last 4 weeks)<br>2...Rarely (1–2 times in the last 4 weeks)<br>3...Sometimes (3–10 times in the last 4 weeks)<br>4...Often (11-20 times in the last 4 weeks)<br>5….Always (More than 20 times in the last 4 weeks)<br>99….Don't know<br>88…Not applicable/I don't have this
HWISE29	In the last 4 weeks, how frequently have problems with water caused you or anyone in your household to **feel ashamed**/excluded/stigmatized?	1...Never (0 times in the last 4 weeks)<br>2...Rarely (1–2 times in the last 4 weeks)<br>3...Sometimes (3–10 times in the last 4 weeks)<br>4...Often (11-20 times in the last 4 weeks)<br>5….Always (More than 20 times in the last 4 weeks)<br>99….Don't know<br>88…Not applicable/I don't have this

**4. Water Access**<br>*Now I would like to learn how and where you acquire water.*		
Wat1	What is currently the **primary source** of drinking water for your household? (Choose only ONE)	1…Piped water<br>2…Stand pipe<br>3…Borehole/tubewell<br>4…Protected dug well<br>5…Unprotected dug well<br>6…Protected spring<br>7…Unprotected spring<br>8…Rainwater collection<br>9…Small water vendor<br>10…Tanker truck<br>11…Bottled water<br>12…Bagged/sachet water<br>13…Surface water (pond, river, lake)<br>14…Other person ____________<br>15…Other_________
Wat2	What is currently the **primary source** of non-drinking water for your household? (Choose only ONE)	1…Piped water<br>2…Stand pipe<br>3…Borehole/tubewell<br>4…Protected dug well<br>5…Unprotected dug well<br>6…Protected spring<br>7…Unprotected spring<br>8…Rainwater collection

For peer review only - http://bmjopen.bmj.com/site/about/guidelines.xhtml

		9…Small water vendor<br>10…Tanker truck<br>11…Bottled water<br>12…Bagged/sachet water<br>13…Surface water (pond, river, lake)<br>14…Other person ____________<br>15…Other_________
Wat3	How long (in minutes) does it take to go to the water source, get water and come back (including wait time)? *(If water source is in household/compound, record 00 minutes)*	____________ minutes
Wat4	How many trips in total are made to this site per week (not including household/compound?)?	__________ trips
Wat4a	Have you ever been injured while fetching water?	0...No<br>1…Yes
Wat4b	If so, how?	
Wat5	In the past 4 weeks, approximately how much money did you spend on getting water for your household?	Units: ______ Amount: ________
Wat6	In the past 4 weeks, was drinking water for your household typically treated in any way to make it safer?	0…No<br>1…Yes<br>DK…Don't know
Wat7	What is the primary way that your household treats your **drinking** water?	1…Do not treat it<br>2…Boil<br>3…Filter<br>4…Add chemicals<br>5…Other (Specify):<br>___________________________
Wat8	In the past 4 weeks, how much money did you spend to treat water (including money for chemicals to treat water)?	Currency: ______ Amount: ________

only

**5. Water Quantity, Utility and Stability**<br>*Now I would like to ask you questions on the quantity of water you have in your household and the amount you drink.*		
Wat9	What is the estimate of the current amount of drinking water (liters) stored in your household?	________ Liters
Wat10	What is the estimate of the current amount of non-drinking water (liters) stored in your household?	________ Liters
Wat11	Which of the months in the year does your household mostly experience **water shortage** (circle all months)?	1…January 7…July<br>2…February 8…August<br>3…March 9…September<br>4…April 10…October<br>5…May 11…November

For peer review only - http://bmjopen.bmj.com/site/about/guidelines.xhtml

		6…June 12…December
Wat12	Which of the months in the year does your household have **excessive amounts** of water (tick the specific months)?	1…January 7…July<br>2…February 8…August<br>3…March 9…September<br>4…April 10…October<br>5…May 11…November<br>6…June 12…December
Wat13	What times of day does your household experience water shortages?	0…None<br>1…Morning (Sunrise (6:00am) to 11:59am)<br>2…Afternoon (12:00 – 5:00pm)<br>3…Evening (5:01 – 8:00pm)<br>4…Night (8:01pm – 5:59am)
Wat14	What do you see as the main **cause of problems with water** in your area?	
Wat15	What do you do when you **don't have enough water** and don't have enough money to buy water?	

6. Food Insecurity (HFIAS)

Now I'm going to ask you about your experiences with access to food in the last four weeks. I will ask you about the frequencies that you have experienced a few situations, and I want you to tell me how frequently it has happened in the last four weeks.

Code Name	Questions	Coding Classification
FI1	In the past four weeks, how frequently did you worry that your household would not have enough food?	1…Never (0 times in the last 4 weeks)<br>2…Rarely (1 – 2 times in the last 4 weeks)<br>3…Sometimes (3 – 10 times in the last 4 weeks)<br>4…Often (More than 10 times in the last 4 weeks)<br>9…Don't know
FI2	In the past four weeks, how frequently were you or any household member not able to eat foods you preferred because you couldn't obtain them because of a lack of resources (such as money, business, land, or any other thing that you would require to help you obtain other types of food)?	1…Never (0 times in the last 4 weeks)<br>2…Rarely (1 – 2 times in the last 4 weeks)<br>3…Sometimes (3 – 10 times in the last 4 weeks)<br>4…Often (More than 10 times in the last 4 weeks)<br>9…Don't know
FI3	In the past four weeks, how frequently did you or any household member have to eat a limited variety of food due to lack of resources (such as money, business, land, or any other thing that you would require to help you obtain other types of food)?	1…Never (0 times in the last 4 weeks)<br>2…Rarely (1 – 2 times in the last 4 weeks)<br>3…Sometimes (3 – 10 times in the last 4 weeks)<br>4…Often (More than 10 times in the last 4 weeks)<br>9…Don't know
FI4	In the past four weeks, how frequently did you or any household member have to eat some foods that you really did not want to eat because of a lack of resources to obtain other types of food?	1…Never (0 times in the last 4 weeks)<br>2…Rarely (1 – 2 times in the last 4 weeks)<br>3…Sometimes (3 – 10 times in the last 4 weeks)<br>4…Often (More than 10 times in the last 4 weeks)<br>9…Don't know
FI5	In the past four weeks, how frequently did	1…Never (0 times in the last 4 weeks)

For peer review only - http://bmjopen.bmj.com/site/about/guidelines.xhtml

	you or any household member have to eat a smaller meal than you felt you needed, meaning a little amount of food that did not satisfy you, because there was not enough food?	2…Rarely (1 – 2 times in the last 4 weeks)<br>3…Sometimes (3 – 10 times in the last 4 weeks)<br>4…Often (More than 10 times in the last 4 weeks)<br>9…Don't know
FI6	How many meals do you think you should eat in a day?	
FI7	In the past four weeks, how frequently did you or anyone in your household have to eat fewer meals in a day because there was not enough food?	1…Never (0 times in the last 4 weeks)<br>2…Rarely (1 – 2 times in the last 4 weeks)<br>3…Sometimes (3 – 10 times in the last 4 weeks)<br>4…Often (More than 10 times in the last 4 weeks)<br>9…Don't know
FI8	In the past four weeks, how frequently was there ever no food to eat of any kind in your household because of lack of resources to get food?	1…Never (0 times in the last 4 weeks)<br>2…Rarely (1 – 2 times in the last 4 weeks)<br>3…Sometimes (3 – 10 times in the last 4 weeks)<br>4…Often (More than 10 times in the last 4 weeks)<br>9…Don't know
FI9	In the past four weeks, how frequently did you or any household member go to sleep at night hungry because there was not enough food?	1…Never (0 times in the last 4 weeks)<br>2…Rarely (1 – 2 times in the last 4 weeks)<br>3…Sometimes (3 – 10 times in the last 4 weeks)<br>4…Often (More than 10 times in the last 4 weeks)<br>9…Don't know
FI10	In the past four weeks, how frequently did you or any household member go hungry for a whole day and night because of limited food in the house?	1…Never (0 times in the last 4 weeks)<br>2…Rarely (1 – 2 times in the last 4 weeks)<br>3…Sometimes (3 – 10 times in the last 4 weeks)<br>4…Often (More than 10 times in the last 4 weeks)<br>9…Don't know

7. Perceived Stress Scale

The questions in this scale ask you about your feelings and thoughts during the last month. In each case, indicate how often you felt or thought a certain way.

PS1	In the past four weeks, how often have you felt that you were unable to control the important things in your life?	1…Never (0 times in the last 4 weeks)<br>2…Almost Never (1-2 times in the last 4 weeks)<br>3…Sometimes (3-10 times in the last 4 weeks)<br>4…Fairly Often (11-20 times in the last 4 weeks)<br>5…Very Often (More than 20 times in the last 4 weeks)
PS2	In the last month, how often have you felt confident about your ability to handle your personal problems?	1…Never (0 times in the last 4 weeks)<br>2…Almost Never (1-2 times in the last 4 weeks)<br>3…Sometimes (3-10 times in the last 4 weeks)<br>4…Fairly Often (11-20 times in the last 4 weeks)<br>5…Very Often (More than 20 times in the last 4 weeks)
PS3	In the last month, how often have you felt that things were going your way?	1…Never (0 times in the last 4 weeks)<br>2…Almost Never (1-2 times in the last 4 weeks)<br>3…Sometimes (3-10 times in the last 4 weeks)<br>4…Fairly Often (11-20 times in the last 4 weeks)<br>5…Very Often (More than 20 times in the last 4 weeks)
PS4	In the last month, how often have you felt difficulties were piling up so high that you could not overcome them?	1…Never (0 times in the last 4 weeks)<br>2…Almost Never (1-2 times in the last 4 weeks)<br>3…Sometimes (3-10 times in the last 4 weeks)

For peer review only - http://bmjopen.bmj.com/site/about/guidelines.xhtml

		4…Fairly Often (11-20 times in the last 4 weeks)<br>5…Very Often (More than 20 times in the last 4 weeks)

**8. Infant feeding**		
This question will ask you about infant feeding and how it can be impacted by the water situation in your area.		
BF1	Can you tell me some ways that the water situation here affects how infants (under 12 months of age) are fed? (*Interviewer, prompt for three ways.)*	1.<br>2.<br>3.

**2a. Socio Demographic Questions (continued)**		
We are now going to ask you three final questions about your standing in your community.		
SD8	What is your current occupation or how do you earn money?	
SD9	Here is a picture of a ladder. Please think of this ladder as representing the socioeconomic standing of people ***in your community.***<br><br>At the top of the ladder are those who are best off; they have the most money, the most education, the most respected jobs. At the bottom are people who have the least money, least education, and least jobs.<br><br>Where would you place yourself on this ladder? Please touch the rung/step.<br><br>Interviewer, write the corresponding number here: ________	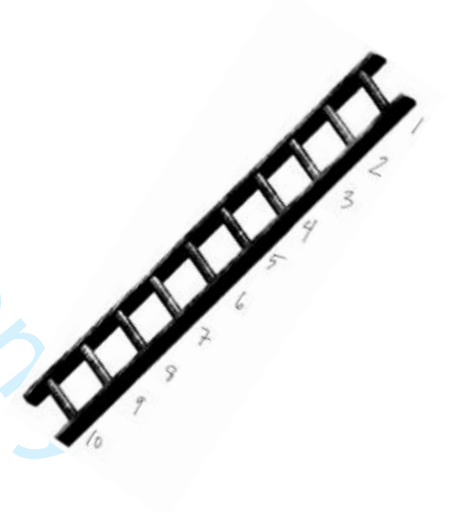

For peer review only

For peer review only - http://bmjopen.bmj.com/site/about/guidelines.xhtml

SD11	Here is a picture of a ladder. Please think of this ladder as representing the water situation of people ***in your community.***<br><br>At the top of the ladder are those who have the best water situation; they can easily get enough water for everything that they need and never have too much. At the bottom are people who have the most problems with water.<br><br>Where would you place yourself on this ladder? Please touch the rung/step.<br><br>Interviewer, write the corresponding number here: ________	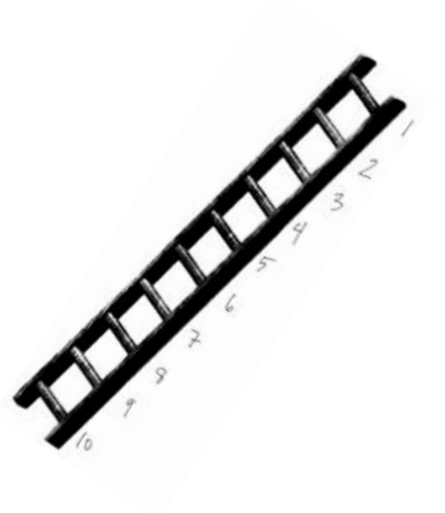
SD10	What is the primary monthly income for your household? (/month)	Units______ Amount ________

Wrap-up Question: Is there anything else you would like to share about your experiences with water for your household and how this affects your life?

Do you have any questions for us? ***Thank you*** *for participating in this survey.*

Data Quality: For the interviewer to complete<br>*Please give your assessment on the quality and reliability of the data you have collected in this survey.*		
DQ01	Did the respondent show any of the following? *(check all that apply):*	___ Mistrust of you or the study<br>___ Dishonesty, lying, or inventing responses that didn't seem true<br>___ Fear of you or the study<br>___ Hostility, anger or resentment<br>___ Evasion or trying to avoid answering
DQ02	Were there any interruptions or distractions?	0…No<br>1…Yes, but I don't think it influenced responses<br>2…Yes, and I think it influenced the answers
DQ03	What is your overall assessment of the quality of the data in this survey?	0…**Excellent**. The respondent understood the survey and was engaged and there were no interruptions<br>1…**Just okay**. The participant may not have understood all or part of the survey well or there were some interruptions.<br>2…**Suspect**. The participant misunderstood the survey or did not participate well or there were many interruptions.
DQ04	The quality of the data from this interview is really important to us. Please explain your answers above, in terms of how the data may have been affected.<br>Is there anything else we need to know?	

For peer review only - http://bmjopen.bmj.com/site/about/guidelines.xhtml

Household Water Insecurity Experiences

HWISE 2.0 Survey Sources

Topic	Brief Description	Source
Socio-demographics	Neighborhood, region, district/residence	Adapted from UNICEF Multiple Indicator Cluster Survey (MICS)[1]
	Role in household	
	Gender of Household head/respondent	
	Relationship status	Adapted from Demographic and Health Surveys (DHS) Household Questionnaire[2]
	Age of household head/respondent	
	Person responsible for collecting water in the household	Adapted from WHO & UNICEF Core Questions on Drinking Water and Sanitation for Household Surveys[3]
	Household size (# of adults & # of kids)	Adapted from UNICEF Multiple Indicator Cluster Survey (MICS)[1]
	Type of housing	Developed by investigators
	Religion and denomination	
Household Water Insecurity Experiences (HWISE) Scale	30-item household water insecurity experiences scale	Developed from extensive literature review[5] and team's knowledge of water insecurity[4,9-12]
Water sharing	Borrowing/loaning, what are you expected to give in return, from whom do you borrow?	Developed by investigators
Water Quality	Source of primary drinking water (WHO categories for improved & unimproved sources)	Adapted from WHO & UNICEF Core Questions on Drinking Water and Sanitation for Household Surveys[3]
	Source of primary non-drinking water (WHO categories for improved & unimproved sources)	
	Assessment of drinking water to be safe or unsafe	Developed by investigators
	Participants treating their water	Adapted from Demographic and Health Surveys (DHS) Household Questionnaire[2]
Water Accessibility	The amount of money spent by the household in water collection	Developed by investigators
	Estimate the time spent in collecting water from water source	Adapted from WHO & UNICEF Core Questions on Drinking Water and Sanitation for Household Surveys[3]
	Frequency of water collection	
Water Quantity	Amount of drinking water stored in household (L)	Developed by investigators
	Amount of non-drinking water stored in household (L)	
Water Utility	Amount of water drank in a day (L)	Developed by investigators
Water Stability/Reliability	Which of the months in a year do households experience water excess and scarcity?	Developed by investigators
	Which times of day do households experience water scarcity?	

For peer review only

**Food Insecurity**	9-item estimate via Household Food Insecurity Access Scale (HFIAS).	Adapted from the Household Food Insecurity Access Scale (HFIAS)[8]
**Perceived Stress**	4-item Estimate via Cohen's Perceived Stress Scale.	Adapted from the Perceived Stress Scale[7]
**Infant Feeding**	1 open-ended question on perceptions of how water insecurity may affect infant & young child feeding	Developed by investigators
**Socioeconomic Status**	Open-ended question about current occupation	Developed by investigators
	Likert ladder with degree of participants socio-economic status (scaled 1 to 10, with 1 being the best off, most educated, most money, and the most respected job; at the bottom participants with less money, education, least respected jobs)	Adapted from The MacArthur Scale of Subjective Social Status[8]
	Household monthly income	Developed by investigators
**Data Quality**	4-items on interviewer-assessed quality of responses	Developed by investigators

1.UNICEF. Multiple Indicator Cluster Survey (MICS): Household Questionnaire [Internet]. 2017. Available from: http://mics.unicef.org/tools#survey-design
2.The DHS Program, USAID. Demographic and Health Surveys (DHS): Questionnaire Modules [Internet]. 2015. Available from: https://dhsprogram.com/publications/publication-dhsqm-dhs-questionnaires-and-manuals.cfm
3.WHO, UNICEF. Core Questions on Drinking Water and Sanitation for Household Surveys [Internet]. Geneva : World Health Organization; 2006. Available from: https://extranet.who.int/iris/restricted/handle/10665/43489
4.Boateng G, Collins SM, Mbullo P, Wekesa P, Onono M, Neilands TB, et al. A Novel Household Water Insecurity Scale: Procedures and Psychometric Analysis Among Postpartum Women in Western Kenya. Biorxiv 10.1101/294298.
5.Jepson WE, Wutich A, Collins SM, Boateng GO, Young SL. Progress in household water insecurity metrics: a cross-disciplinary approach. WIREs Water. 2017 Apr 11;4(3):e1214-21.
6.Coates J, Swindale A, Bilinsky P. Household food insecurity access scale (HFIAS) for measurement of food access: Indicator guide. Washington DC: Food and Nutrition Technical Assistance Project; 2007.
7.Cohen S, Kamarck T, Mermelstein R. A global measure of perceived stress. J Health Soc Behav. 1983;24(4):385–96.
8.Adler N, Stewart J, Psychosocial Working Group. The MacArthur Scale of Subjective Social Status [Internet]. MacArthur Scale of Subjective Social Status. 2007. Available from: http://www.macses.ucsf.edu/research/psychosocial/subjective.php
9.Wutich A, Brewis A. Food, Water, and Scarcity. Current Anthropology. 2014;55(4):444–68.
10.Jepson W. Measuring no-win waterscapes: Experience-based scales and classification approaches to assess household water security in *colonias* on the US--Mexico border. Geoforum. 2014;51(C):107–20.
11.Wutich A, Ragsdale K. Water insecurity and emotional distress: coping with supply, access, and seasonal variability of water in a Bolivian squatter settlement. Social science & medicine. 2008;67(12):2116–25.
12.Krumdieck N, Collins S, Wekesa P, Mbullo P, Boateng G, Onono M, et al. Household water insecurity is associated with a range of negative consequences among pregnant Kenyan women of mixed HIV status. Journal of Water and Health. 2016 Jul 25.

For peer review only - http://bmjopen.bmj.com/site/about/guidelines.xhtml

Household Water Insecurity Experiences

HWISE Data Protocol

Data cleaning procedure and notes for using the HWISE data

Purposes of Data Cleaning

I. Identify Aberrant Data

A. There are numerous instances in which data may be entered incorrectly or missing when it should not be; for each of the following situations, the protocol is to 1) contact PIs to see if the data can be recovered and/or corrected and, if not, 2) either update the aberrant data with a reasonable value or as missing

- Categorical variables that are not within reasonable or defined bounds (e.g. age is reported to be 500)
- Data that are logically incongruent (e.g. respondent reports that he/she does not treat water but then lists a non-zero amount spent on water treatment)
- Missing data

II. De-string Numeric Variables

A. String variables (i.e. those with with non-numeric characters) are updated to be numerical

III. Split Multiple-Select Variables

A. To make multiple-select responses analyzable, they are split into multiple responses (e.g. months of water shortage are split into 12 variables)

IV. Remove Duplicates

A. If multiple entries are submitted for the same participant, these are flagged and only the most up-to-date submission is retained

V. Recode Values

A. Values may be recoded to more clearly flag data that should later be changed to missing for analysis

- 999 = "Don't know"
- 888 = "Not applicable"
- 555 = "Refuse to answer"

B. Multiple-select response options sometimes vary across sites (e.g. '1' is piped water in one site but '7' in another); to account for this in aggregated datasets, values are recoded to be harmonious across sites

VI. Label Data

A. Add variable labels to describe what a variable represents

For peer review only - http://bmjopen.bmj.com/site/about/guidelines.xhtml

B. Add value labels to categorical variables to convey what each value represents (e.g. '0' is male)

VII.Generate New Variables

A. Variables that are of broad interest to the group are generated based on other items within the dataset (e.g. creating a food insecurity score based on the FIAS questions)

VIII.Reorder Variables

A. Throughout the data entry and cleaning process, variables may be shifted around; these are reordered to match the order in the original survey

Stages of Data Cleaning

I. Cleaning of Site-Specific Data

A. Raw .csv data is imported and saved as a Stata dataset (.dta file)
B. All variables are then cleaned following the above guidelines; this includes variables unique to specific sites (e.g. ward in Ethiopia)
  - All changes made to aberrant data are documented in the data dictionary (see below)

C. Once cleaned, the dataset is saved

II. Cleaning of Aggregated Data

A. Cleaned datasets for each site are appended together
B. Site-specific variables are dropped
C. Multiple-select response options that vary across sites (e.g. '1' is piped water in one site but '7' in another) are recoded so responses across all sites are comparable
D. Questions related to money are converted to USD (exchange rate for each site based on date of last interview at the site and pulled from https://www.oanda.com/currency/converter/)
E. Once cleaned, the dataset is saved
F. Code replacing 555, 888, 999 with missing is then executed; the dataset is saved again

Using HWISE Data

I. Data Dictionary

For peer review only - http://bmjopen.bmj.com/site/about/guidelines.xhtml

A. The first tab ("Summary") provides a broad overview of the modules within the survey; blue text hyperlinks to the respective module in the more in-depth "Dictionary" tab
B. The "Dictionary" tab describes what each variables represents, data type, and the appropriate range of responses
C. The "Data Errors" section lists each error encountered while cleaning the site-specific data, as well as any corrective action taken; if you discover an error in the data that is not listed here, please contact the Northwestern Team

II. Determining Which Aggregated Dataset to Use

A. One dataset retains 555 (refuse to answer), 888 (not applicable), and 999 (don't know); this is best used for understanding the range of responses
B. The other dataset replaces 555, 888, and 999 with missing; it is best to use this dataset when performing analyses

For peer review only - http://bmjopen.bmj.com/site/about/guidelines.xhtml

Cross-cultural study of Household Water Insecurity

Interviewer Debriefing Guide

Interviewer ID ____________________

Country __________________________ **Region/District**_________________

Gender of interviewer (participant): (0) Male (1) Female

Interviewer (participant) conducted interviews/surveys in what kind of area(s):
(0) Rural (2) Peri-urban (3) Urban

0a. This debrief is occurring after the completion of the:
(1) Cognitive interviews (2) Surveys

0b. Approximately how many of the above (surveys OR interviews) did you conduct? ________

0c. What was the primary language(s) you conducted the above (surveys or interviews) in? _____

1. What questions on the water insecurity access scale (WIAS) do you think worked best, and why?

2. Which questions on the water insecurity access scale (WIAS) were not well understood by participants? Why do you think they were hard to understand?

3. What questions didn't work in this population, and why?

4. What do you think was the most important question in understanding water insecurity here and why?

5. What question(s) do you wish we would have asked to better understand water insecurity?

For peer review only

For peer review only - http://bmjopen.bmj.com/site/about/guidelines.xhtml
